# Forsythoside B alleviates cerebral ischemia-reperfusion injury via inhibiting NLRP3 inflammasome mediated by SIRT1 activation

**DOI:** 10.1371/journal.pone.0305541

**Published:** 2024-06-17

**Authors:** Qiaoyu Li, Chongyang Zhang, Xiao Sun, Mengchen Wang, Zhixiu Zhang, Rongchang Chen, Xiaobo Sun

**Affiliations:** 1 Key Laboratory of Innovative Drug Discovery of Traditional Chinese Medicine (Natural Medicine) and Translational Medicine, Institute of Medicinal Plant Development, Peking Union Medical College and Chinese Academy of Medical Sciences, Beijing, China; 2 Key Laboratory of Bioactive Substances and Resources Utilization of Chinese Herbal Medicine, Ministry of Education, Institute of Medicinal Plant Development, Chinese Academy of Medical Sciences & Peking Union Medical College, Beijing, China; 3 Key Laboratory of Efficacy Evaluation of Chinese Medicine Against Glycolipid Metabolic Disorders, State Administration of Traditional Chinese Medicine, Institute of Medicinal Plant Development, Peking Union Medical College and Chinese Academy of Medical Sciences, Beijing, China; 4 Guangdong Provincial Key Laboratory of Advanced Drug Delivery, Guangdong Pharmaceutical University, Guangzhou, China; National Institutes of Health, UNITED STATES

## Abstract

**Background:**

The inflammatory response is a key factor in the pathogenesis of cerebral ischemia/reperfusion injury (CIRI), and anti-inflammatory interventions may offer a promising therapeutic strategy. Forsythoside B (FB) is a phenylethanoid glycoside isolated from Forsythiae fructus, which has been reported to have anti-inflammatory effects. However, the mechanism of the neuroprotective effect of FB on CIRI remains unclear.

**Methods:**

Adult male Sprague-Dawley rats were subjected to transient middle cerebral artery occlusion/reperfusion (MCAO/R). FB was administered intraperitoneally for 3 days prior to MCAO/R. Cerebral infarct volume and neurological deficit score were used as indices to evaluate MCAO/R injury. The serum levels of inflammatory factors and antioxidant enzymes were measured. The activation of silent information regulator 2 homolog 1 (Sirt1) and the inhibition of the nucleotide-binding oligomerization domain-like receptor with a pyrin domain 3 (NLRP3) pathway were assessed through western blot and immunohistochemistry analysis. Furthermore, the rats were treated with Sirt1 shRNA 3 days before MCAO/R by stereotactical injection into the ipsilateral hemispheric region to assess the impact of Sirt1 knockdown on the protection of FB during MCAO/R.

**Results:**

FB reduced cerebral infarct volume and neurological deficit score in MCAO/R rats. FB reduced pathological changes and cell apoptosis in the hippocampal CA1 region and cortex on the ischemic side of rats. FB inhibited the serum levels of inflammatory factors and increased the activities of antioxidant enzymes. Further study showed that FB inhibited the activation of the NLRP3 pathway and induced Sirt1 activation.

**Conclusion:**

FB demonstrated neuroprotective and anti-inflammatory effects by inhibiting the NLRP3 pathway through Sirt1 activation in CIRI.

## Background

Stroke is an acute cerebrovascular disease and one of the leading causes of death and disability among adults worldwide [1], which is mainly divided into ischemic stroke and hemorrhagic stroke. Ischemic stroke is the most prevalent type of stroke, accounting for approximately 80% of all stroke cases [2]. It is characterized by the reduction of cerebral blood flow due to thromboembolic occlusion. Early restoration of cerebral blood supply is the main treatment strategy to improve the clinical prognosis of ischemic stroke [3]. However, the restoration of blood flow (reperfusion) after ischemia may further aggravate the cerebral injury, leading to what is known as cerebral ischemia/reperfusion injury (CIRI). Inhibiting CIRI is important for improving the prognosis of ischemic stroke [4]. The molecular mechanism of CIRI is complex and primarily involves the inflammatory response, mitochondrial energy metabolism disorder, excitatory amino acid toxicity, free radical injury, and pyroptosis [5]. All of these factors directly or indirectly contribute to neuronal death.

Inflammation is a common pathological process essential to CIRI [6]. The Nucleotide-binding oligomerization domain-like receptor protein 3 (NLRP3) inflammasome is a cytosolic protein complex comprising procysteinyl aspartate-specific protease-1 (pro-Caspase-1), apoptosis-associated speck-like protein (ASC), and NLRP3. It can detect cellular deviation from homeostasis as a danger signal and then trigger inflammatory responses. This complex is linked to a broad spectrum of diseases, including auto-inflammatory, infectious, and autoimmune diseases [7]. NLRP3 interacts with ASC and subsequently cleaves pro-Caspase-1. Activated Caspase-1 induces the maturation and secretion of interleukin (IL)-1β and IL-18. Previous studies have found that inhibition of nucleotide-binding oligomerization domain-like receptor protein 3 (NLRP3) pathway-mediated inflammation can significantly reduce CIRI [8, 9].

Oxidative stress is one of the most important factors for CIRI [10]. Reactive oxygen species (ROS) generation significantly increases during cerebral ischemia/reperfusion and can directly attack brain tissues, leading to mitochondrial dysfunction and apoptosis of neurons. Furthermore, ROS can increase the permeability of the blood-brain barrier, raising the risk of cerebral hemorrhage and brain edema [11]. What important, excessive generation and accumulation of ROS in brain tissue, induced by I/R, will activate adhesion molecules and promote immune cell infiltration, and subsequently induce inflammatory responses in both the peripheral immunological system and the central nervous system [12].

As a NAD^+^ dependent protein deacetylase, silent information regulator 2 homolog 1 (Sirt1) can regulate the acetylation of specific transcription factors and proteins, which is involved in various pathophysiological processes, including energy metabolism, stress response, inflammation, and redox homeostasis [13]. Emerging studies have shown that overexpression of Sirt1 significantly reduces brain damage in ischemic stroke by inhibiting oxidative stress and inflammation response [14, 15]. Sirt1 activation can inhibit the NLRP3 signaling pathway [16].

**Fig 1:**
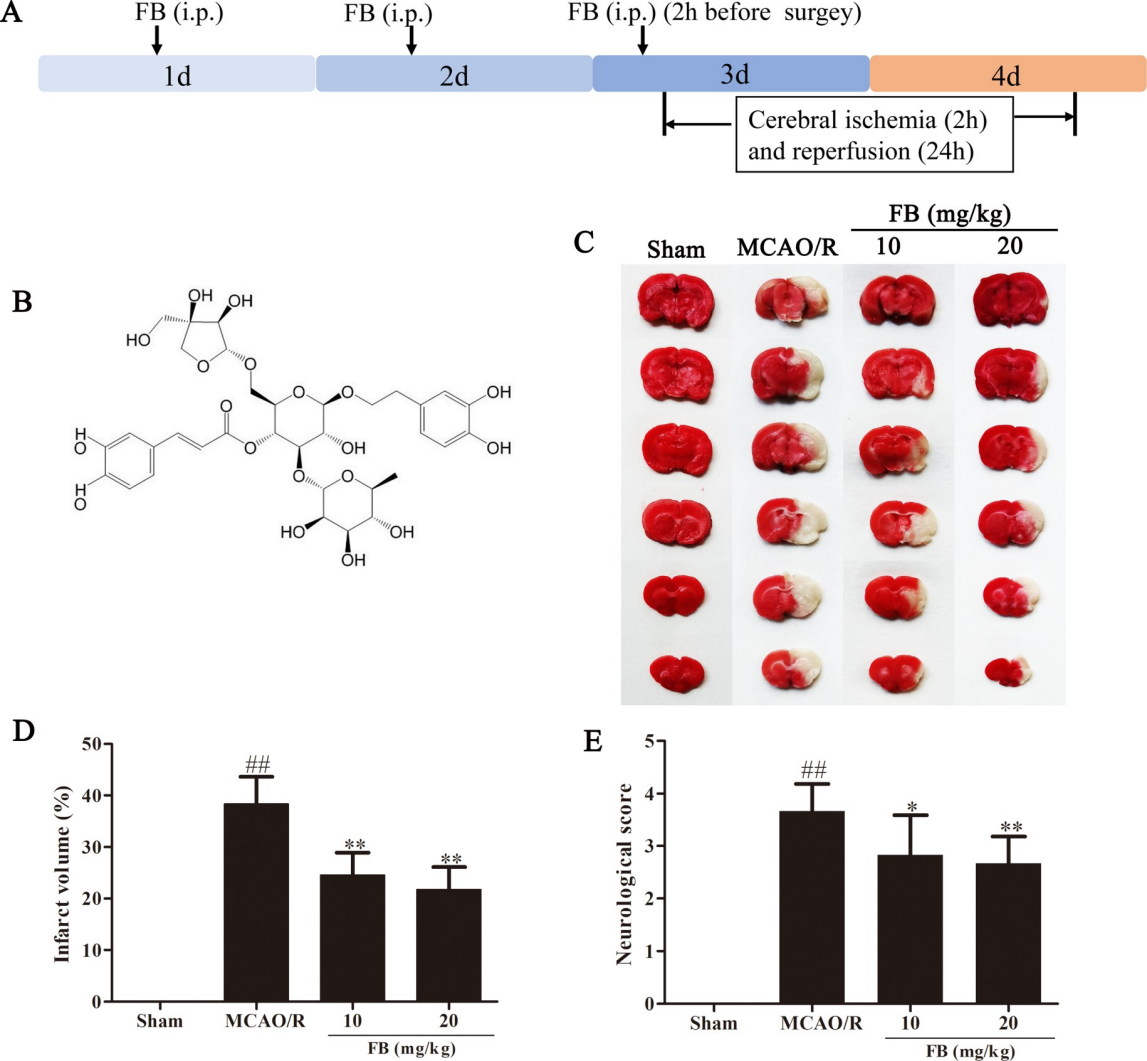
FB significantly reduced the volume of cerebral infarction and improved the behavioral indexes. (A) protocol for an in vivo study. (B) Chemical structure of FB. (C) Representative brain slices stained with TTC. (D) Quantitative evaluation of infarct volume. (E) Zea-Longa score results. n = 8 per group. All plots are presented as the mean ± SD. ^##^*p*<0.01 vs. the sham group; ^*^p<0.05, ^**^p<0.01 vs. the MCAO/R group.

Forsythiae Fructus (FF, Lianqiao in Chinese) is a traditional Chinese medicine, which is widely used as an antipyretic agent in China, Japan, and Korea [17, 18]. A lot of Chinese herbal preparations contain FF, such as Shuanghuanglian oral solution, Niuhuang Shangqing tablet, and Yinqiao Jiedu tablet, etc [19]. FF is rich in oleic acid and linoleic acid, which are easily absorbed and digested by the human body. The extract from FF can effectively inhibit the growth of common spoilage bacteria in the environment and prolong the shelf life of food. It is a promising, low-cost, and safe new food preservative. More than 30 phenylethanoid glycosides, which demonstrate anti-inflammatory, antibacterial, and antioxidant effects, have been extracted from FF [20]. Forsythoside B (FB) is a compound extracted from FF, which has the chemical structure C_34_H_44_O_19_ (Fig 1B) and exhibits superior neuroprotective effects in various diseases, including CIRI and Alzheimer’s disease [20, 21]. However, the mechanism behind the protective effect of FB on CIRI remains unclear. This study aims to investigate the neuroprotective effect of FB on CIRI using the rat MCAO/R model and explore its molecular mechanism, providing a basis for its development and utilization.

## Materials and methods

### Animal experiments and ethics statement

Male Sprague-Dawley (SD) rats, weighing 250–280 g, were purchased from Beijing Vital River Laboratories. All rat care and experimental procedures were approved by the Laboratory Animal Ethics Committee of the Institute of Medicinal Plant Development, Peking Union Medical College, and complied with the NIH Guidelines for the Care and Use of Laboratory Animals (approval number: SYXK 2021–0017). All rats were housed in ventilated cages at 22 ± 2°C with a 12-hour light/dark cycle. The rats were provided free access to rodent diet and tap water and subjected to adaptive feeding for one week.

The MCAO/R model was established as previously described by our research group [22]. After being anesthetized with an intraperitoneal injection of 2% sodium pentobarbital (60 mg/kg), the rats were fixed on an operating plate in the supine position. Then, the right common carotid artery (CCA) and external carotid artery (ECA) were identified and tied off. A silicone-coated 4–0 monofilament nylon suture with a rounded tip (Beijing Xinong Technology Co., Ltd, China) was inserted into the ICA through the ECA and gently pushed further to occlude the middle cerebral artery (MCA). Following 2 h of MCAO, 24 h reperfusion was induced by removing the suture after 24 hours. Sham-operated rats underwent the same surgical procedure, but the MCA was not occluded. During the operation, the rats were placed on a heating pad to maintain their body temperature at 37 ± 0.5°C. The experimenter was blinded to the treatment that the rats had received prior to all subsequent analyses.

### Drug treatment

FB was purchased from Shanghai Winherb Medical S&T Development (China). A total of 80 rats were randomly divided into four groups: (i) the sham group, (ii) the MCAO/R group, (iii) the group treated with 10 mg/kg of FB, and (iv) the group treated with 20 mg/kg of FB. FB was administered by intraperitoneally (i.p.). injection after dissolving in 0.9% normal saline for three times. Optimal administration times were 50 h, 26 h, and 2 h prior to model establishment. The sham and MCAO/R groups received equal volumes of physiological saline (Fig 1A).

### Neurological deficit score

Neurological deficit score was evaluated by two blinded investigators 24 h after reperfusion according to the Longa method [23]. Neurological deficit scores were recorded as follows: 0, no neurological deficit symptoms, normal activity; 1, the contralateral forelimb of the lesion could not be fully straightened; 2, rats turned to the opposite side when crawling; 3, rats walked their body to the opposite side; and 4, rats were unable to walk on their own, loss of consciousness.

### Infarct volume measurement

Intact brains were quickly removed after evaluating neurological deficits to determine the effect of FB on brain infarction. The brains were placed at -20°C for 15 min and then cut into six 2 mm thick coronal sections. The sections were incubated in a 2% TTC solution (Solarbio, China) at 37°C and then fixed in 4% paraformaldehyde overnight. Brain section images were captured using a digital camera (Nikon, Japan) and analyzed using Image-Pro Plus version 5.0 analysis software. Infarct areas from all sections were combined to calculate the total infarct area, which was then multiplied by the thickness of the brain sections to determine the infarct volume.

### Histopathology staining

Histopathological staining was conducted 24 hours after reperfusion, as previously described [24]. Briefly, brain tissues were fixed in 4% buffered paraformaldehyde, dehydrated in graded ethanol, and embedded in paraffin wax. The apex of the brain was sectioned and stained with H&E and Nissl (Solarbio, China). The structure was then examined under a light microscope (CKX41, Olympus, Tokyo, Japan) by a pathologist who was unaware of the groups being studied.

### Immunohistochemistry

Immunohistochemical staining of tissue sections was carried out following the method described in a previous study [24]. The sections were incubated at 60°C for 45 min followed by deparaffinization and rehydration. After soaking in 0.3% H_2_O_2_ (v/v) for 10 min at 37°C and being washed twice with double distilled water, the sections were placed into boiling citrate buffer (0.01M) for 2min for antigen repairing and then washed twice with phosphate-buffered saline (PBS) (Solarbio, China). Then, the coronal sections were blocked in 5% bovine serum albumin (Solarbio, China) for 1 h at room temperature. The sections were incubated at 4°C overnight in a humidified chamber with the following specific primary antibodies: anti-TNFα (1:200, Proteintech, USA), anti-caspase3 (1:100, Proteintech, USA), and anti-NLRP3 (1:200, Proteintech, USA). The slides were washed three times for 5 min each with PBS and then incubated with goat anti-rabbit IgG or goat anti-mouse IgG (Solarbio, China) for 1h at 37°C. Positive activity was revealed by 3–3′diaminobenzidine (DAB) (Solarbio, China). Finally, the slides were restained with hematoxylin, mounted, and observed under a light microscope (CKX41, Olympus, Tokyo, Japan). Quantification was performed using Image-Pro Plus 6.0 software (Media Cybernetics, USA). Protein expression levels were reflected by the mean optical density value (integrated optical density (IOD) divided by the relevant area).

### Transmission electron microscopy

Rats were sacrificed 24 h after MCAO, and the ischemic cerebral cortex and hippocampus of the animals were examined using transmission electron microscopy (TEM). In brief, the rats were anesthetized, and the tissue samples were harvested and fixed in 2.5% (w/v) glutaraldehyde (Beijing Sinouk institute of Biological Technology, China) for 30min. Then, the tissues were cut into small pieces of 1mm×1mm×1 mm and fixed in 2.5% (w/v) glutaraldehyde for more than 2h at 4°C. The specimens were fixed, dehydrated, soaked, embedded, solidified, and finally observed using TEM.

### Oxidative stress-related enzyme activities

Blood samples were collected from the abdominal aorta in heparinized tubes, allowed to clot for 30 min, and centrifuged at 3,000 rpm for 15 min at 4°C. The levels of superoxide dismutase (SOD), glutathione peroxidase (GSH-PX), catalase (CAT), and malondialdehyde (MDA) in the serum were determined using the corresponding kits (Nanjing Jiancheng Bioengineering Institute, China) according to the manufacturer’s protocols.

### Enzyme‑linked immunosorbent assay (ELISA) for inflammatory cytokines

The concentrations of IL18, IL1β, TNFα, MCP1, IL6, and ICAM1 in the serum were measured using rat-specific ELISA Kits according to the manufacturer’s protocol (Mlbio, China).

### Terminal Deoxynucleotidyl Transferase-mediated dUTP Nick-end Labeling (TUNEL) assay

Cell apoptosis in the ischemic penumbra of the cerebral was detected using the TUNEL apoptosis detection kit (Solarbio, China) following the manufacturer’s protocol. Briefly, the animals were anesthetized, the brain samples were removed and fixed in 4% paraformaldehyde. Subsequently, the samples were embedded in paraffin and sectioned on a coronal plane at a thickness of 5 μm. After dewaxing and rehydration, the sections were incubated with proteinase K (20 mg/mL) at room temperature for 15 min. The slices were washed with PBS and then incubated with working-strength terminal deoxynucleotidyl transferase enzyme at 37°C for 1 h in a moist chamber. After rinsing in a stop buffer, the sections were incubated with a working-strength antidigoxigenin conjugate for 30 min at room temperature. The slices were stained with DAPI and then subsequently examined under a fluorescence microscope (Leica, Heidelberg, Germany).

### Western blot

Tissues from the ischemic penumbra of rat brains were lysed on ice using tissue protein extraction reagent containing 0.1 mM dithiothreitol and a proteinase inhibitor cocktail (Beyotime, China). After centrifugation at 3000 rpm for 10min, the supernatant was collected, and the protein concentration was measured using a BCA kit (Beyotime, China). Protein SDS-PAGE loading buffer (Beyotime, China) was added, and the samples were heated to 100°C for 5 min to denature the proteins. Equal amounts of protein fractions were separated using 12% SDS-PAGE and then transferred onto nitrocellulose membranes (Millipore Corporation, USA) in Tris-glycine buffer at 100 V for 55 min. The membranes were blocked with 5% (w/v) nonfat milk powder in Tris buffer containing 0.05% (v/v) Tween-20 (TBST) (Cwbio, China) at room temperature for 2 h. The membrane was incubated with primary antibodies, including NLRP3, Caspase-1, IL-1β, Sirt1, and β-actin (Proteintech, USA), After overnight incubation at 4°C, the membranes were washed with TBST three times, the membranes were incubated with secondary antibodies (ZSGB-BIO, China) for 1 h at room temperature. Protein bands were visualized with enhanced chemiluminescence solution (Beyotime, China). The protein expression levels were visualized with Image Lab Software (Bio-Rad, USA). Quantification was performed using Image-Pro Plus 6.0 software (Media Cybernetics, USA).

### Sirt1 RNAi knockdown

Custom-made adeno-associated virus (AAV) harboring shRNA for rat Sirt1 (AAV-Sirt1) and rat nonsense control shRNA (AAV-Ctrl) were obtained from Hanbio Biotechnology Co. Ltd. (Shanghai, China). AAV-Sirt1 shRNA and AAV-Ctrl shRNA were injected into the ischemic cortex to knock down Sirt1 and as control, respectively, 3 days before MCAO/R, respectively. Briefly, the rats were anesthetized as previously described and then placed on a stereotactic apparatus. A total of 5μl lentiviruses were stereotaxically injected into the ischemic cortex at two sites on the right cortex (stereotaxic coordinates: A-P 1.0 mm, M-L -2.0 mm, D-V -1.2 mm; A-P -3.0 mm, M-L -1.5 mm, D-V -1.2 mm; 2.5μl for each site). The injection rate was 0.2μl/min, and the microinjector was left in place for 5 min before withdrawal.

A total of 80 rats were randomly divided into four groups: (i) AAV-Cont group, (ii) AAV-Cont + FB (20 mg/kg) group, (iii) AAV-Sirt1 group, and (iv) the AAV-Sirt1 + FB (20 mg/kg) group. After lentivirus administration, FB (20 mg/kg) treatment, MCAO/R model preparation, and index detection were conducted as previously described.

### Statistical analysis

All values are presented as mean ± SEM. Statistical analysis was conducted using one-way analysis of variance (ANOVA) followed by the Tukey test with GraphPad Prism software (GraphPad Software, San Diego, CA, USA). A value of P < 0.05 was considered to be statistically significant.

## Results

### FB decreased cerebral infarct volume and neurological deficit scores in MCAO/R rats

To examine the neuroprotective effect of FB in CIRI, we evaluated cerebral infarct volume and neurological score 24 h after reperfusion. Compared with the sham group, the rats in the MCAO/R group showed significantly higher cerebral infarct volume and neurological deficit scores. However, after FB (10 mg/kg and 20 mg/kg) intervention, the cerebral infarct volume and neurological deficit scores of the rats markedly decreased (Fig 1C–1E).

### FB reduced cerebral pathological injury in MCAO/R rats

**Fig 2:**
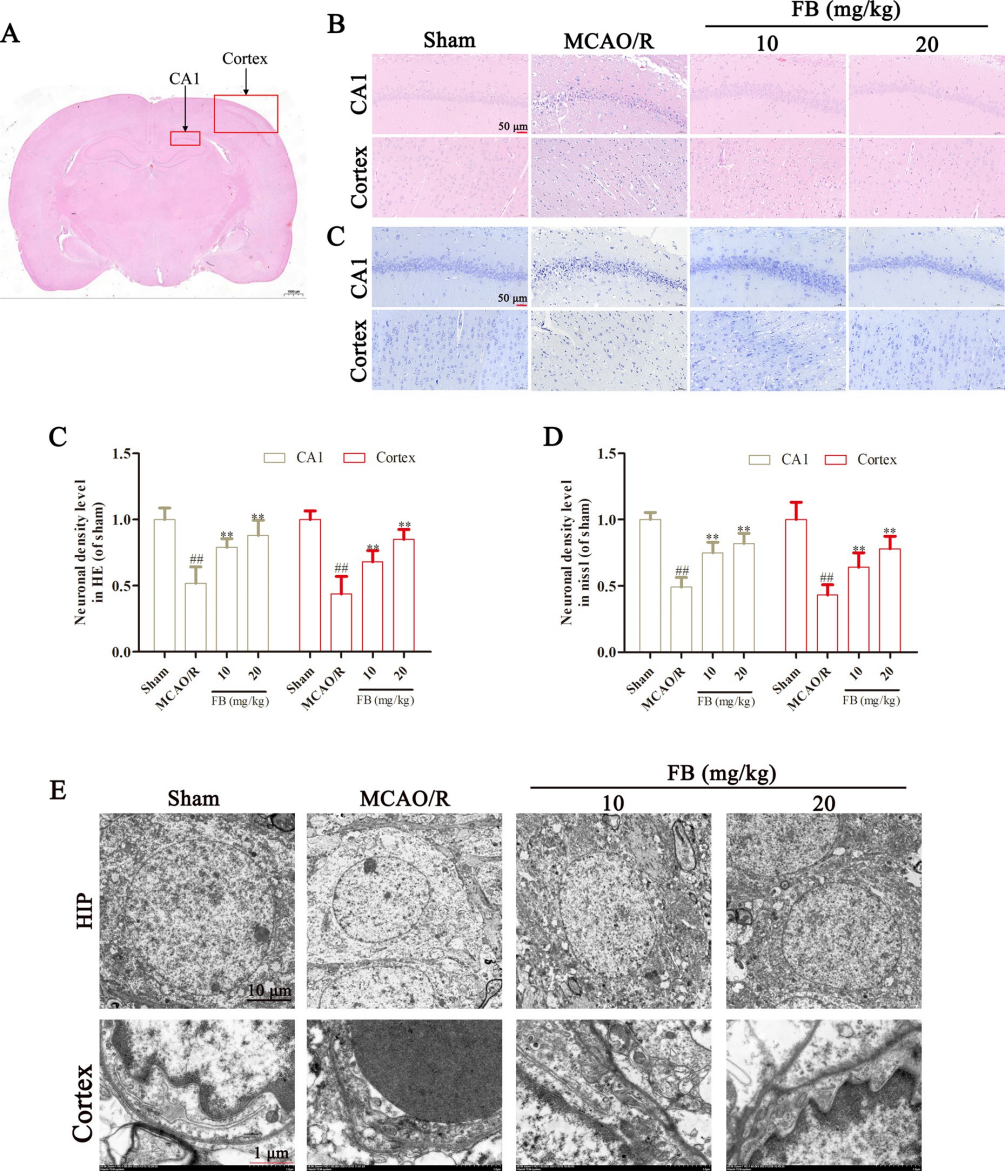
FB reduced cerebral pathological injury of MCAO rats. (A) The image positions corresponding to the cortex and the CA1 region of the hippocampus in each group. (B) Representative H&E and Nissl staining images in the cortex and the CA1 region of the hippocampus in each group. Scale bar = 50μm, n = 6. (C, D) Quantitative analysis of the H&E and Nissl staining. (E) Representative TEM images in the cortex and the CA1 region of the hippocampus in each group. n = 3. All plots are presented as the mean ± SD. ^##^*p*<0.01 vs. the sham group; ^**^p<0.01 vs. the MCAO/R group.

We then assessed the extent of injury in the hippocampal CA1 region and cortex on the ischemic side of rats using HE and Nissl staining. There were no obvious pathological changes observed in the rats of the sham group. Compared with the sham group, a significant number of neurons in the hippocampal CA1 region and cortex of rats in the MCAO/R group degenerated and became necrotic. The cell morphology was irregular and exhibited a disordered arrangement. However, FB pretreatment reduced neuronal damage and improved tissue morphology and structure, as indicated by the significantly increased neuron density (Fig 2A–2D).

From the electron microscope observation, we can see numerous electron-dense granules and vacuoles in the hippocampal of rats in the MCAO/R group. Swelling, dissolution, and rupture of various neuronal membrane structures were also observed. We examined the ultrastructure of cerebral microvascular endothelial cells in the cortex of rats and discovered that the vascular basement membrane was damaged. Additionally, the tight junctions between the capillary endothelial cells were disrupted, leading to greater opening in the MCAO/R group. FB pretreatment significantly reduced the ultrastructural damage of neurons and cerebral microvascular endothelial cells (Fig 2E).

### FB inhibited cell apoptosis in MCAO/R rats

**Fig 3:**
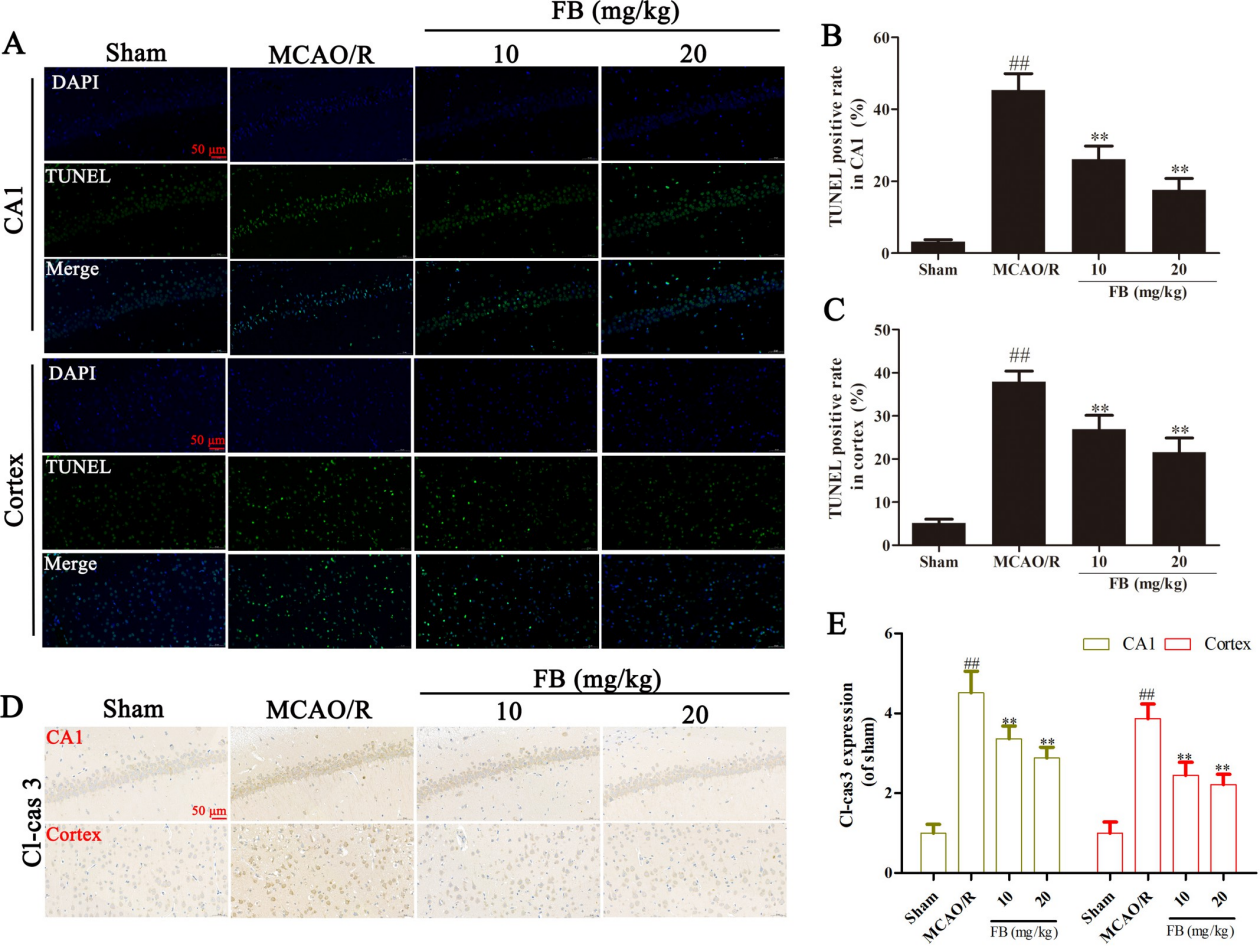
FB inhibited cell apoptosis in MCAO/R rats. (A) Representative TUNEL staining images in the cortex and the CA1 region of the hippocampus in each group. Scale bar = 50μm, n = 6. (B, C) Relative density (% of sham) of the TUNEL staining. (D) Immunohistochemical analysis of Cl-caspase 3 in the cortex and the CA1 region of the hippocampus in each group. n = 6. (E) Quantification of Cl-caspase 3 expression. All plots are presented as the mean ± SD. ^##^*p*<0.01 vs. the sham group; ^**^p<0.01 vs. the MCAO/R group.

We assessed the extent of cell apoptosis in the hippocampal CA1 region and cortex on the ischemic side of rats using TUNEL staining. The relative level of apoptosis in the MCAO/R group was significantly higher than that of the sham group. This increase was significantly inhibited by pretreatment with FB (10 mg/kg and 20 mg/kg) (Fig 3A–3C). Immunohistochemical analysis revealed an increase in Cl-caspase3-positive cells in the hippocampal CA1 region and cortex of the MCAO/R rats (Fig 3D and 3E). However, FB pretreatment inhibited the expression of Cl-caspase3.

### FB remitted the inflammation response and inhibited NLRP3 pathway in MCAO/R rats

**Fig 4:**
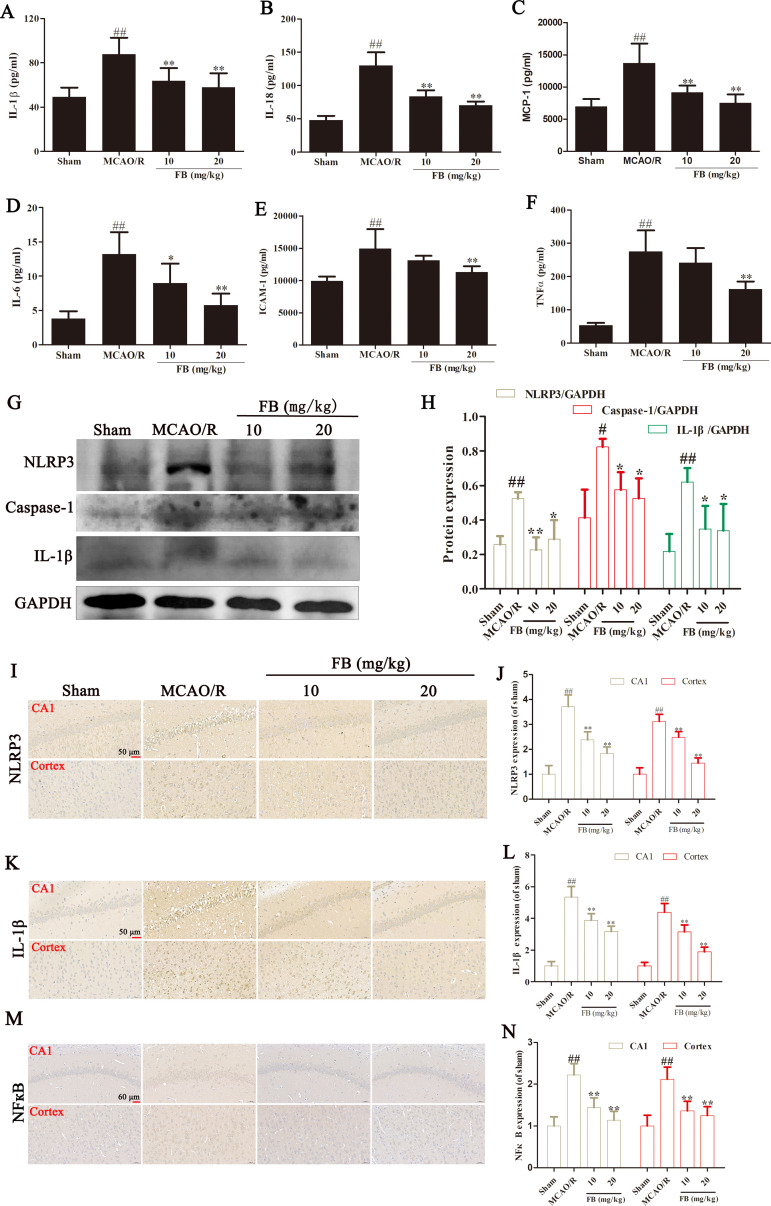
FB remitted the inflammation response and inhibited NLRP3 pathway in MCAO/R rats. (A, B, C, D, E, and F) The levels of IL-1β, IL-18, MCP-1, IL-6, ICAM-1, and TNF-α in serum were measured using ELISA kit. n = 8. (G) Protein expression of NLRP3, IL-1β, and caspase-1 in ischemic penumbra of rat brains were measured using western blot analysis. n = 3. (H) Quantification of protein expression. (I-N) Immunohistochemical analysis of NLRP3, IL-1β, and NFкB in the cortex and the CA1 region of the hippocampus in each group. n = 6. All plots are presented as the mean ± SD. ^##^*p*<0.01 vs. the sham group; ^*^p<0.01, ^**^p<0.01 vs. the MCAO/R group.

An ELISA assay was conducted to measure the levels of inflammatory cytokines. Serum levels of IL-1β, IL-18, MCP-1, IL-6, ICAM-1, and TNF-α in the MCAO/R rats significantly increased compared with those in the sham group. This increase was significantly inhibited by FB pretreatment (Fig 4A–4F).

To investigate whether the neuroprotective mechanism of FB is associated with the inhibition of the NLRP3 pathway, we assessed the impact of FB on the expression levels of NLRP3, Caspase-1, and IL-1β using western blot analysis. Results revealed a significant increase in the expression of Caspase-1, IL-1β, and NLRP3 in the MCAO/R rats (Fig 4G and 4H). Immunohistochemical analysis also indicated an increase in NLRP3 and IL-1β expression in the hippocampal CA1 region and cortex of the MCAO/R rats (Fig 4I–4L). However, FB pretreatment significantly inhibited the expression of Caspase-1, IL-1β, and NLRP3. We also detected NFкB expression in the hippocampal CA1 region and cortex of rats using immunohistochemical analysis. As shown in Fig 4M and 4N, NFкB expression increased significantly in MCAO/R rats and was inhibited by FB pretreatment.

### FB suppressed oxidative stress and increased Sirt1 expression in MCAO/R rats

**Fig 5:**
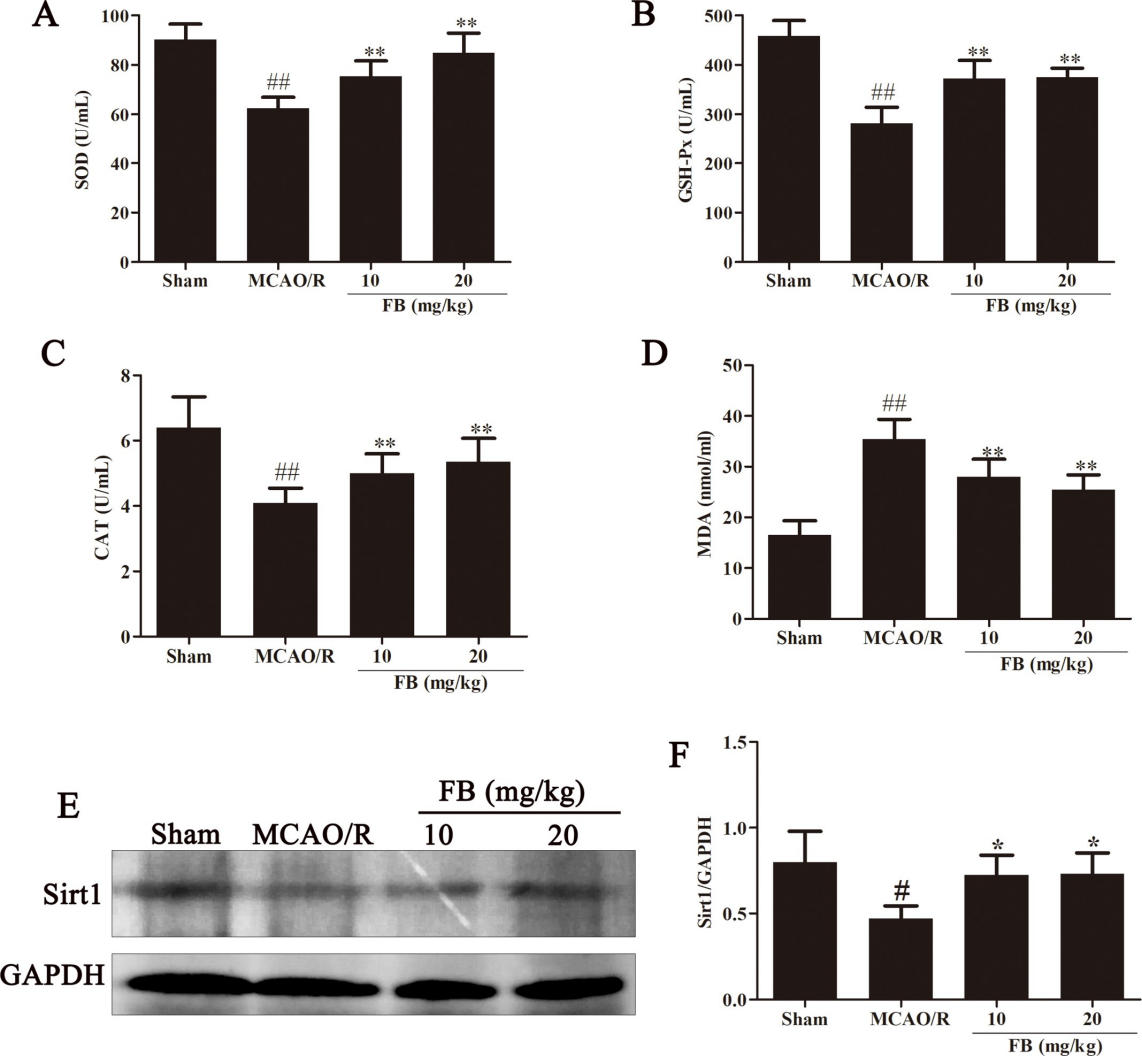
FB suppressed oxidative stress and increased Sirt1 expression in MCAO/R rats. (A, B, C, D) Serum levels of SOD, GSH-Px, CAT, and MDA in rats were measured. n = 8. (E) Protein expression of Sirt1 in ischemic penumbra of rat brains were assayed using western blot analysis. n = 3. (F) Quantification of protein expression. All plots are presented as the mean ± SD. ^##^*p*<0.01 vs. the sham group; ^**^p<0.01 vs. the MCAO/R group.

To evaluate the suppression of FB on oxidative stress in MCAO/R rats, we measured MDA levels, as well as the SOD, CAT, and GSH-Px activities in serum. MDA is a degradation product of membrane lipid oxidation, which is a primary event in oxidative damage. MDA levels in the MCAO/R group increased significantly, which was ameliorated by pretreatment with FB. Moreover, the activity of SOD, CAT, and GSH-Px decreased significantly in the MCAO/R group and was effectively increased by pretreatment with FB (Fig 5A–5D).

Sirt1 is involved in a variety of pathophysiological processes, including immune response and oxidative stress. Western blot analysis was utilized to quantify the level of Sirt1 in brain tissues. Compared with the sham group, the Sirt1 level in the MCAO/R group decreased significantly. Compared with the MCAO/R group, intervention with FB significantly increased Sirt1 expression. (Fig 5E and 5F).

### Sirt1 knockdown abolished the protective effect of FB on MCAO/R rats

**Fig 6:**
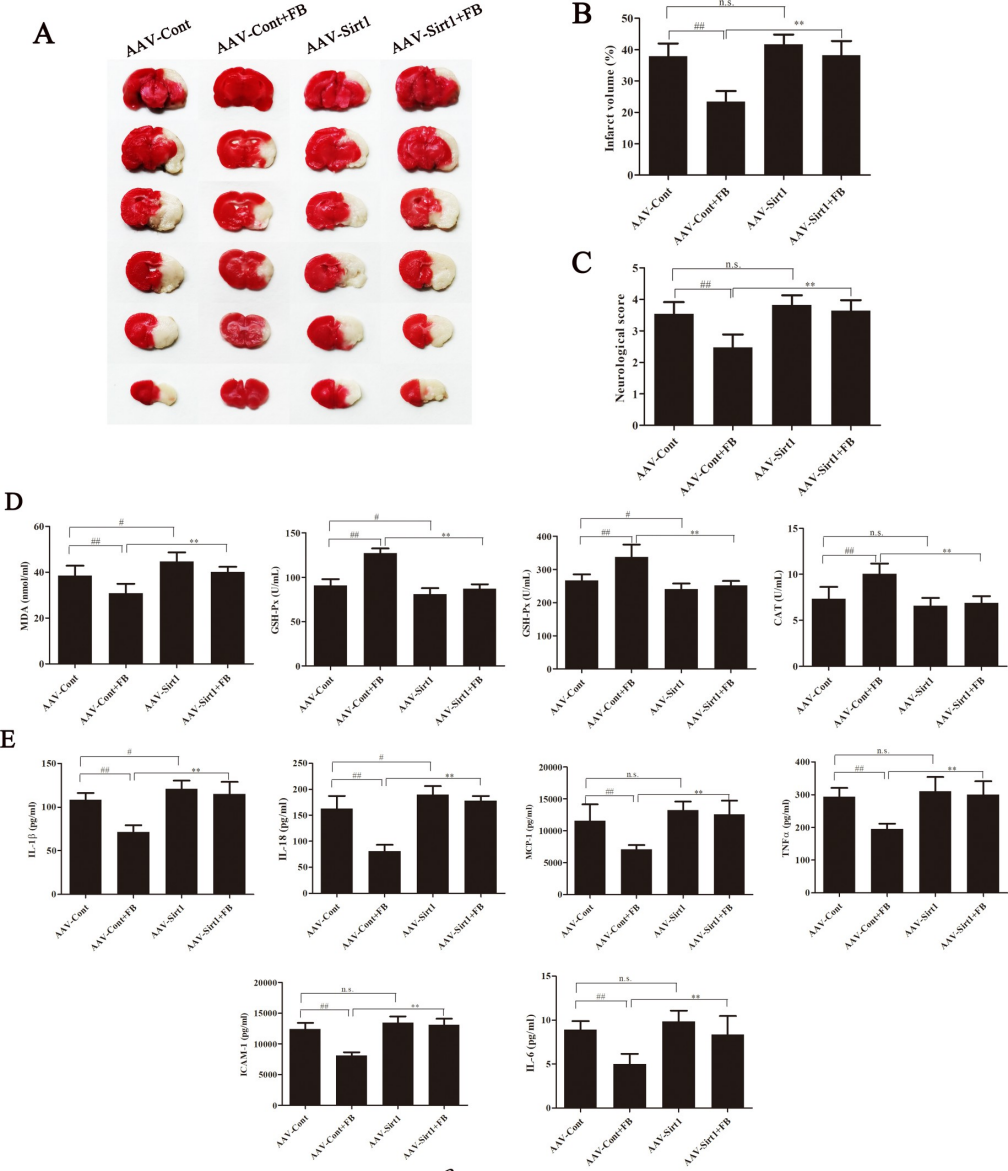
Sirt1 knockdown abolished the inhibition of FB on inflammatory response and oxidative stress in MCAO/R rats. (A) Representative brain slices stained by TTC. n = 6. (B) Quantitative evaluation of infarct volume. (C) Zea‑Longa score results. n = 8. (D) The levels of SOD, GSH-Px, CAT, and MDA in serum were measured using relevant reagent kits. n = 8. (E) The levels of IL-1β, IL-18, MCP-1, IL-6, ICAM-1, and TNF-a in serum of rats were measured by ELISA kits. n = 8. All plots are presented as the mean ± SD. ^#^*p*<0.05, ^##^*p*<0.01 vs. the AAV-Cont group; ^**^p<0.01 vs. the AAV-Cont+FB group.

To confirm the role of Sirt1 in the protective effect of FB on MCAO/R rats, rats were injected with AAV-Sirt1shRNA to knock down Sirt1 (AAV-Sirt1) in vivo. As a control, rats were injected with empty AAV control shRNA (AAV-Ctrl). The protective effect of FB on CIRI was assessed in genetically modified rats, with or without treatment using a 20 mg/kg dose of FB. In rats subjected to MCAO/R and treated with AAV control shRNA, FB treatment significantly reduced the brain infarct volume and improved neurological scores. However, in rats subjected to MCAO/R and treated with AAV-Sirt1 shRNA, no changes were observed (Fig 6A–6C). The protective effect of FB on MCAO/R rats was negated by Sirt1 knockdown.

### Sirt1 knockdown abolished the inhibition of FB on inflammatory response and oxidative stress in MCAO/R rats

We next evaluated whether Sirt1 participates in the regulatory effect of FB on inflammatory response and oxidative stress in MCAO/R rats. In rats subjected to MCAO/R and treated with AAV control shRNA, FB treatment significantly reduced the MDA level and increased the activities of SOD, CAT, and GSH-Px in serum (Fig 6D). Furthermore, FB treatment significantly reduced the levels of IL-1β, IL-18, MCP-1, IL-6, ICAM-1, and TNF-α in the serum (Fig 6E). However, in rats with MCAO/R that were treated with AAV-Sirt1 shRNA, no changes were observed. The inhibitory effect of FB on inflammatory response and oxidative stress in MCAO/R rats was eliminated by Sirt1 knockdown.

### Sirt1 knockdown abolished the inhibition of FB on NLRP3 pathway

**Fig 7:**
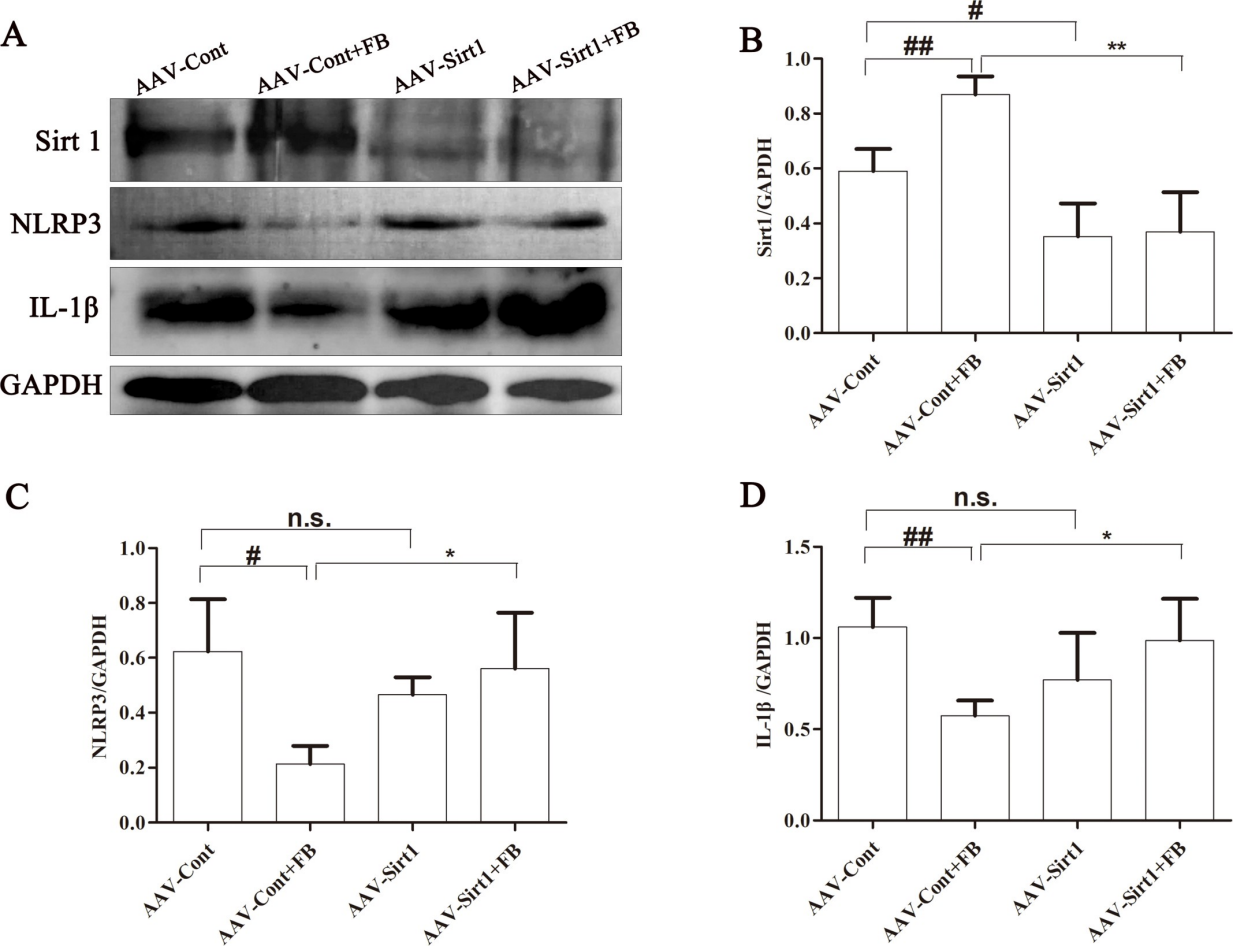
Sirt1 knockdown abolished the inhibition of FB on NLRP3 pathway. (A) Expression of Sirt1, NLRP3, and IL-1β in ischemic penumbra of rat brains were assayed by western blot analysis. (B, C, and D) Quantification of protein expression. n = 3. All plots are presented as the mean ± SD. ^#^*p*<0.05, ^##^*p*<0.01 vs. the AAV-Cont group; ^**^p<0.01 vs. the AAV-Cont+FB group.

To further investigate whether Sirt1 is involved in regulating the NLRP3 pathway by FB, we assessed the expression of related proteins in MCAO/R rats with and without Sirt1 knockdown. In rats subjected to MCAO/R and treated with AAV control shRNA, FB treatment significantly increased the expression of Sirt1 and decreased the expression of NLRP3 and IL-1β. However, in rats subjected to MCAO/R and treated with AAV-Sirt1 shRNA, no changes were observed. The inhibition of FB on the expression of NLRP3 inflammasome restriction mediated by Scu was abolished (Fig 7). These results indicate that FB may suppress the NLRP3 pathway by activating Sirt1.

## Discussion

Ischemic stroke has emerged as a significant threat to human health, and the potential for reperfusion injury in cerebral ischemic tissue has garnered the interest of scientists. In this study, we demonstrated that FB exerts a neuroprotective effect against CIRI by reducing the infarct volume and improving neurological scores. Furthermore, the FB administration reduced inflammation, oxidative stress, and neuronal apoptosis. Furthermore, the neuroprotective effect of FB was associated with NLRP3 inhibition and Sirt1 activation.

Accumulating evidence suggests that inflammation plays an important role in the pathogenesis of ischemic stroke. In ischemic stroke, the inflammatory cascade is activated immediately after the vessel occlusion [25]. The secondary inflammatory response is formed within a few minutes and can last for days or even weeks [26]. This process induces the production of a large number of proinflammatory factors, which lead to apoptosis of neuronal cells by causing vasomotor contraction, obstruction of microvessels, release of secreted cytotoxic enzymes, and destroying the integrity of the blood-brain barrier [27]. Therefore, inhibiting the production of inflammatory factors is essential for reducing CIRI. The present study revealed that FB treatment significantly inhibited the serum levels of IL-18, IL-1β, TNF-α, MCP-1, IL-6, and ICAM-1, indicating that the protective effect of FB on CIRI may be related to the inhibition of the inflammatory response.

As a crucial pathway in the inflammatory cascade, numerous studies have been conducted to explore the involvement of NLRP3 in CIRI [28]. The NLRP3 inflammasome in neurons drives neuroinflammation, and the inhibition or absence of NLRP3 reduces CIRI by decreasing the inflammatory response [29]. The results of the Western blot analysis in our study demonstrated that FB pretreatment reduced the protein expression of NLRP3 and caspase-1 in MCAO/R rats. This indicates that FB prevented cerebral injury and inflammatory response by inhibiting the NLRP3 signaling pathway. Nuclear transcription factor kappa B (NF-kB) controls the initiation and development of the underlying inflammatory reaction. The phosphorylated and activation of NFкb after ischemia promots the expression of pro-inflammatory cytokines (such as TNF-α and IL-1β). This pathological process leads to an excessive inflammatory response in the brain and causes serious damage including breakdown of the blood-brain barrier and brain edema in the early stage of CIR [30]. Therefore, blocking the NF-kB signaling pathway is an effective strategy for reducing inflammatory injury after stroke. The immunohistochemical results shown that FB decreased activation of NFкB in the brain MCAO/R rats, indicating that NFкB signaling pathway may also participate in the protection of FB on CIRI.

Under normal conditions, ROS can regulate several important physiological responses through redox-responsive signaling pathways [31]. When ROS generation is elevated, detrimental events are observed, including peculiar changes in cellular proteins, lipids, and ribonucleic acids, which can lead to cell dysfunction or death [32]. In rats with MCAO/R, ROS not only directly cause neural cell damage but also activate NLRP3 to stimulate the production of inflammatory factorss [33]. Therefore, inhibition of ROS production is important to reduce CIRI. Several enzymes with antioxidant activity are involved in neutralizing ROS, including SOD, GSH-Px, and CAT. Our results have demonstrated that FB pretreatment increased the activities of SOD, GSH-Px, and CAT, while decreasing the level of MDA in serum. These findings suggest that FB may protect against CIRI by enhancing the brain’s endogenous antioxidant capacity.

Sirt1 is a histone deacetylase that regulates a wide range of cellular functions, including immune response and redox stress [34]. Previous studies have shown that activating Sirt1 protects against various factors that cause neuronal injury and neurodegeneration, including CIRI [16]. The protective effect of Sirt1 on CIRI may be mediated by NLRP3 inhibition [16]. Furthermore, Sirt1 also inhibits oxidative stress [14]. Our study found that Sirt1 expression decreased in MCAO/R rats and was activated by FB pretreatment.

Sirt1-siRNA transfection was conducted to further investigate the role of Sirt1 in protecting against CIRI. Our experiments showed that knocking down Sirt1 abolished the protective effect of FB on CIRI. The increase in antioxidant enzymes and the decrease in inflammatory factors induced by FB was counteracted by Sirt1 knockdown. More importantly, in Sirt1 knockdown rats, FB was unable to increase Sirt1 expression, and its inhibitory effect on NLRP3, Caspase-1, and IL-1β disappeared. These results further demonstrate that Sirt1 plays a role in the neuroprotective effect of FB on MCAO/R rats by inhibiting the NLRP3 pathway and oxidative stress.

**Fig 8:**
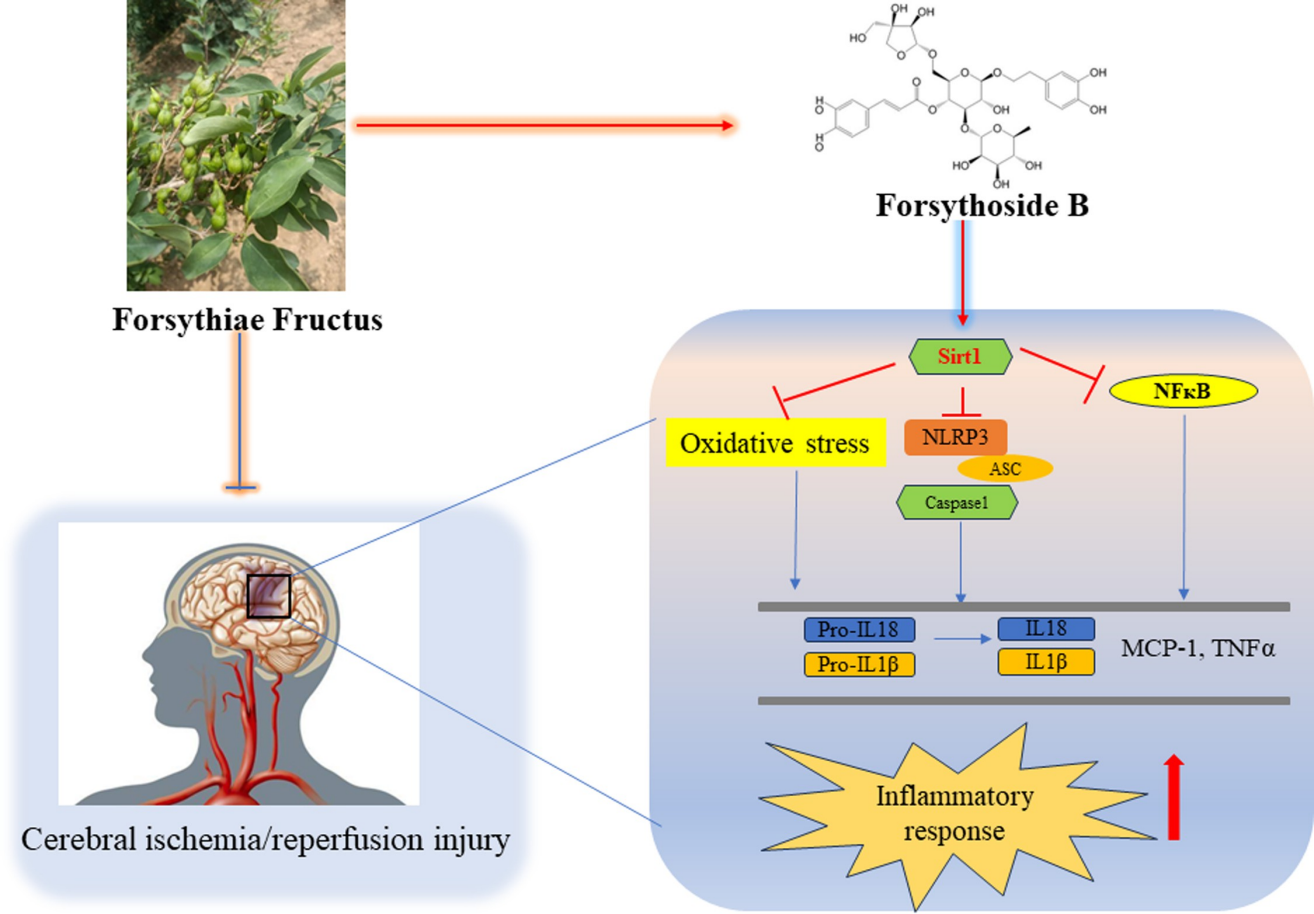
Illustration of the pharmacological effect and possible underlying mechanism of FB in protecting against CIRI.

In conclusion, our study suggests that FB has a neuroprotective effect against CIRI by inhibiting oxidative stress and the NLRP3-mediated inflammatory response, which is mediated by Sirt1 activation (Fig 8). The obtained results support the potential use of FB in the development of more effective therapeutic approaches to ischemic stroke.

## Supporting information

S1 Raw image (PDF)

S1 Raw data (ZIP)

## Abbreviations

CIRI: ischemia/reperfusion injury
FB: Forsythoside B
MCAO/R: middle cerebral artery occlusion/reperfusion
ROS: Reactive oxygen species
Sirt1: Silent information regulator 2 homolog 1
FF: Forsythiae Fructus
SD: Sprague-Dawley
CCA: Common carotid artery
ECA: External carotid artery
MCA: Middle cerebral artery
SOD: Superoxide dismutase
GSH-PX: Glutathione peroxidase
CAT: Catalase
MDA: Malondialdehyde
ELISA: Enzyme‑linked immunosorbent assay
TUNEL: Terminal Deoxynucleotidyl Transferase-mediated dUTP Nick-end Labeling
WB: Western blot
IF: Immunofluorescence
TNF: Tumor necrosis factor
FBS: Fetal bovine serum
i.p.: Intraperitoneal injection
SDS‑PAGE: Sodium dodecyl sulfate-polyacrylamide gel electrophoresis
ICA: Internal carotid artery
TTC: 2,3,5-Triphenyltetrazolium chloride
