# Supplementary material for: Forsythoside B alleviates cerebral ischemia-reperfusion injury via inhibiting NLRP3 inflammasome mediated by SIRT1 activation
Source: PLoS One. 2024 Jun 17;19(6):e0305541. doi: 10.1371/journal.pone.0305541 (PMC11182500; doi:10.1371/journal.pone.0305541)
Supplement: S1 Raw image — (PDF) [file pone.0305541.s001.pdf]

NLRP3 (Figure 4G)

The NLRP3 with no marker is more clearly, so we show NLRP3 with or without marker.

1 Sample with no marker

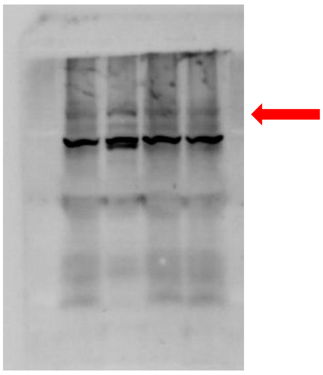

NLRP3-1

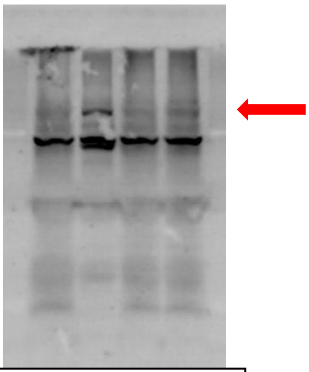

Used in Figure4G

NLRP3-2

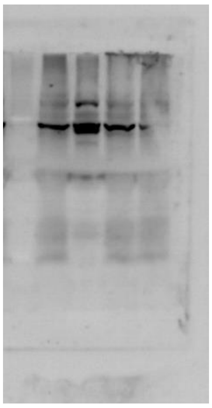

NLRP3-3

2 Sample with marker

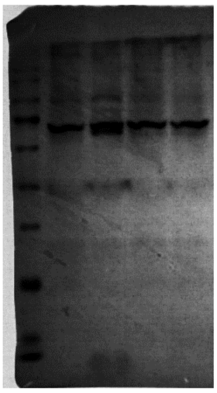

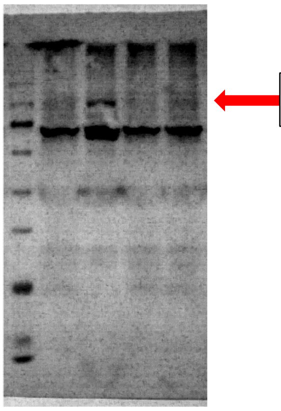

118Kd

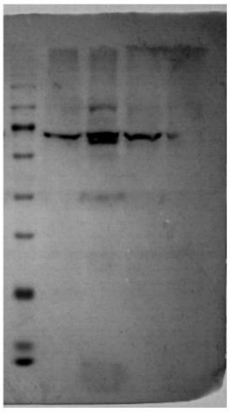

	NLRP3-1	NLRP3-2	NLRP3-3
lane 1	19311.4802	13400.31	14945.38
lane 2	27603.8944	31899.55	29812.24
lane 3	12954.3797	8008.309	16060.55
lane 4	12918.7731	9696.116	20342.67

Caspase-1 (Figure 4G)

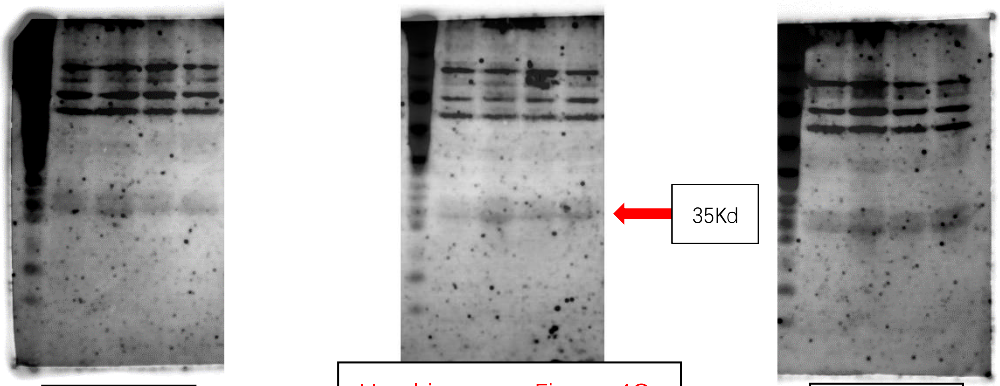

Cas1-1

Used in paper Figure 4G

Cas1-2

Cas1-3

	CAS1-1	CAS1-2	CAS1-3
lane 1	37022.9533	18304.29	20986.29
lane 2	49880.9447	44990.87	45794.41
lane 3	26492.2168	37558.36	29974.39
lane 4	19728.3087	31114.82	27246.72

IL-1β (Figure 4G)

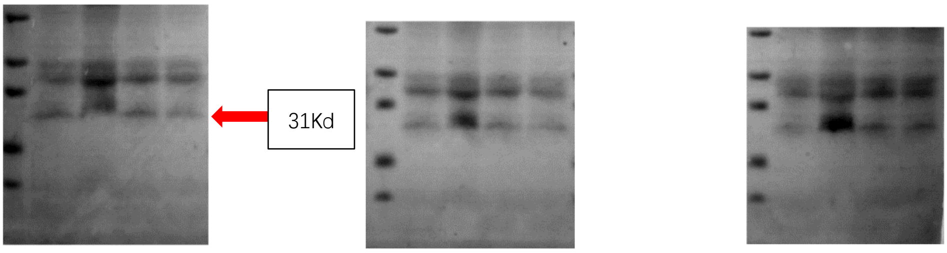

Used in Figure 4G

IL-1β-1

IL-1β-2

IL-1β-3

	IL-1β-1	IL-1β-2	IL-1β-3
lane 1	7378.5097	19928.14	12933.14
lane 2	38191.9863	29989.24	37626.97
lane 3	11244.8234	20309.31	25605.07
lane 4	8924.9949	17007.09	24328.14

Sirt1 (Figure 5E)

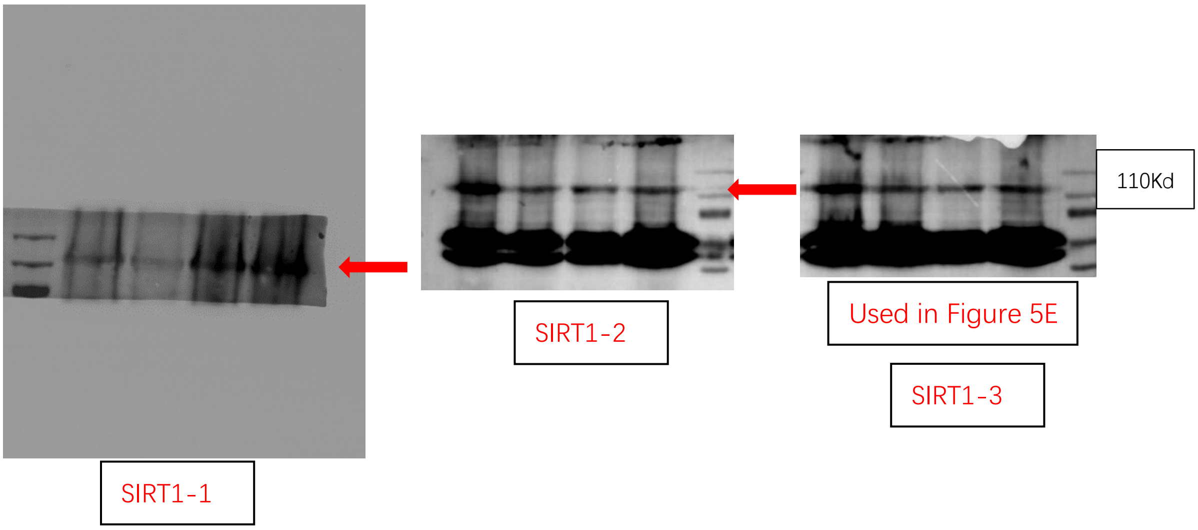

	SIRT1 -1	SIRT1 -2	SIRT1 -3
lane 1	36280.3711	55444.96	55841.4
lane 2	21987.3087	28209.67	30103.06
lane 3	46749.0574	35118.16	36719.74
lane 4	43071.4005	31380.18	34318.76

GAPDH (Figure 4G and 5E)

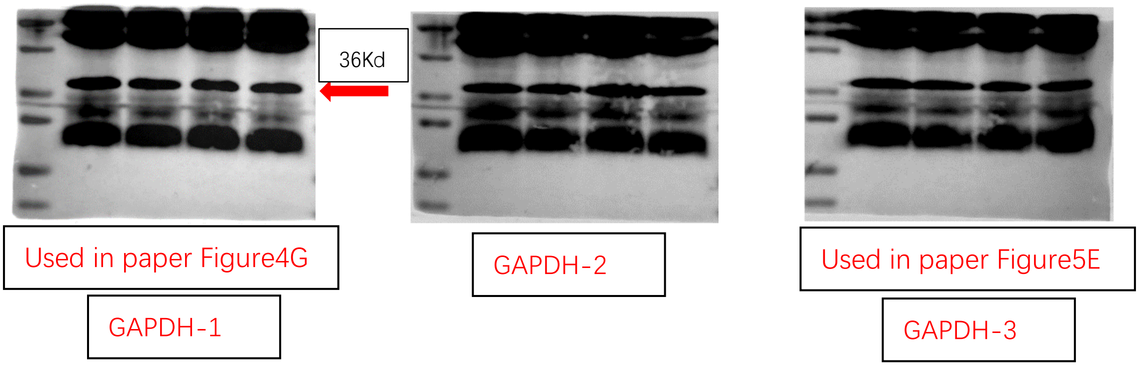

	GAPDH-1	GAPDH-2	GAPDH-3
lane 1	50429.0366	62965.48	71598.13
lane 2	50981.8148	50534.43	69030.69
lane 3	46497.6224	66019.76	51221.87
lane 4	53206.6934	44973.94	50667.76

Sirt1 (Figure 7A)

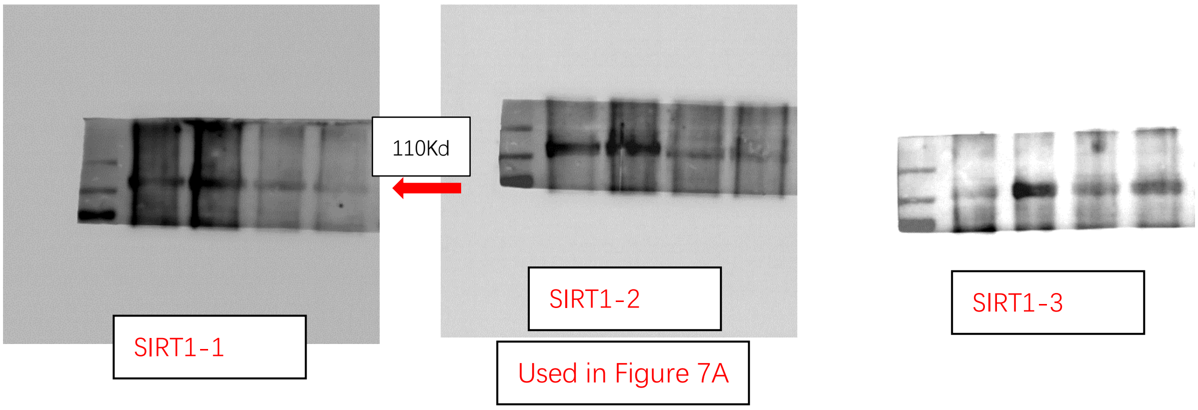

	SIRT1 -1	SIRT1 -2	SIRT1 -3
lane 1	36273.7939	30323.04	27908.33
lane 2	50341.9153	51870.23	44846.14
lane 3	19576.9949	30226.52	15518.84
lane 4	11844.1665	27487.81	21017.99

NLRP3 (Figure 7A)

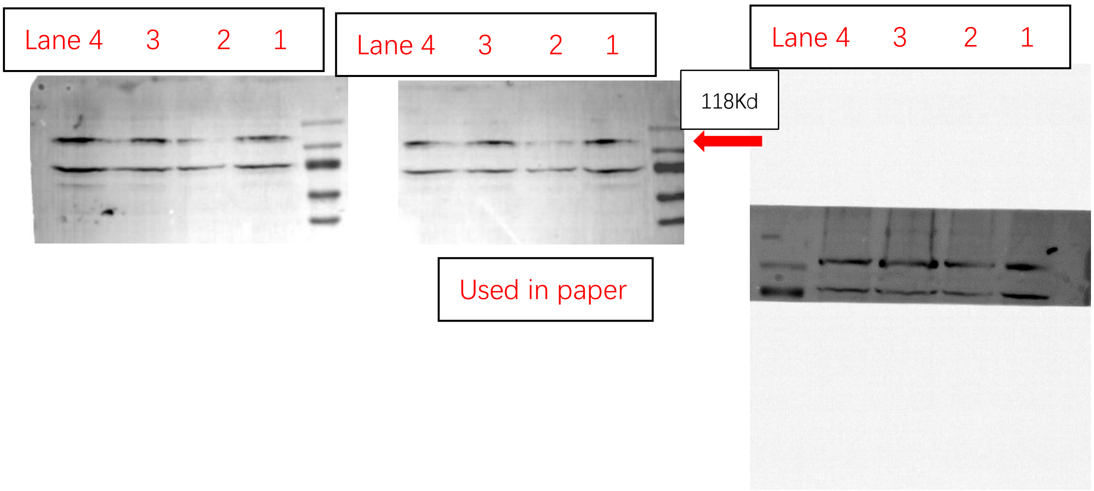

	NLRP3 2-1	NLRP3 2-2	NLRP3 2-3
lane 1	42148.99	35385.54	22118.1873
lane 2	9487.258	10207.84	16252.2082
lane 3	24427.11	30611.79	30984.7142
lane 4	22981.55	43245.86	25418.7437

IL-1β (Figure 7A)

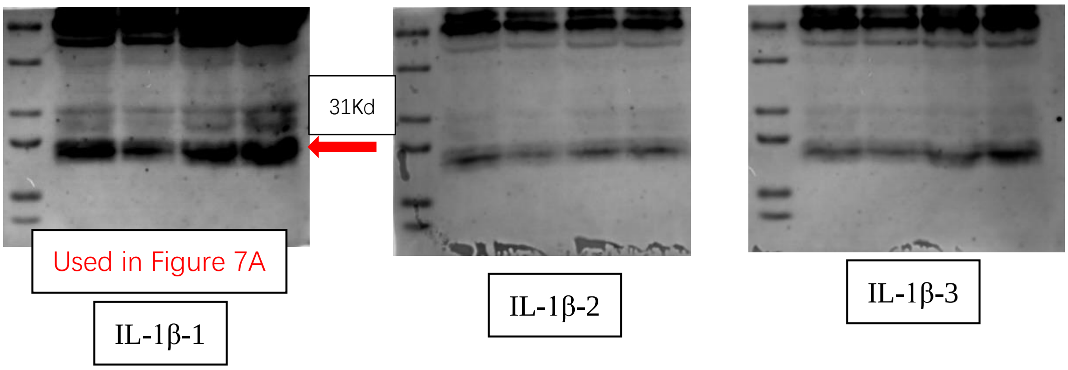

	IL-1β-1	IL-1β-2	IL-1β-3
lane 1	64484.59	47643.8356	57706.79
lane 2	32643.33	36835.5513	27432.5
lane 3	60154.19	52122.1787	29894.87
lane 4	63271.87	58210.865	39527.42

GAPDH (Figure 7A)

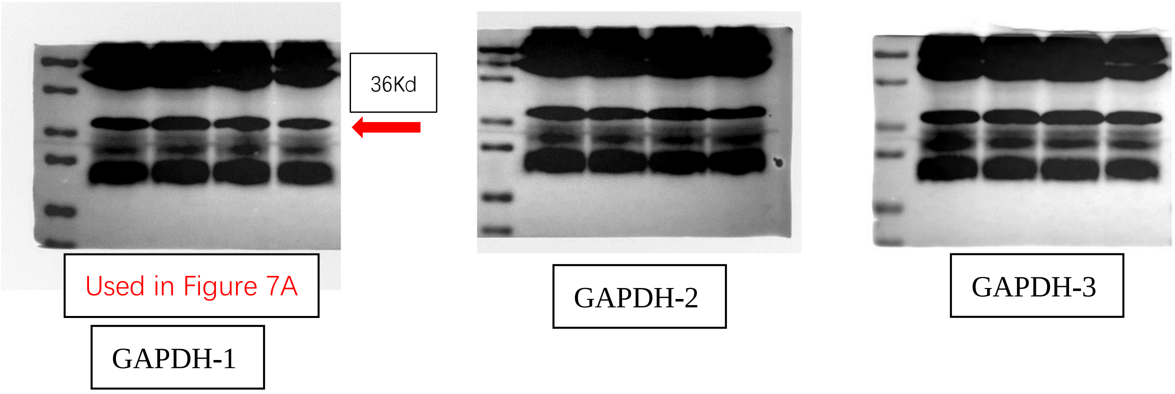

	GAPDH-1	GAPDH-2	GAPDH-3
lane 1	52856.1787	57192.37	49986.64
lane 2	54519.0366	63869.04	50714.21
lane 3	57053.5219	64276.64	61758.94
lane 4	52395.6934	56306.28	54584.67
