# Supplementary figures and images for: Forsythoside B alleviates cerebral ischemia-reperfusion injury via inhibiting NLRP3 inflammasome mediated by SIRT1 activation
Source: PLoS One. 2024 Jun 17;19(6):e0305541. doi: 10.1371/journal.pone.0305541 (PMC11182500; doi:10.1371/journal.pone.0305541)

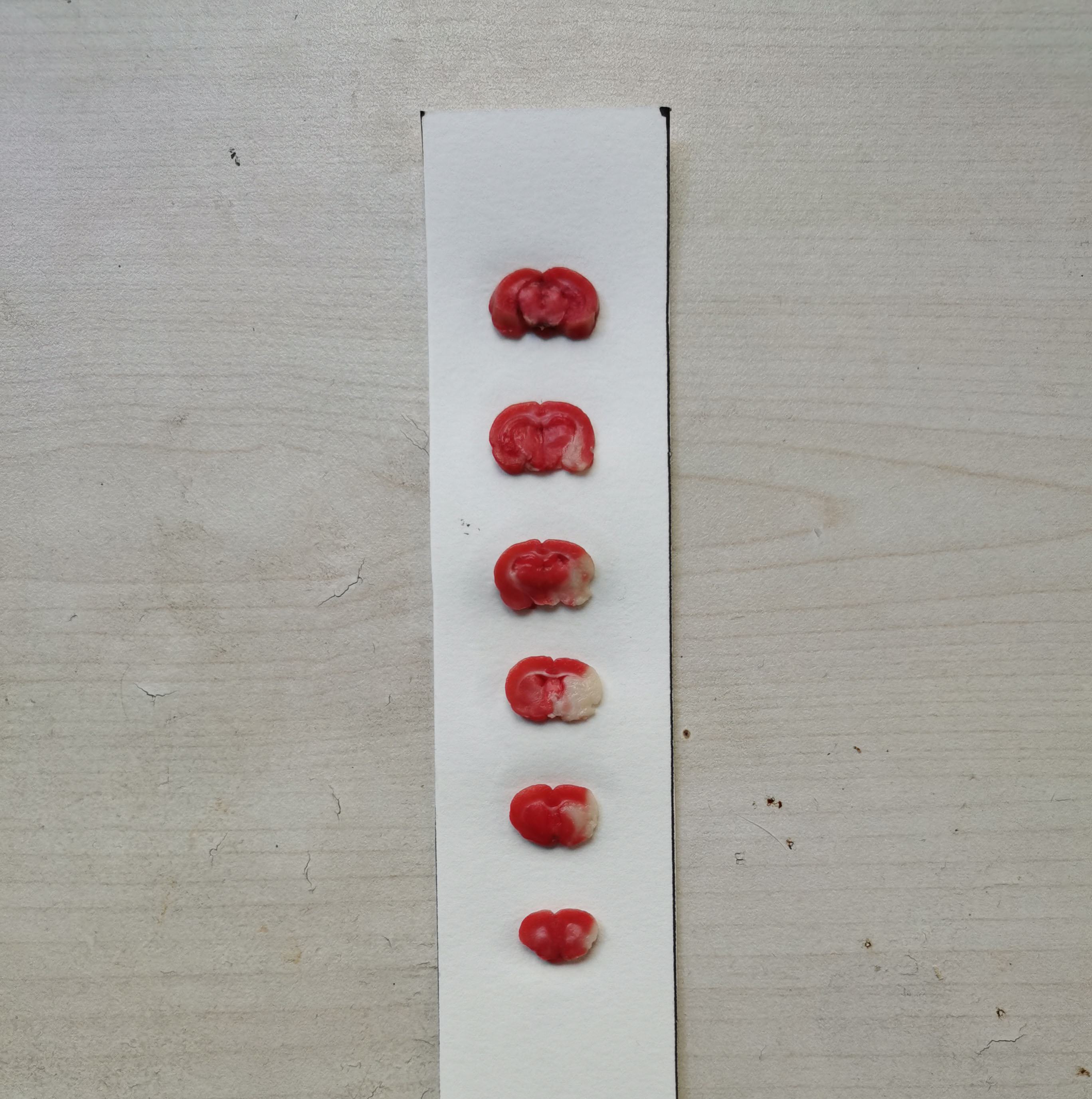

Supplement: S1 Raw data — (ZIP) [file pone.0305541.s002.zip › RAW DATA/FIG1/TTC/10.jpg]

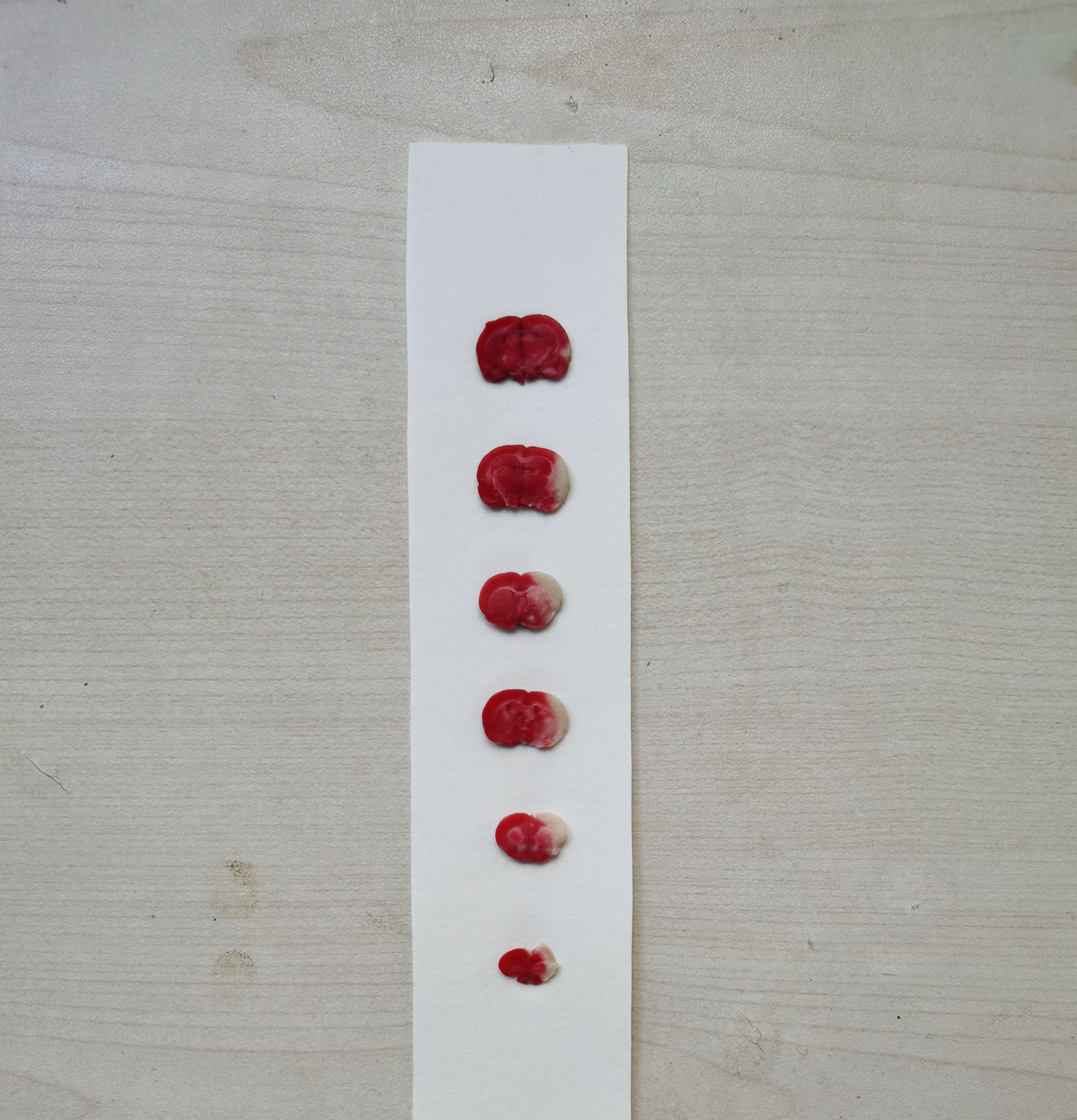

Supplement: S1 Raw data — (ZIP) [file pone.0305541.s002.zip › RAW DATA/FIG1/TTC/20.jpg]

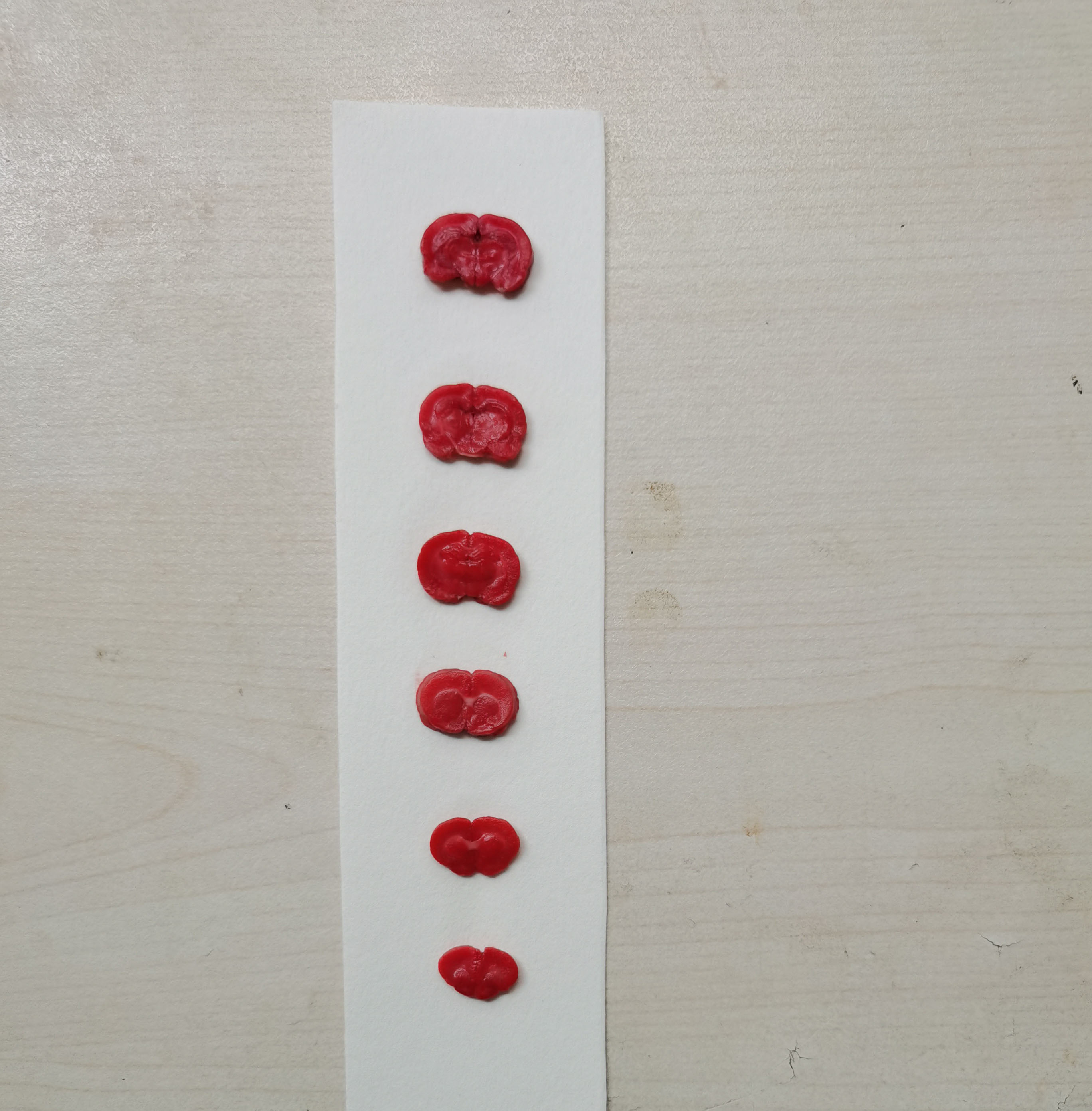

Supplement: S1 Raw data — (ZIP) [file pone.0305541.s002.zip › RAW DATA/FIG1/TTC/K1.jpg]

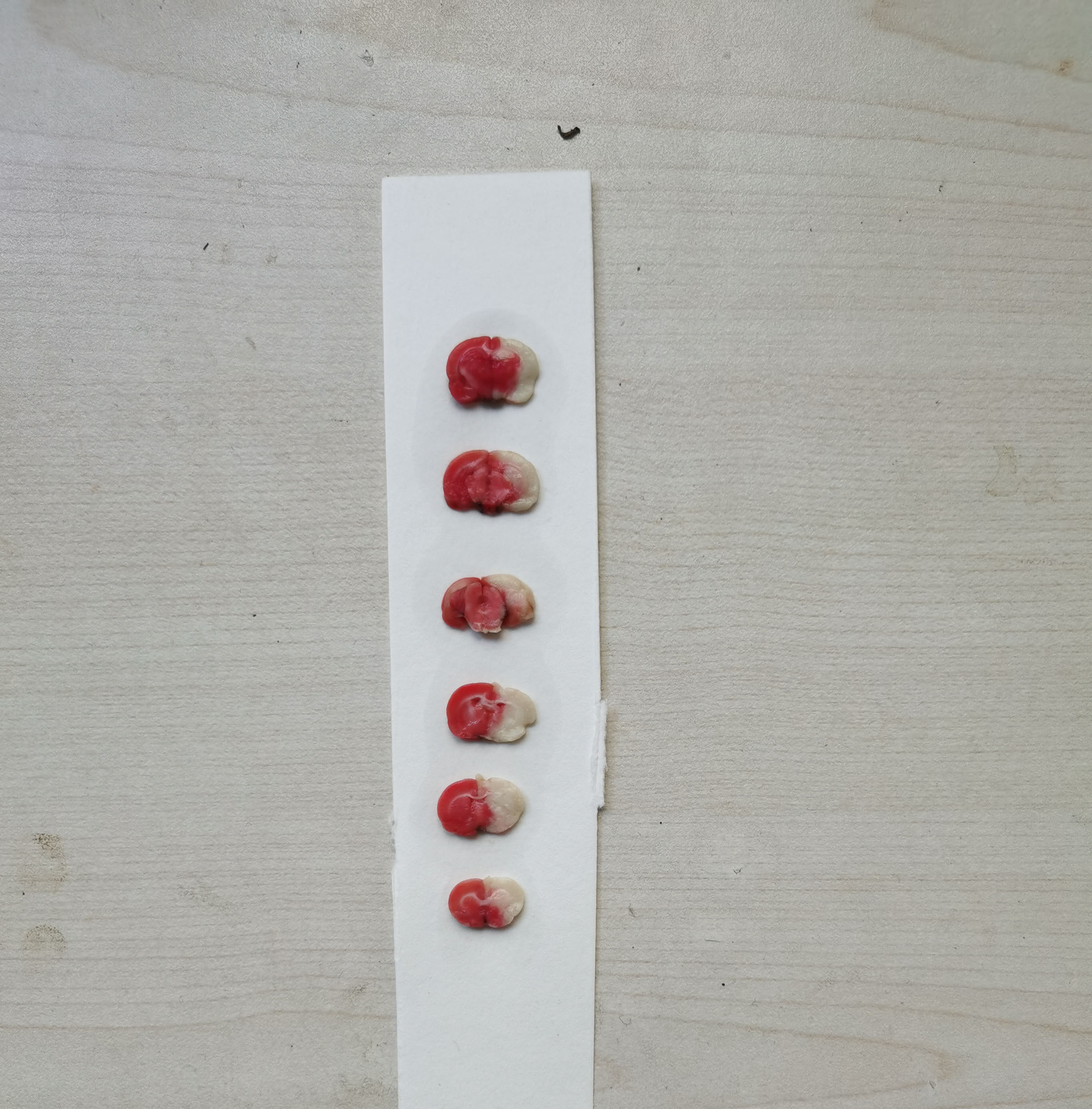

Supplement: S1 Raw data — (ZIP) [file pone.0305541.s002.zip › RAW DATA/FIG1/TTC/M.jpg]

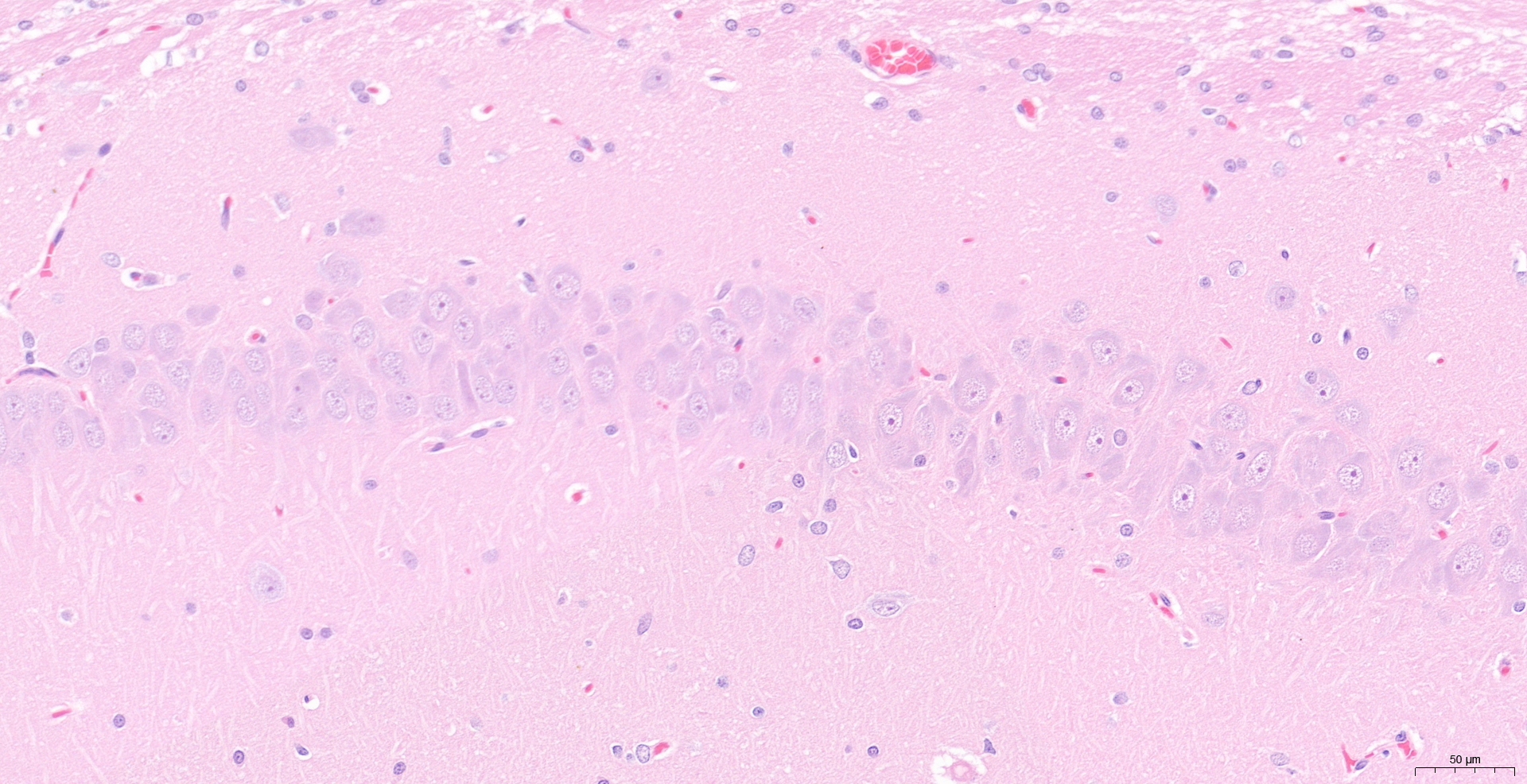

Supplement: S1 Raw data — (ZIP) [file pone.0305541.s002.zip › RAW DATA/FIG2/HE/10-CRC-HE_25.0x-1.jpg]

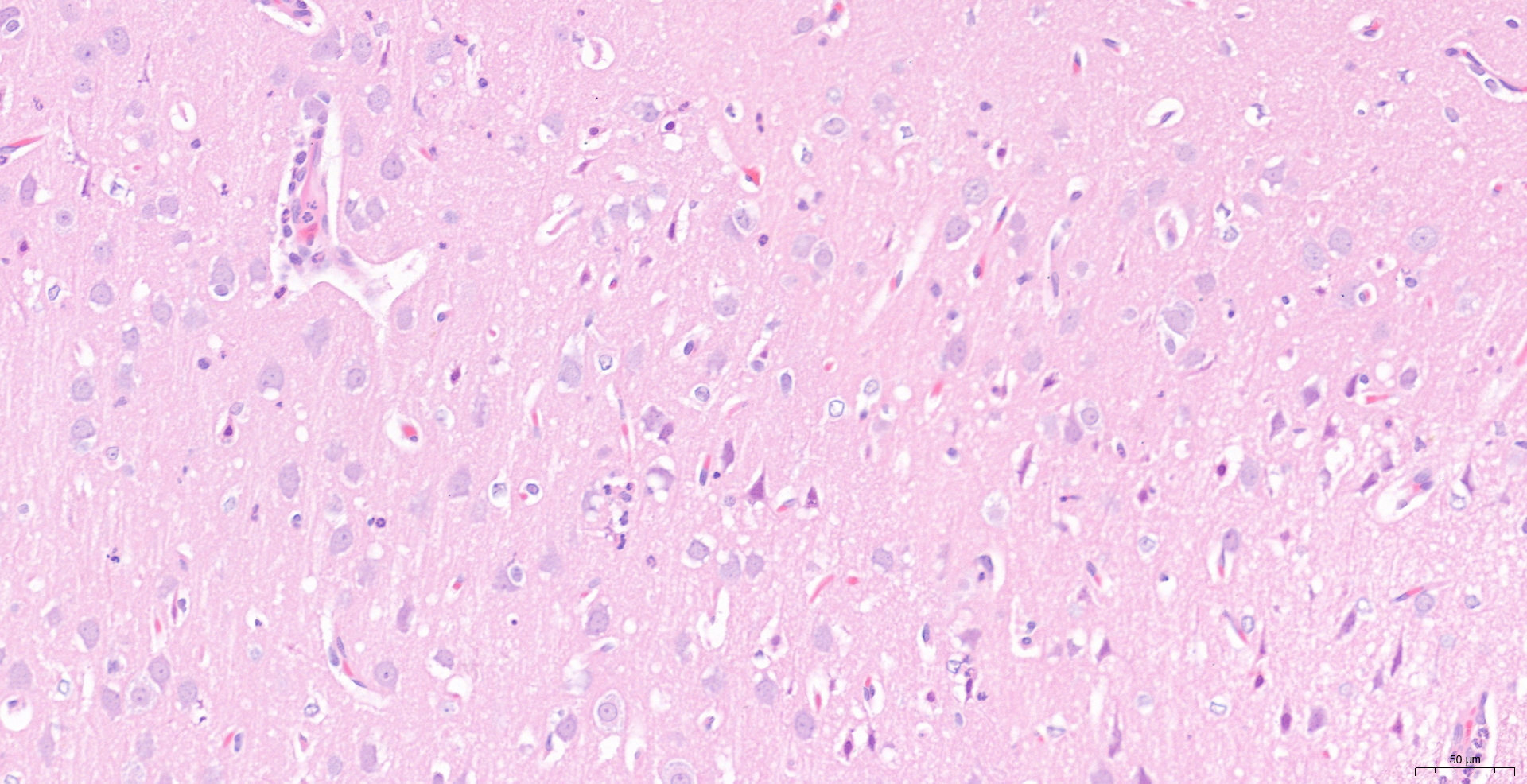

Supplement: S1 Raw data — (ZIP) [file pone.0305541.s002.zip › RAW DATA/FIG2/HE/10-CRC-HE_25.0x.jpg]

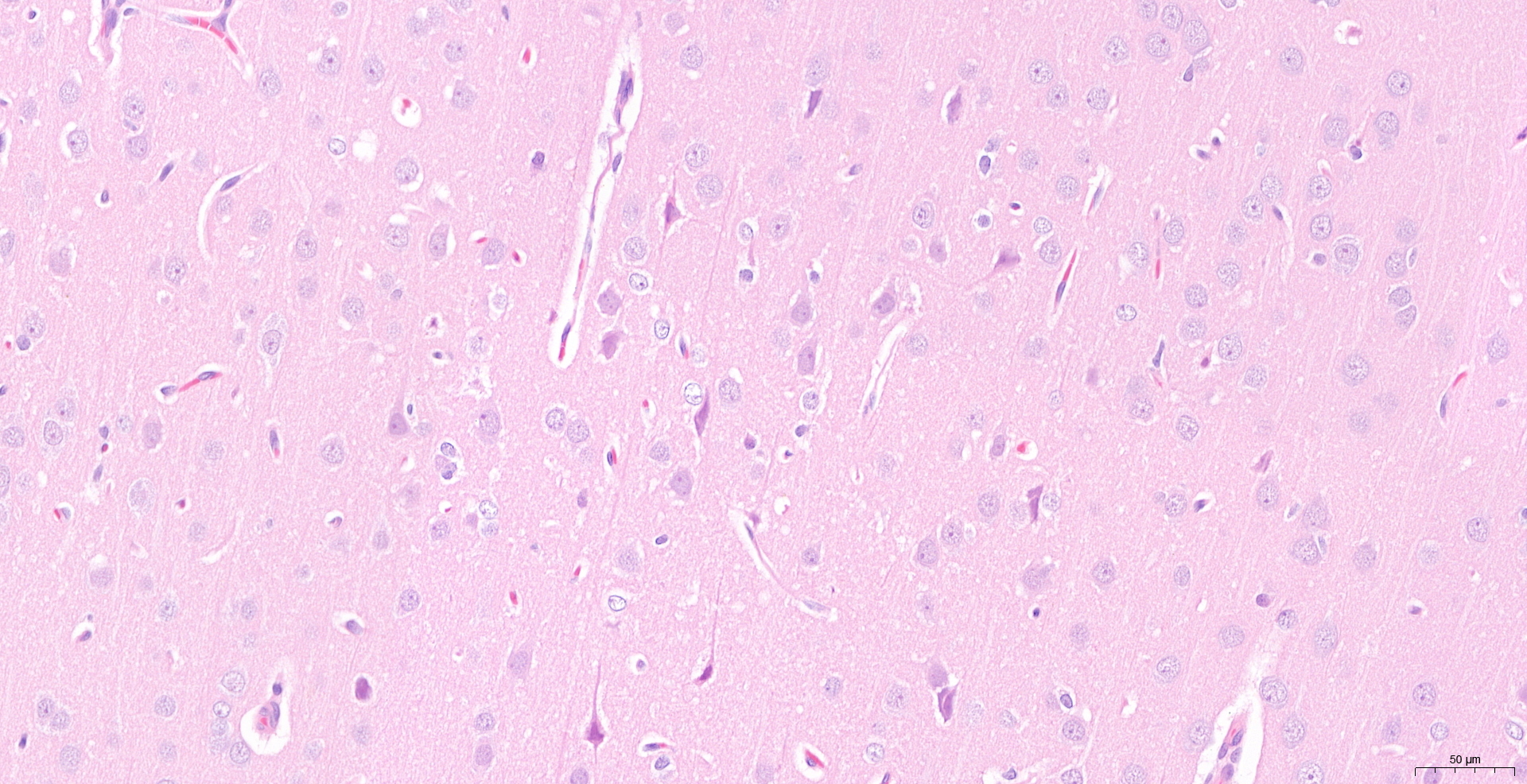

Supplement: S1 Raw data — (ZIP) [file pone.0305541.s002.zip › RAW DATA/FIG2/HE/20-CRC-HE_25.0x-1.jpg]

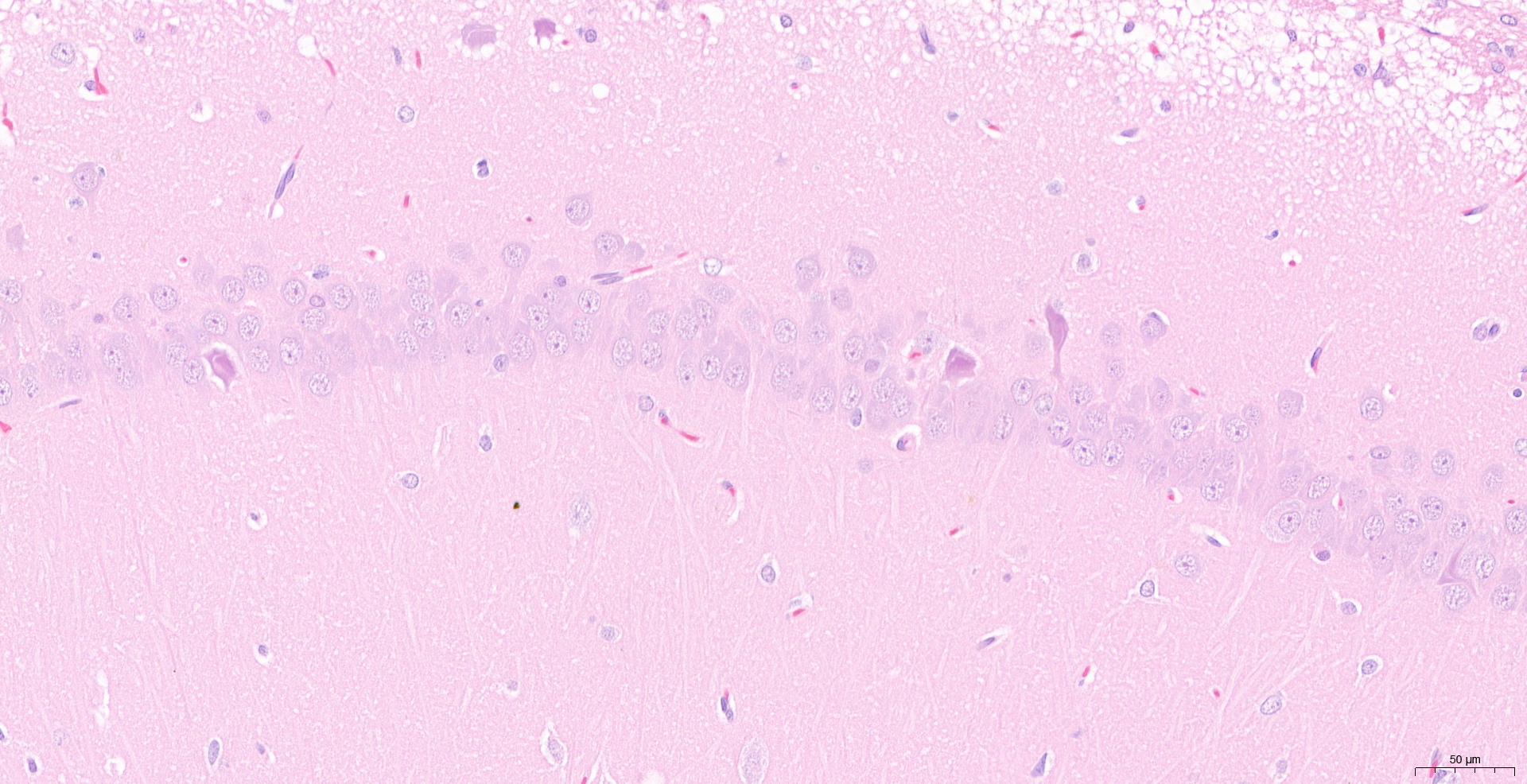

Supplement: S1 Raw data — (ZIP) [file pone.0305541.s002.zip › RAW DATA/FIG2/HE/20-CRC-HE_25.0x.jpg]

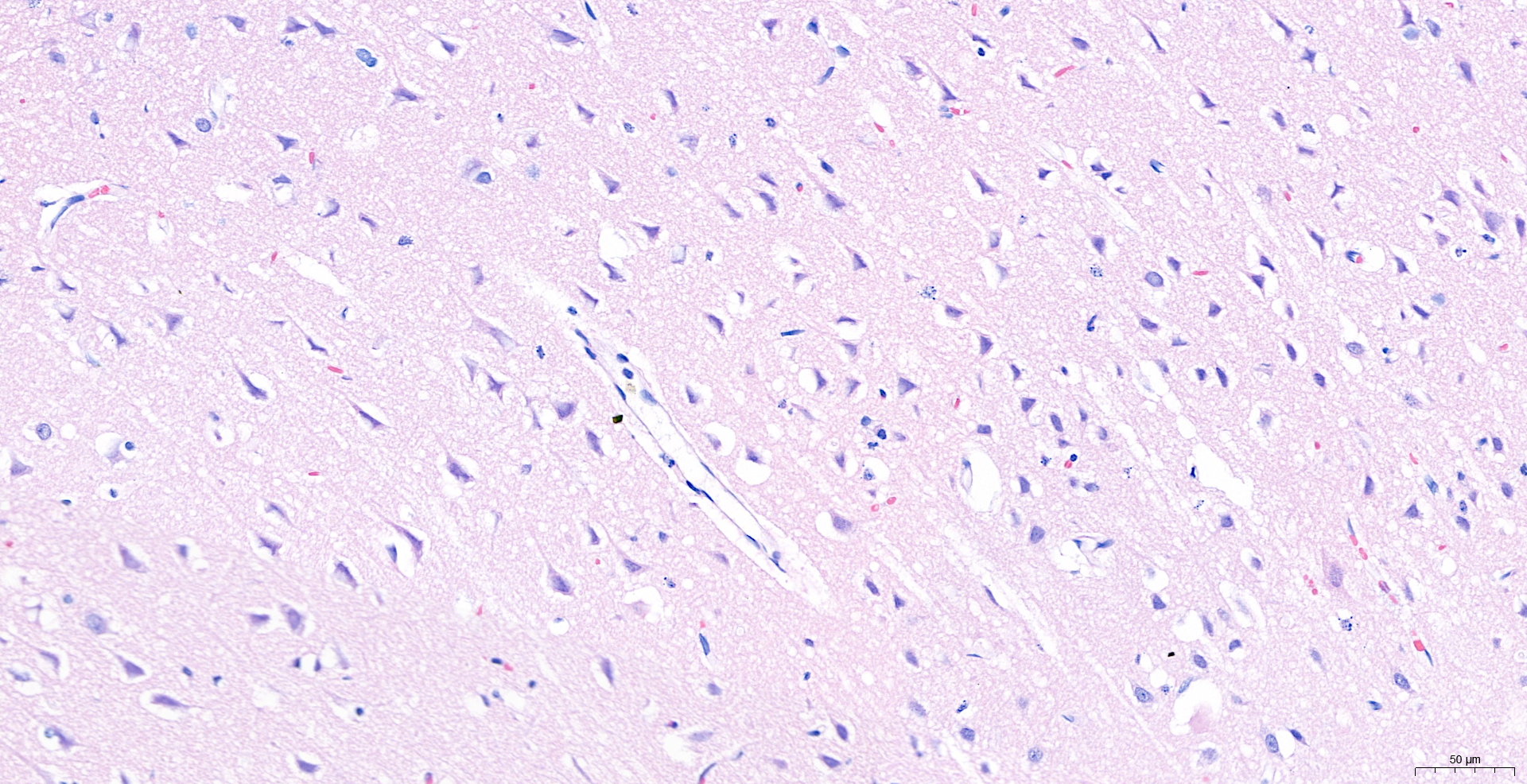

Supplement: S1 Raw data — (ZIP) [file pone.0305541.s002.zip › RAW DATA/FIG2/HE/M5-CRC-HE_25.0x-1.jpg]

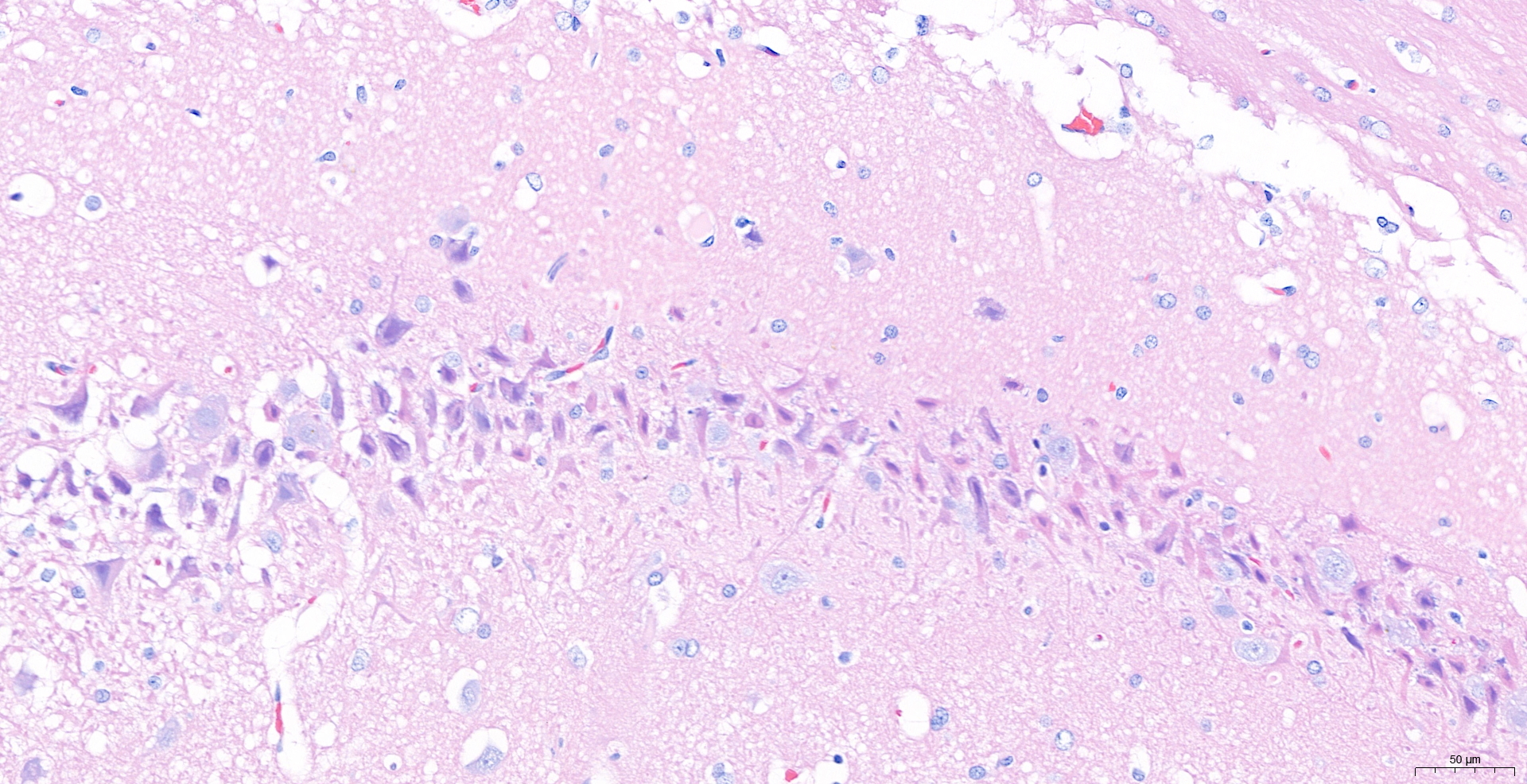

Supplement: S1 Raw data — (ZIP) [file pone.0305541.s002.zip › RAW DATA/FIG2/HE/M5-CRC-HE_25.0x.jpg]

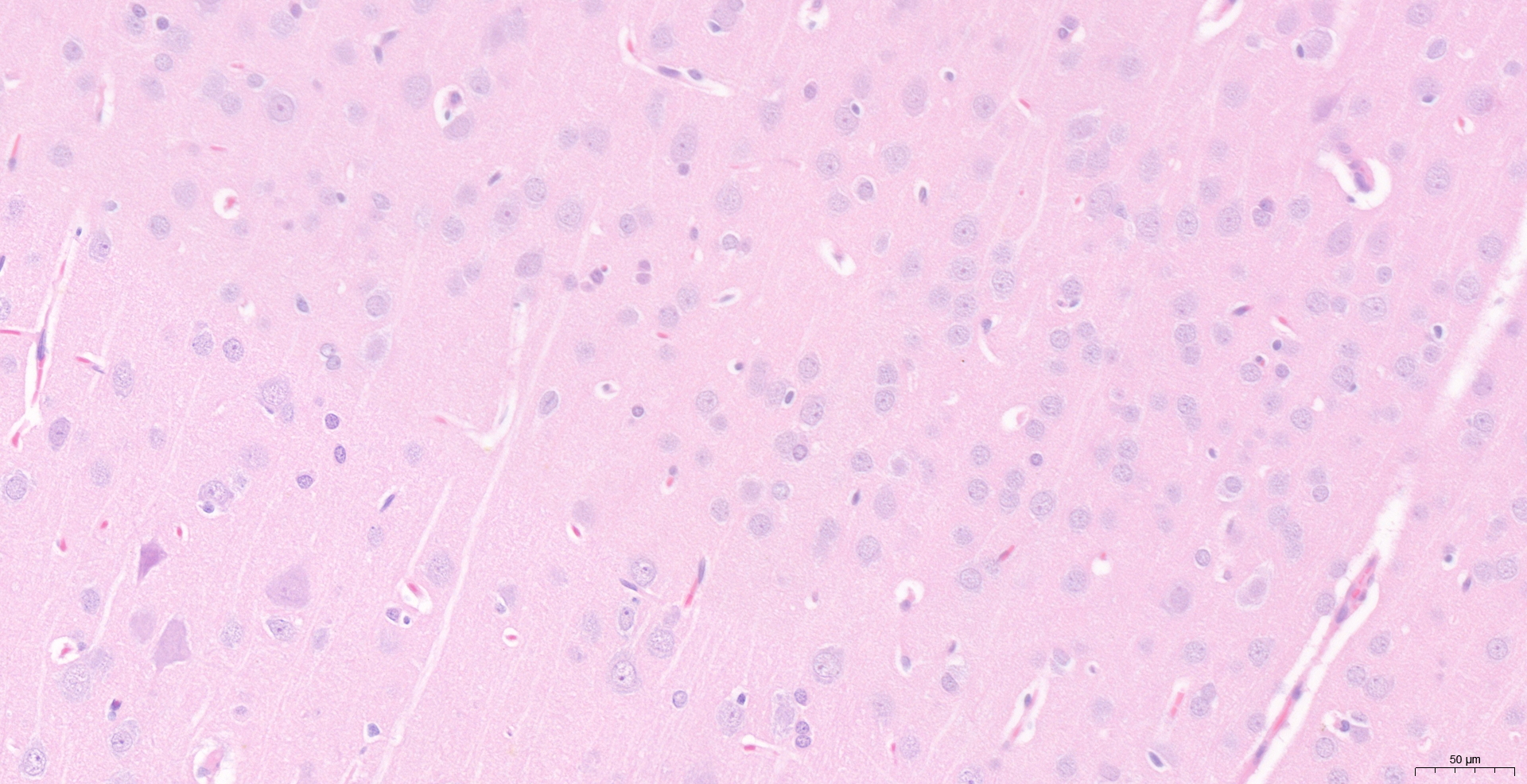

Supplement: S1 Raw data — (ZIP) [file pone.0305541.s002.zip › RAW DATA/FIG2/HE/┐╒░╫3-CRC-HE_25.0x-1.jpg]

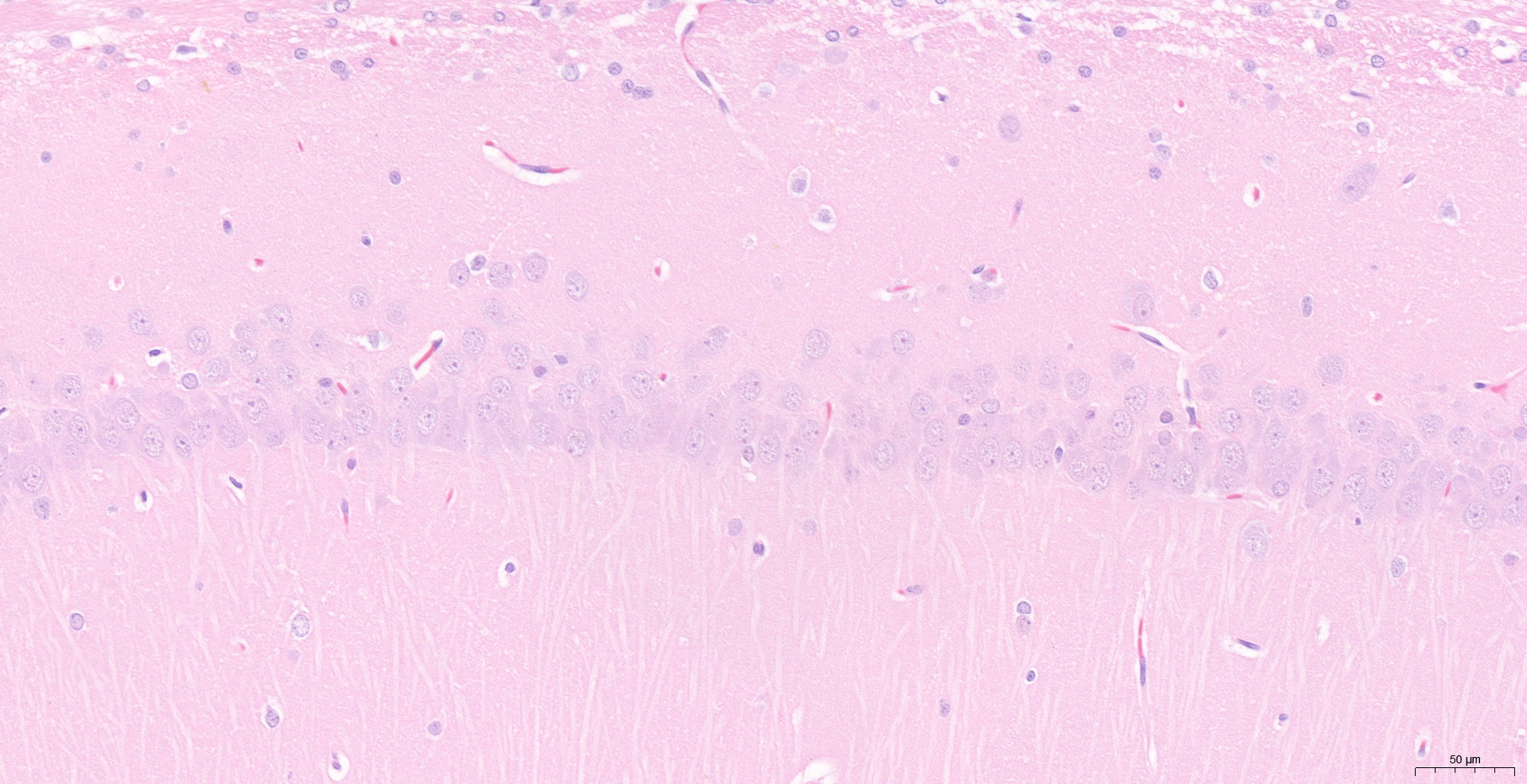

Supplement: S1 Raw data — (ZIP) [file pone.0305541.s002.zip › RAW DATA/FIG2/HE/┐╒░╫3-CRC-HE_25.0x.jpg]

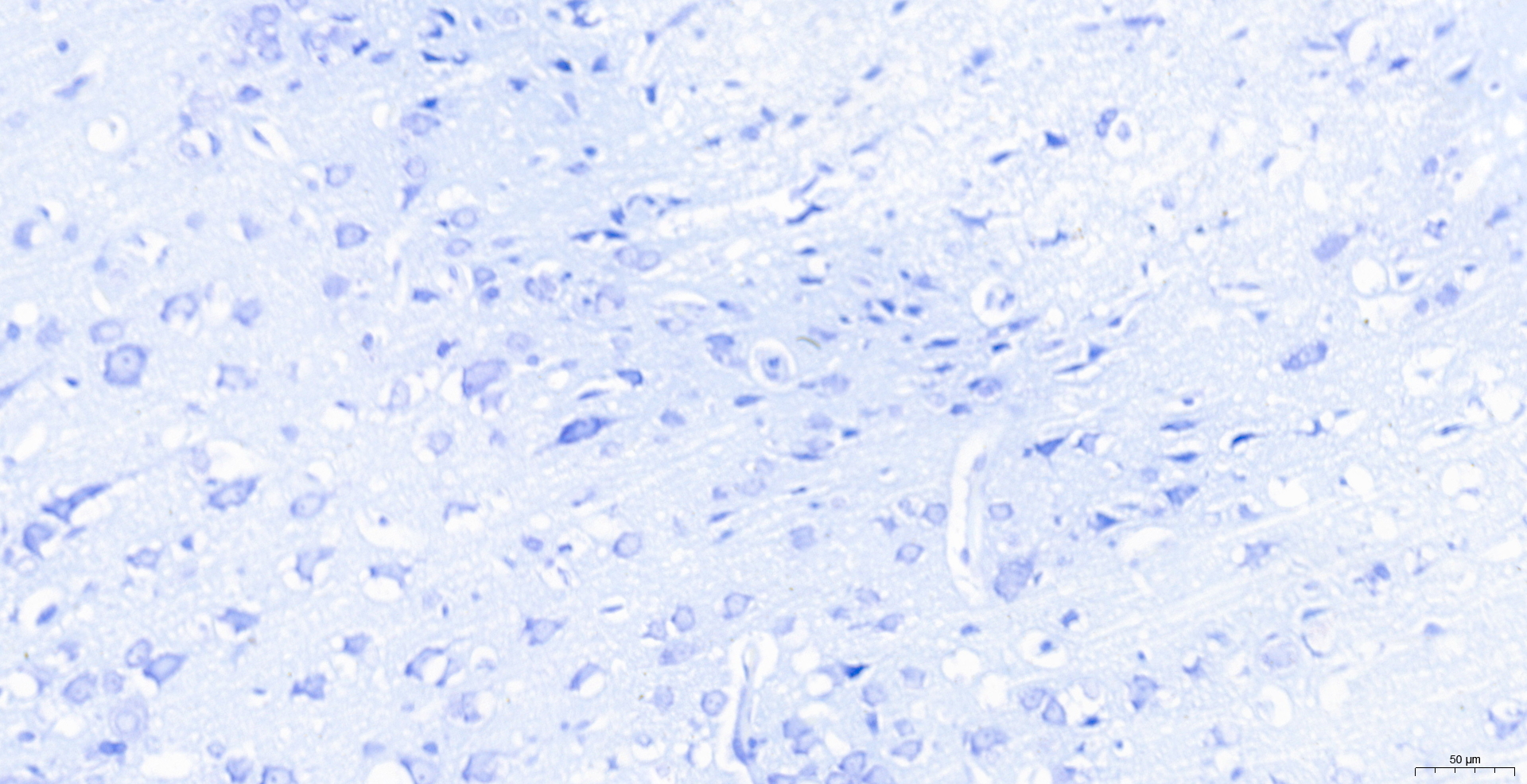

Supplement: S1 Raw data — (ZIP) [file pone.0305541.s002.zip › RAW DATA/FIG2/nissl/10-CRC-─ß╩╧_25.0x-1.jpg]

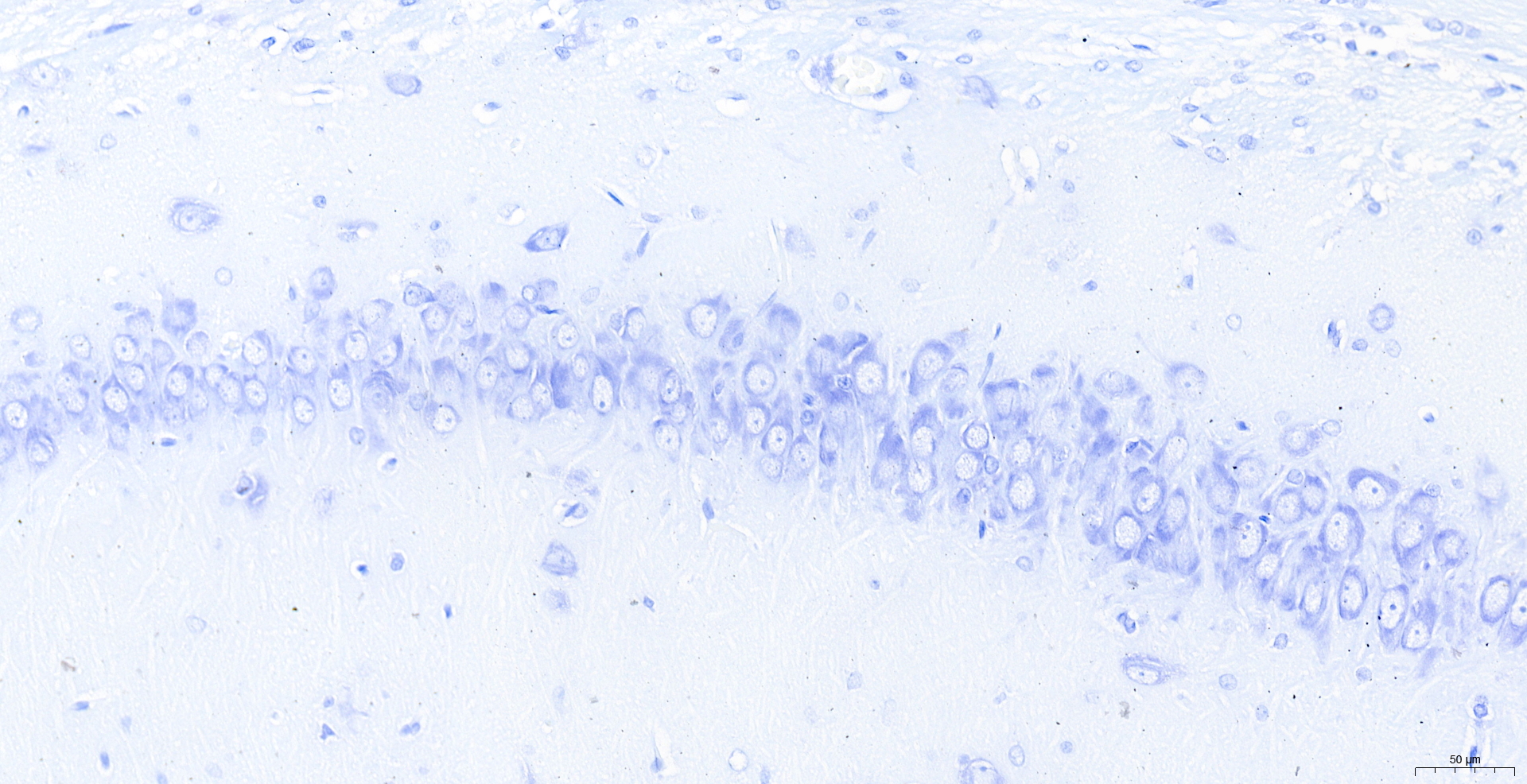

Supplement: S1 Raw data — (ZIP) [file pone.0305541.s002.zip › RAW DATA/FIG2/nissl/10-CRC-─ß╩╧_25.0x.jpg]

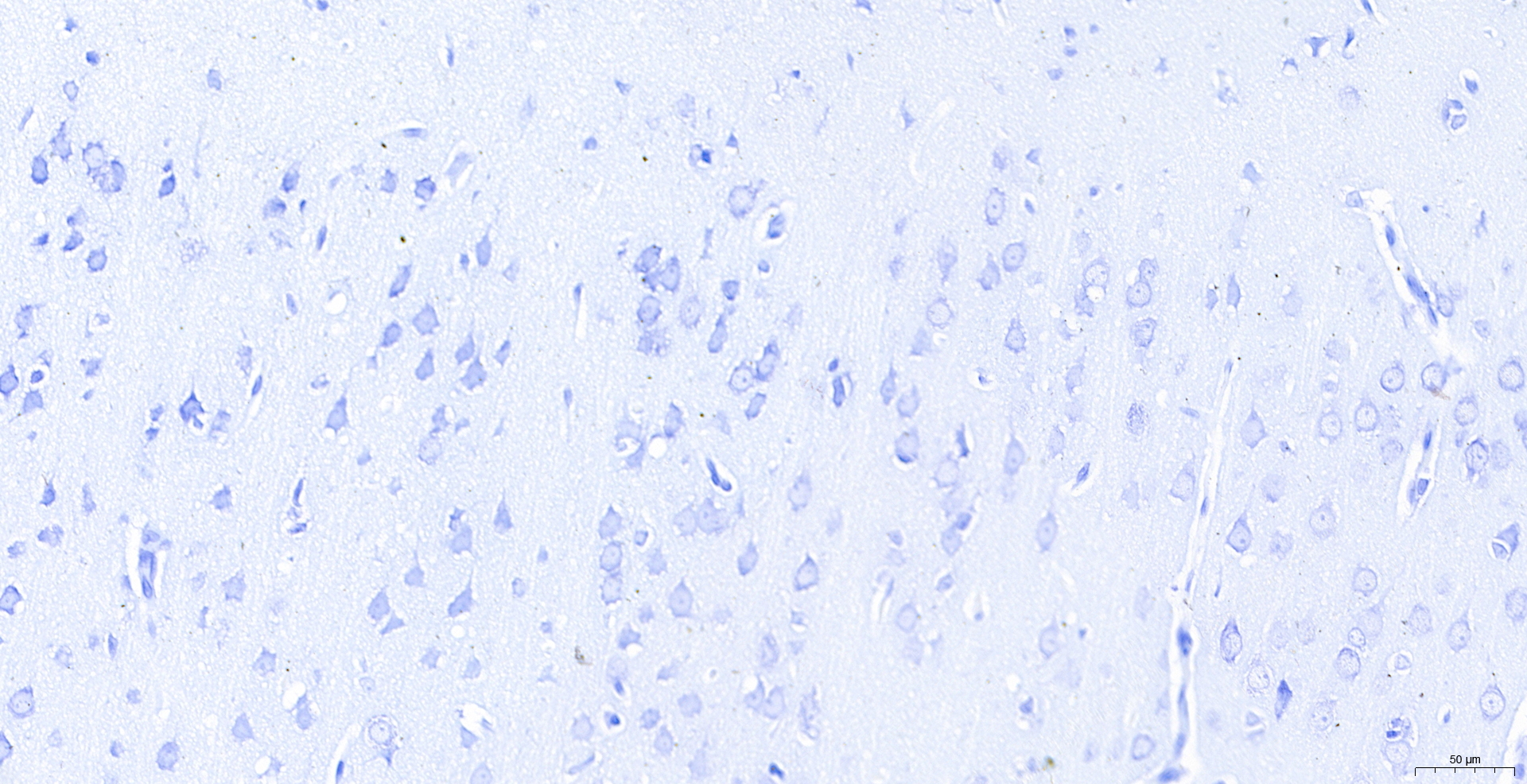

Supplement: S1 Raw data — (ZIP) [file pone.0305541.s002.zip › RAW DATA/FIG2/nissl/20-CRC-─ß╩╧_25.0x-1.jpg]

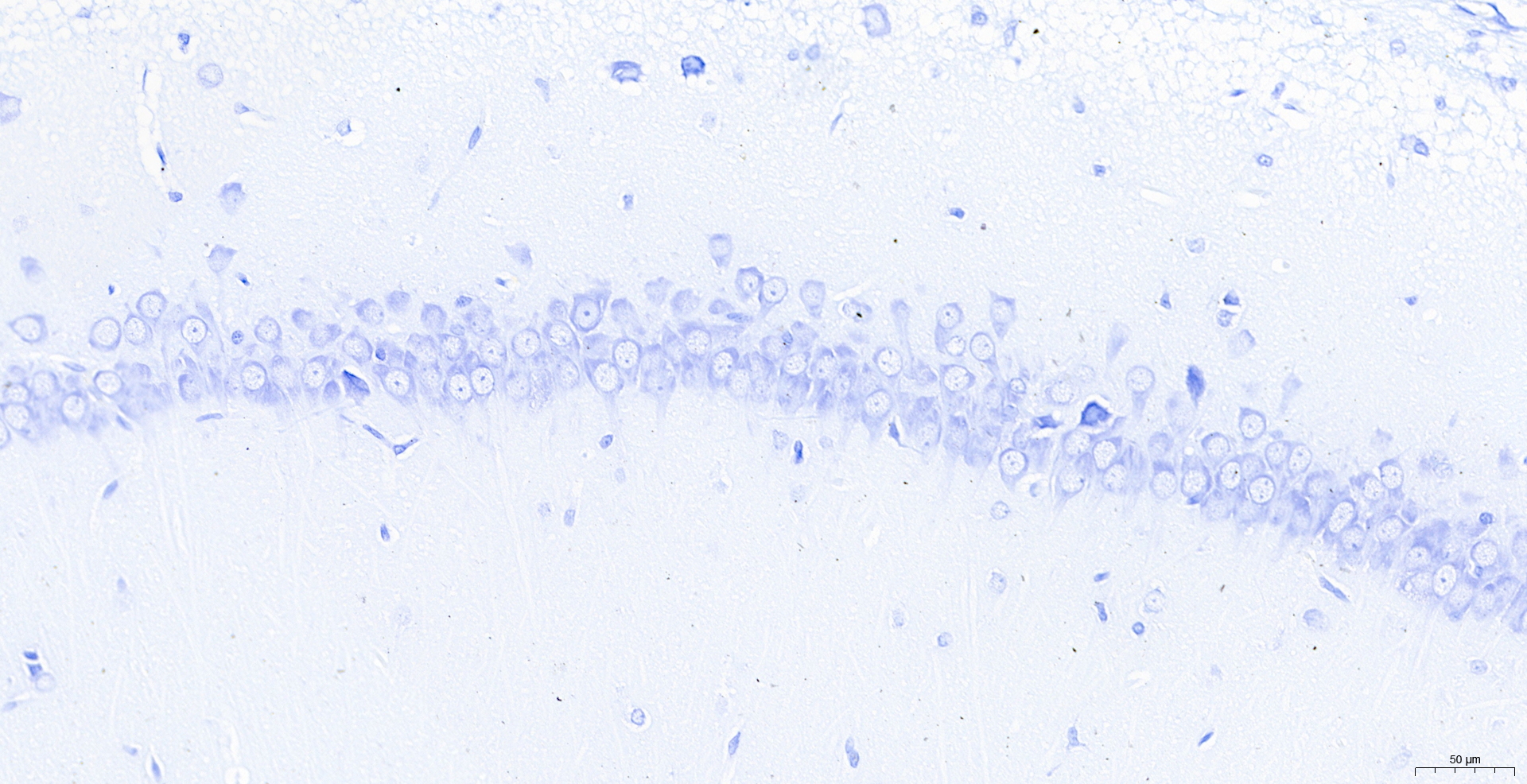

Supplement: S1 Raw data — (ZIP) [file pone.0305541.s002.zip › RAW DATA/FIG2/nissl/20-CRC-─ß╩╧_25.0x.jpg]

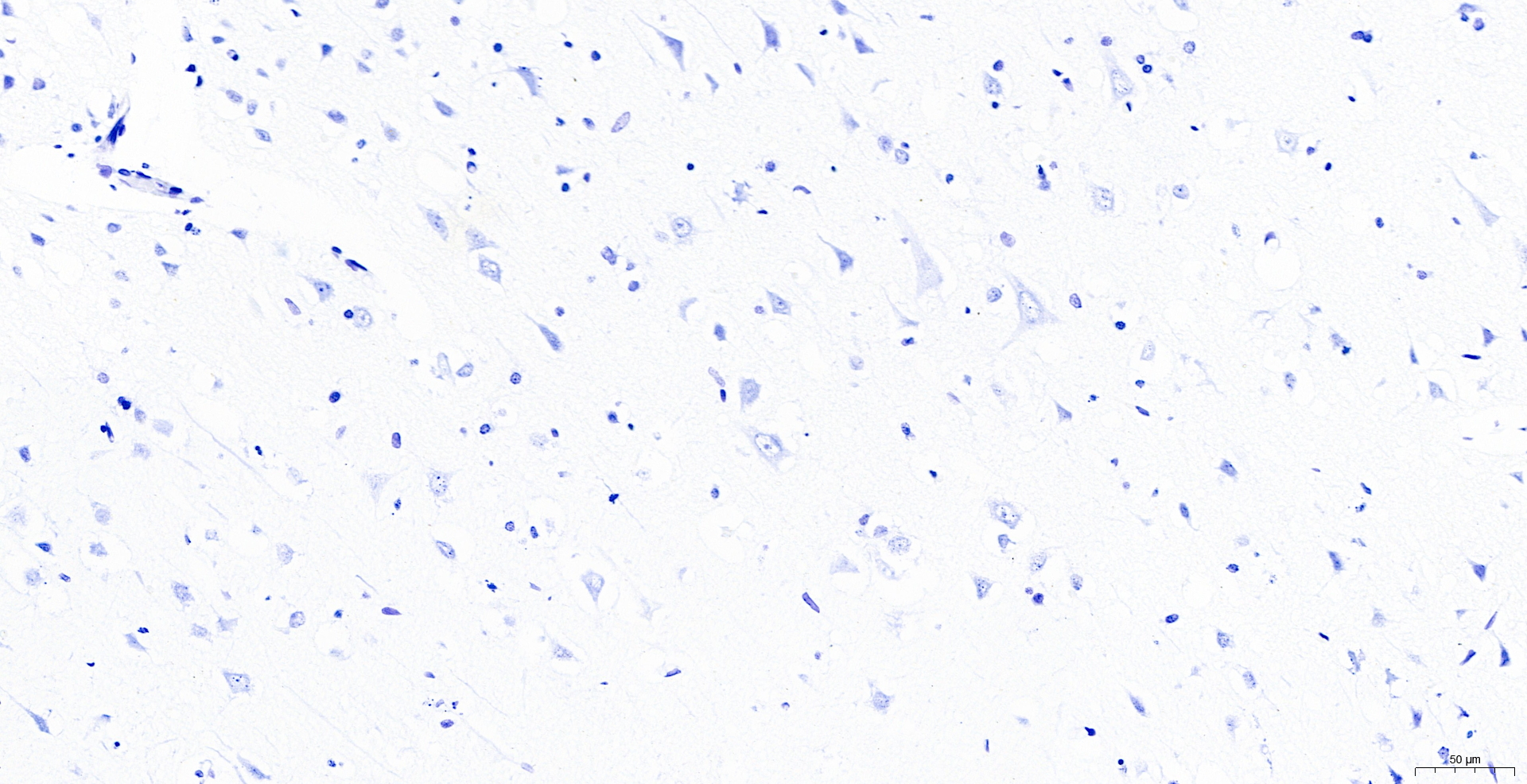

Supplement: S1 Raw data — (ZIP) [file pone.0305541.s002.zip › RAW DATA/FIG2/nissl/M5-CRC-─ß╩╧_25.0x-1.jpg]

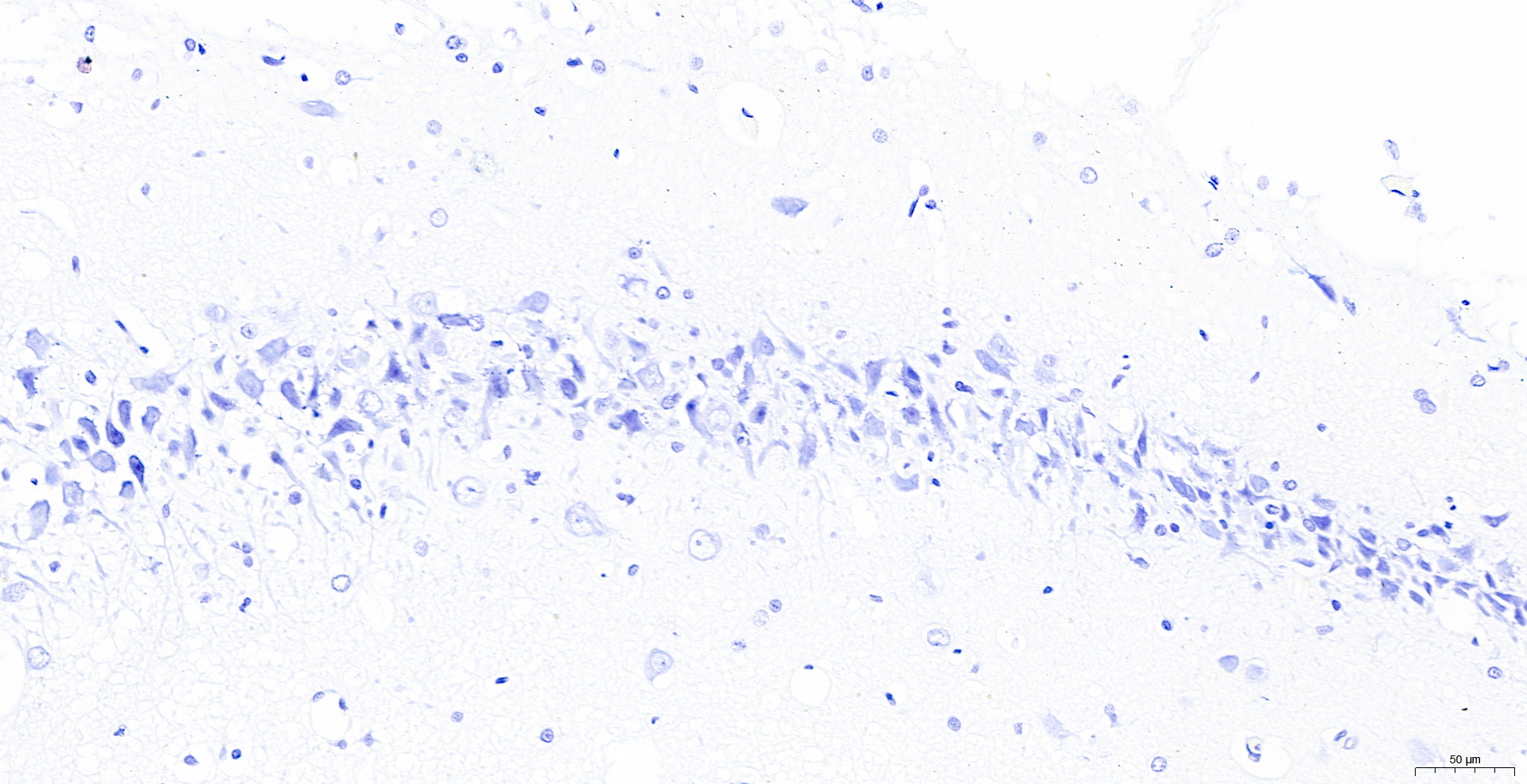

Supplement: S1 Raw data — (ZIP) [file pone.0305541.s002.zip › RAW DATA/FIG2/nissl/M5-CRC-─ß╩╧_25.0x.jpg]

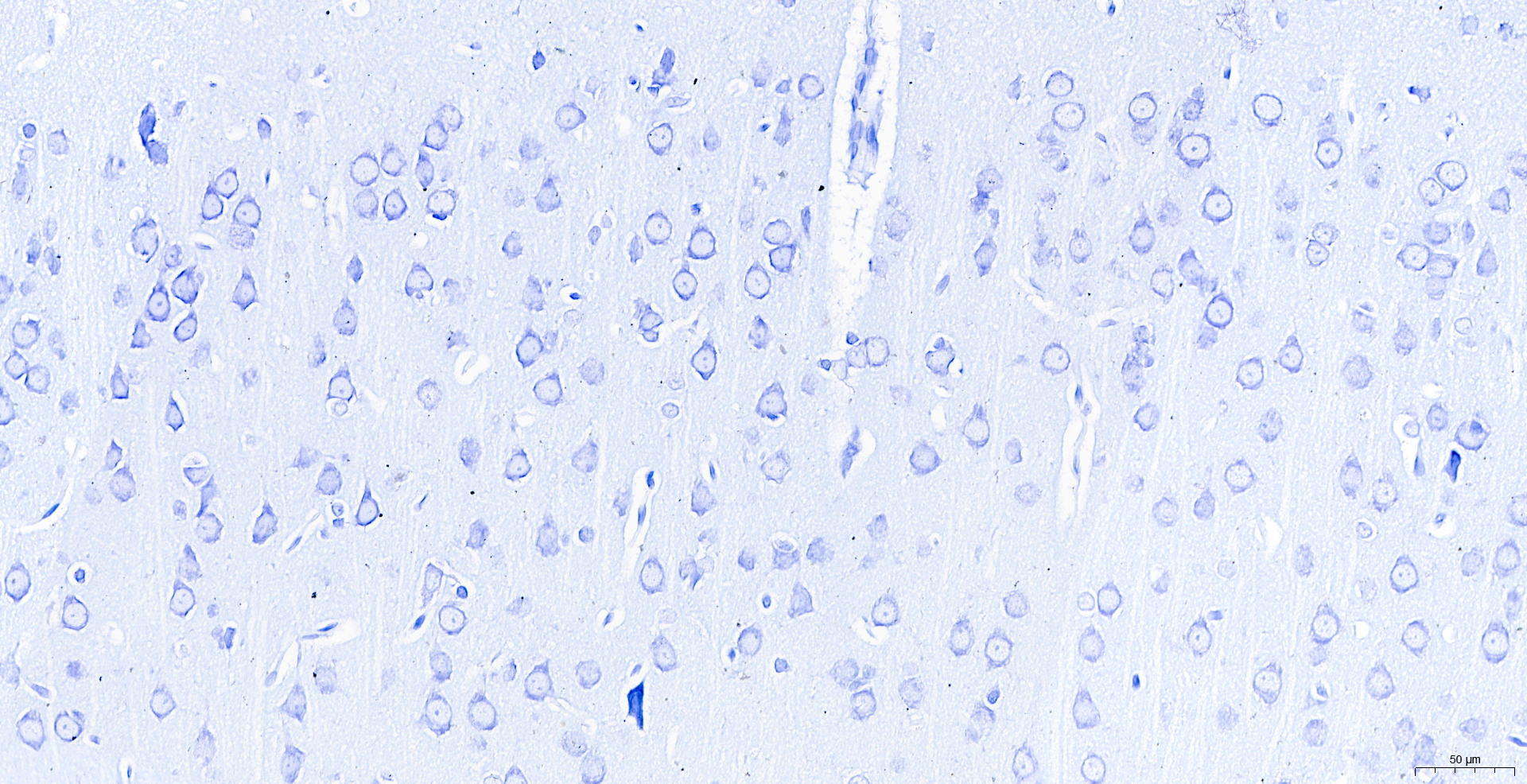

Supplement: S1 Raw data — (ZIP) [file pone.0305541.s002.zip › RAW DATA/FIG2/nissl/┐╒░╫3-CRC-─ß╩╧_25.0x-1.jpg]

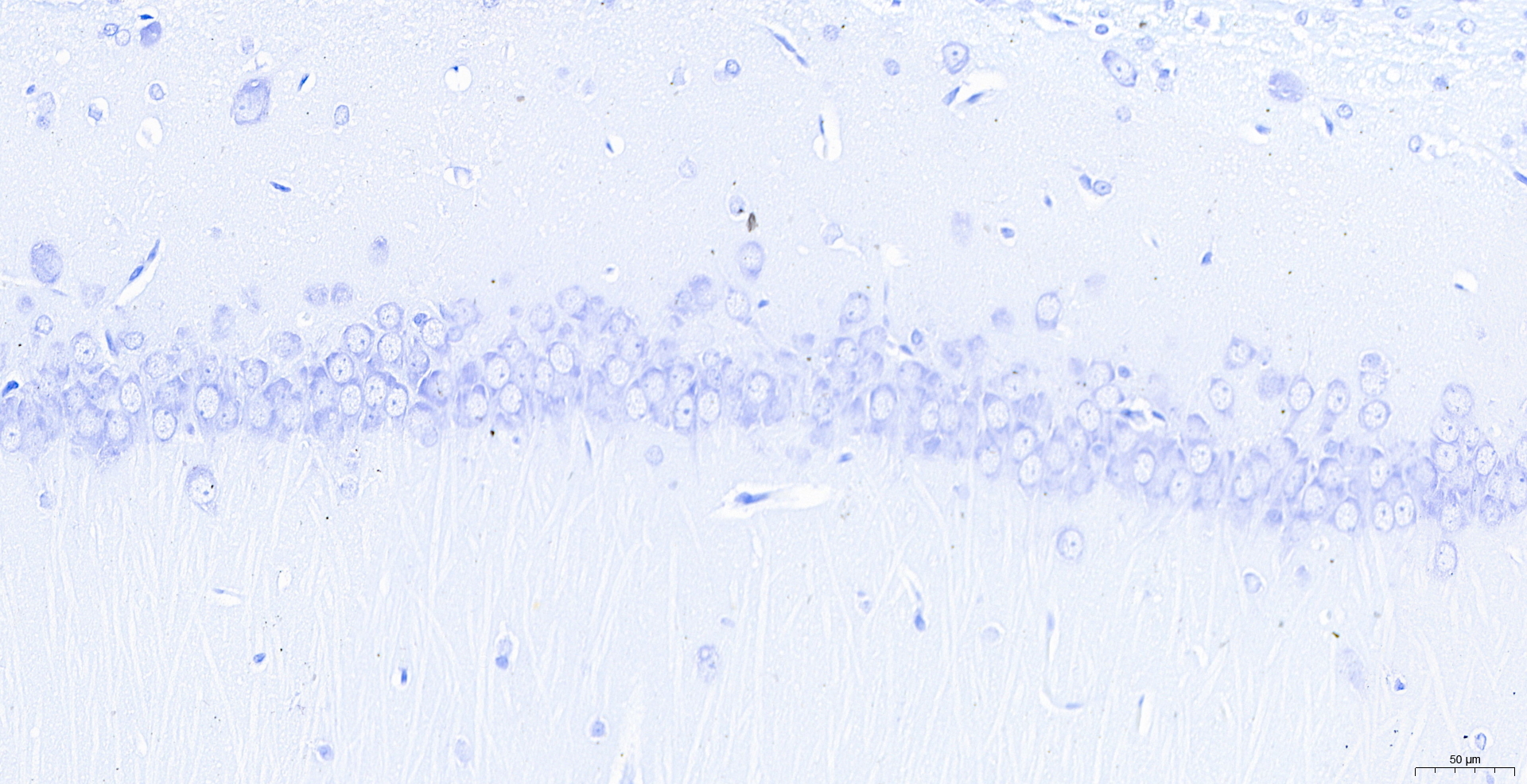

Supplement: S1 Raw data — (ZIP) [file pone.0305541.s002.zip › RAW DATA/FIG2/nissl/┐╒░╫3-CRC-─ß╩╧_25.0x.jpg]

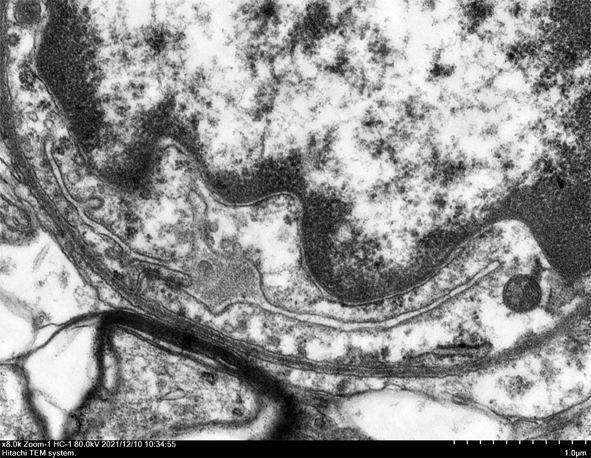

Supplement: S1 Raw data — (ZIP) [file pone.0305541.s002.zip › RAW DATA/FIG2/╡τ╛╡/1-Img4973.tif]

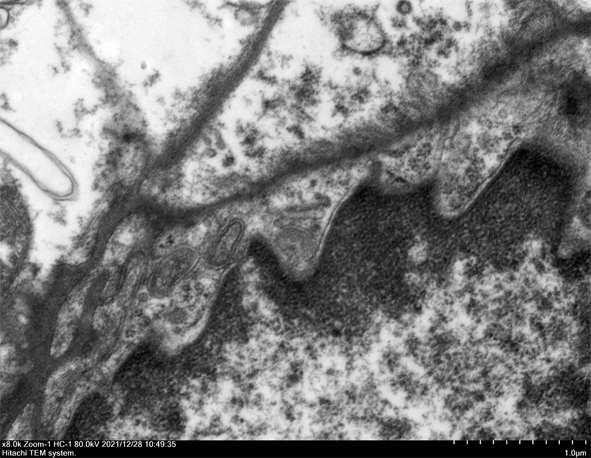

Supplement: S1 Raw data — (ZIP) [file pone.0305541.s002.zip › RAW DATA/FIG2/╡τ╛╡/2-Img3208-1.tif]

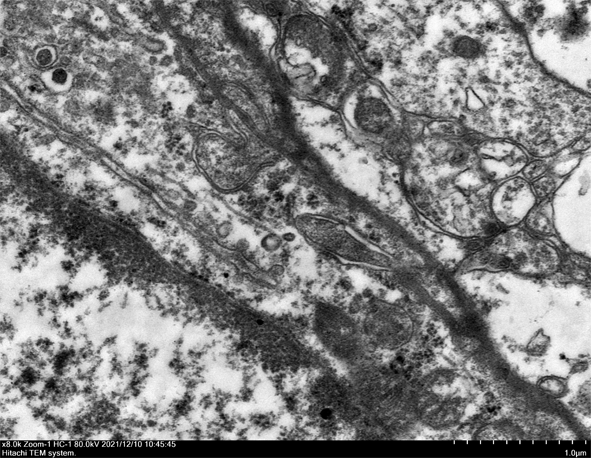

Supplement: S1 Raw data — (ZIP) [file pone.0305541.s002.zip › RAW DATA/FIG2/╡τ╛╡/5-Img4985.tif]

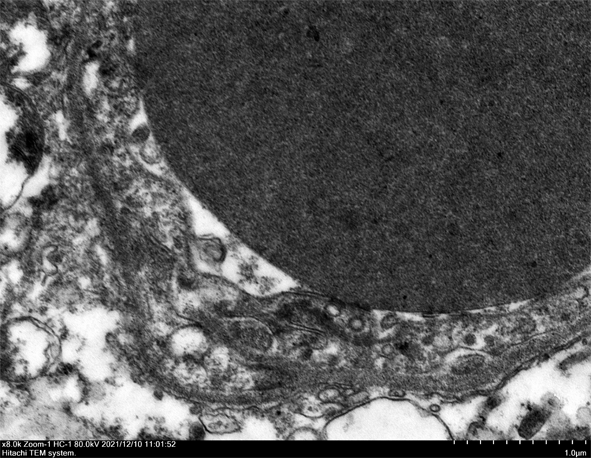

Supplement: S1 Raw data — (ZIP) [file pone.0305541.s002.zip › RAW DATA/FIG2/╡τ╛╡/8-Img5003.tif]

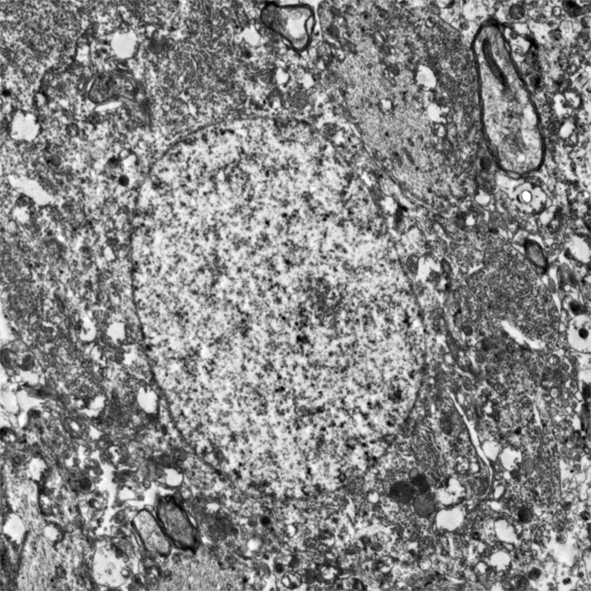

Supplement: S1 Raw data — (ZIP) [file pone.0305541.s002.zip › RAW DATA/FIG2/╡τ╛╡/hai-10-Img5049.tif]

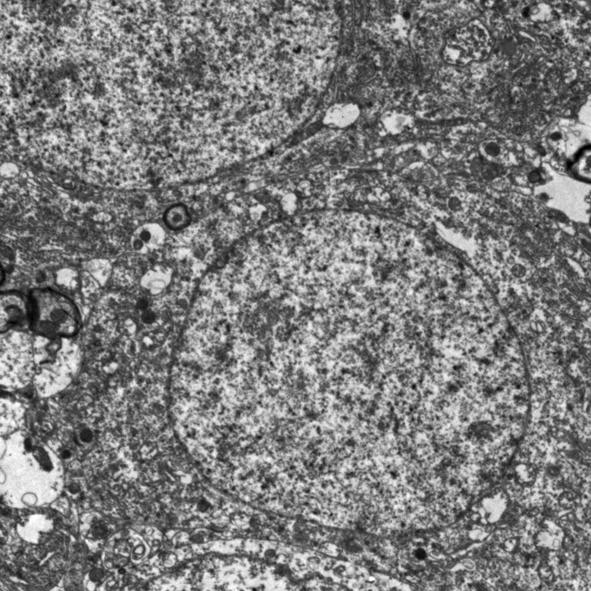

Supplement: S1 Raw data — (ZIP) [file pone.0305541.s002.zip › RAW DATA/FIG2/╡τ╛╡/hai-20-Img5018.tif]

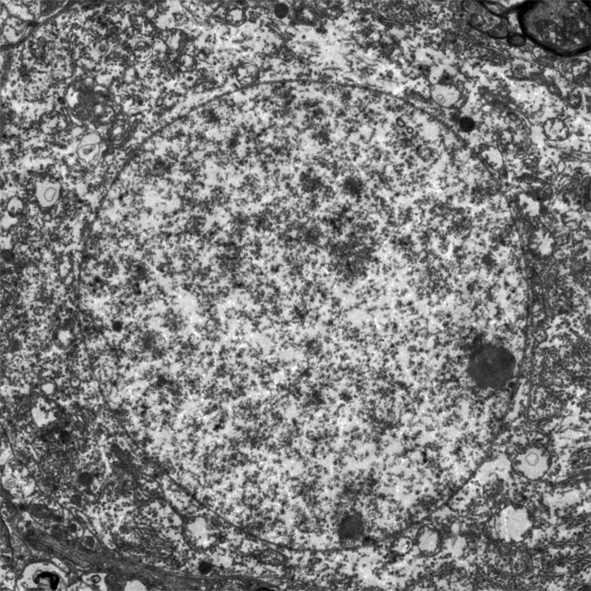

Supplement: S1 Raw data — (ZIP) [file pone.0305541.s002.zip › RAW DATA/FIG2/╡τ╛╡/hai-k-Img5009.tif]

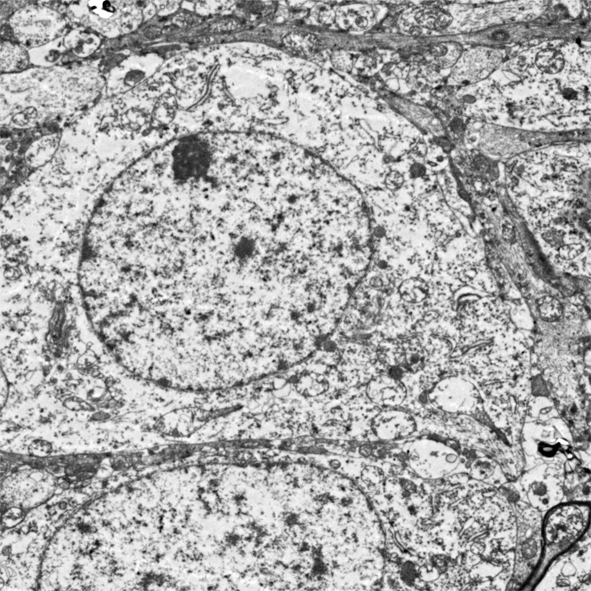

Supplement: S1 Raw data — (ZIP) [file pone.0305541.s002.zip › RAW DATA/FIG2/╡τ╛╡/hai-m-Img5031.tif]

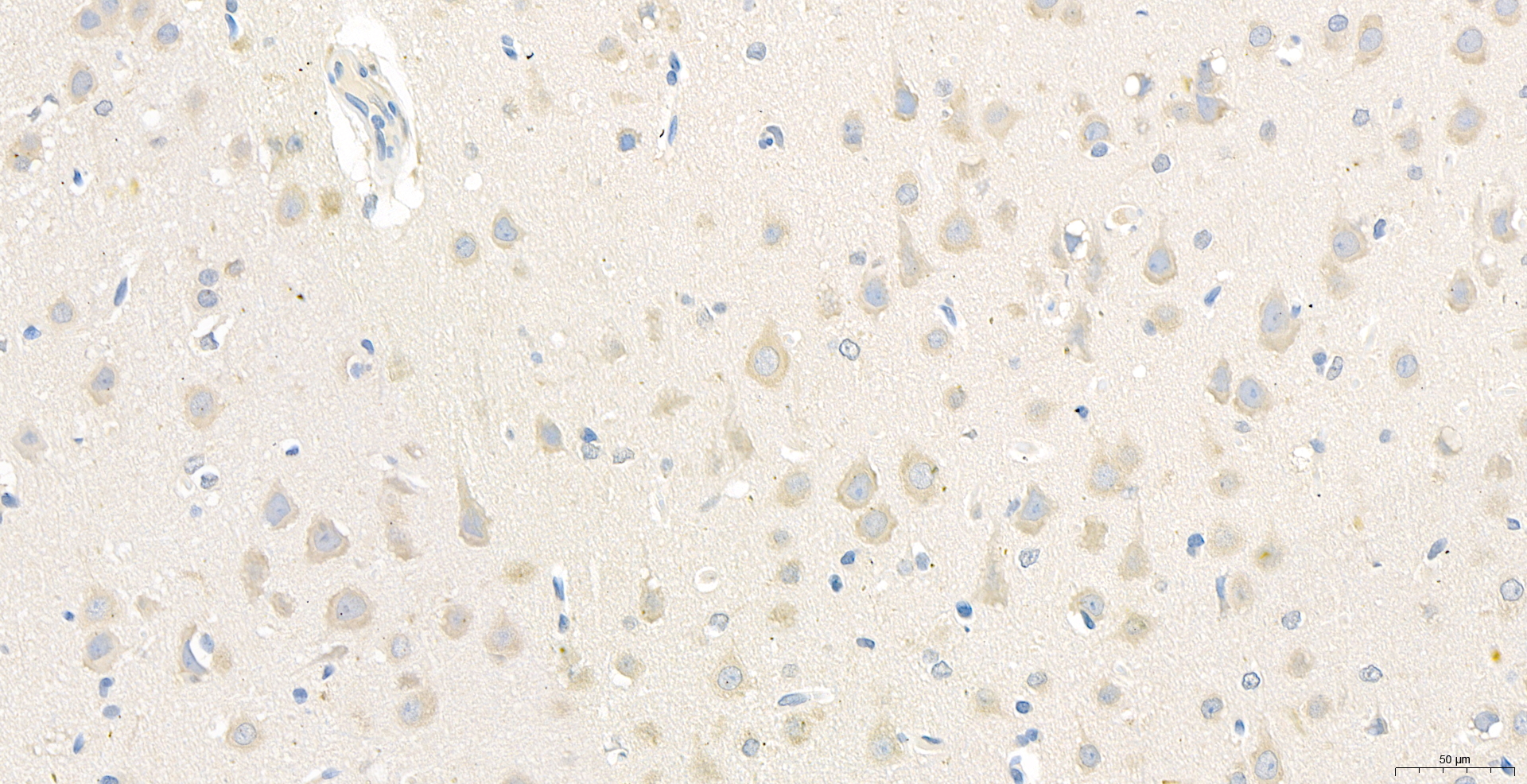

Supplement: S1 Raw data — (ZIP) [file pone.0305541.s002.zip › RAW DATA/FIG3/CAS3/10-100x_30.0x-HM.jpg]

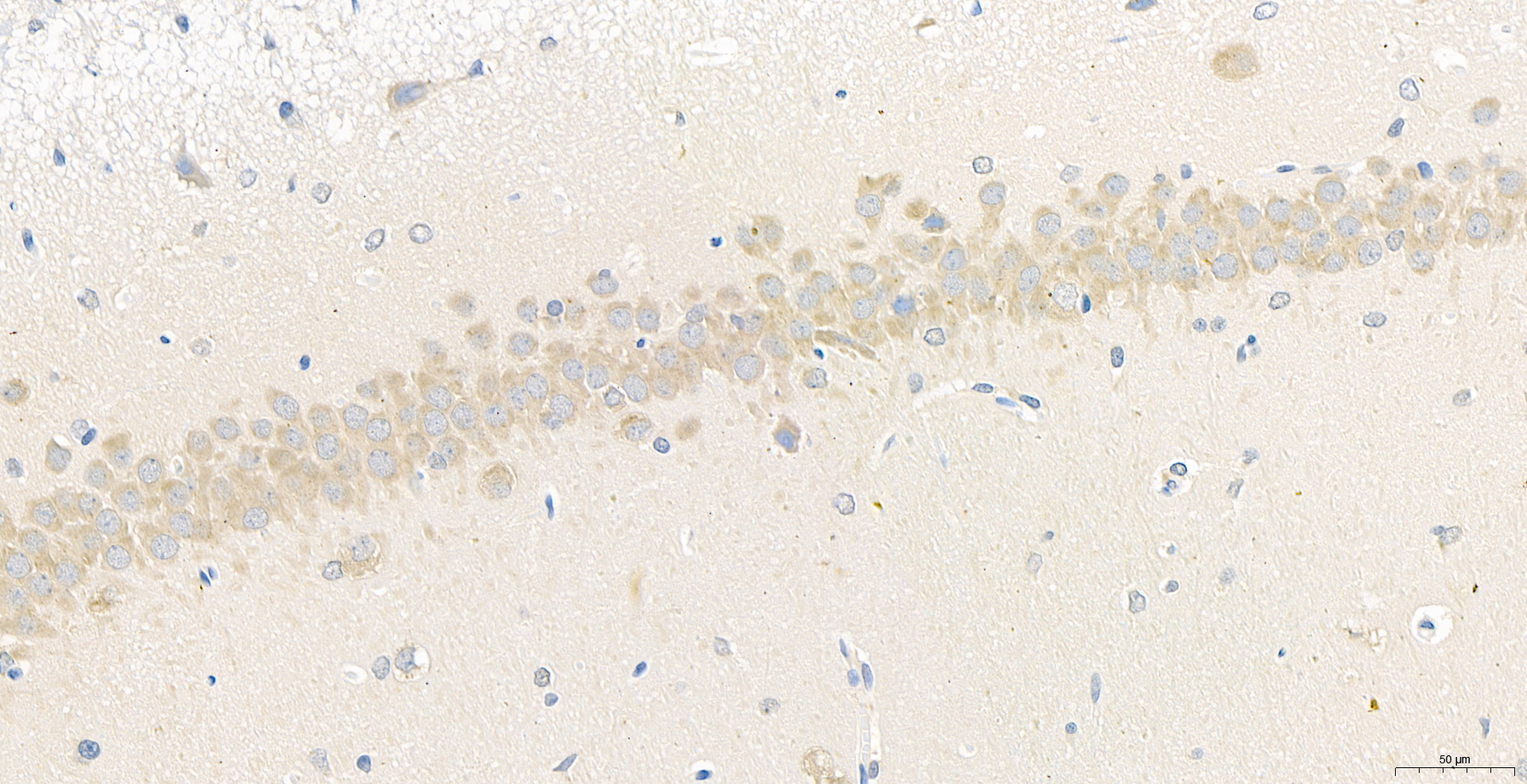

Supplement: S1 Raw data — (ZIP) [file pone.0305541.s002.zip › RAW DATA/FIG3/CAS3/10-100x_30.0x.jpg]

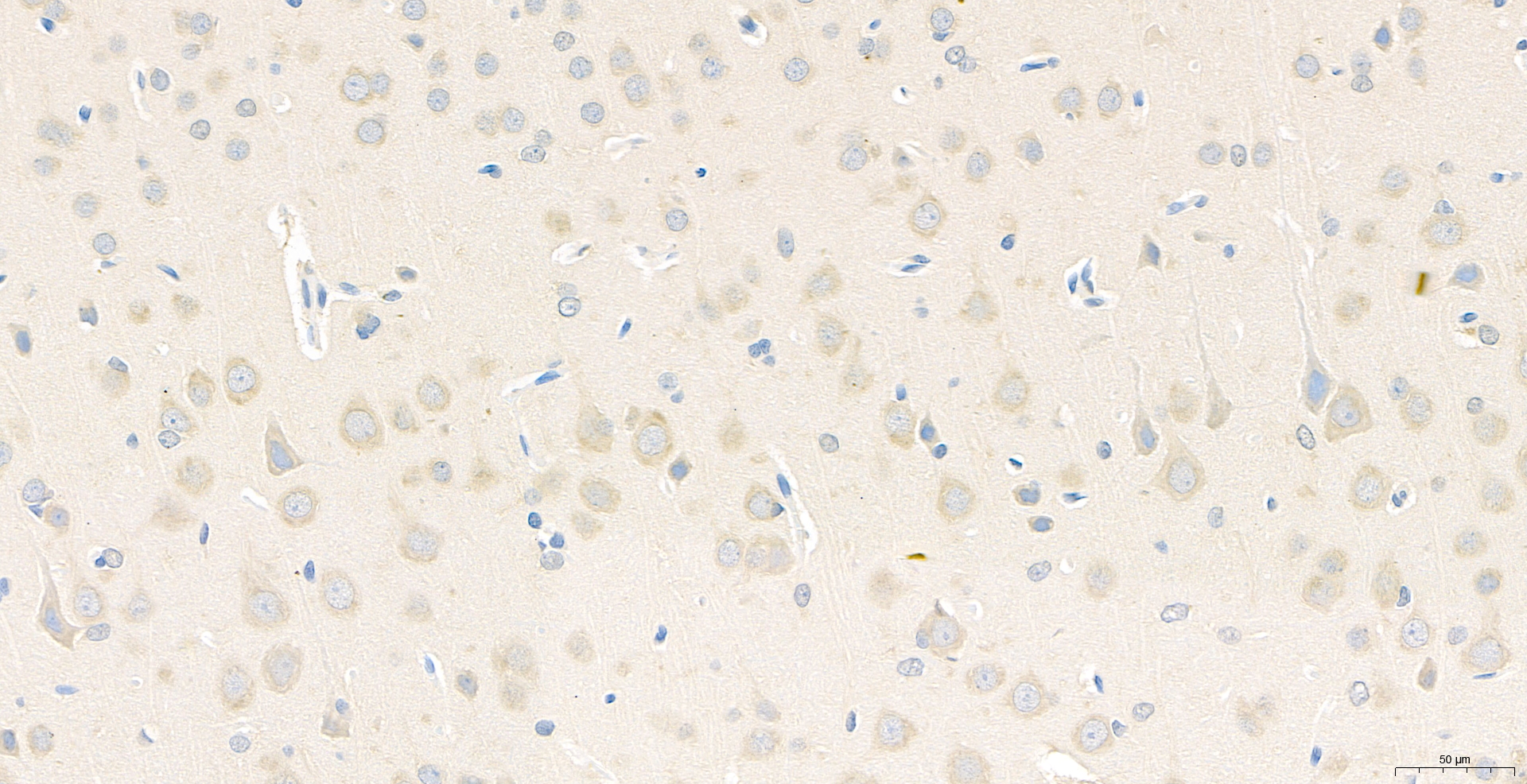

Supplement: S1 Raw data — (ZIP) [file pone.0305541.s002.zip › RAW DATA/FIG3/CAS3/20-100x_30.0x-HM.jpg]

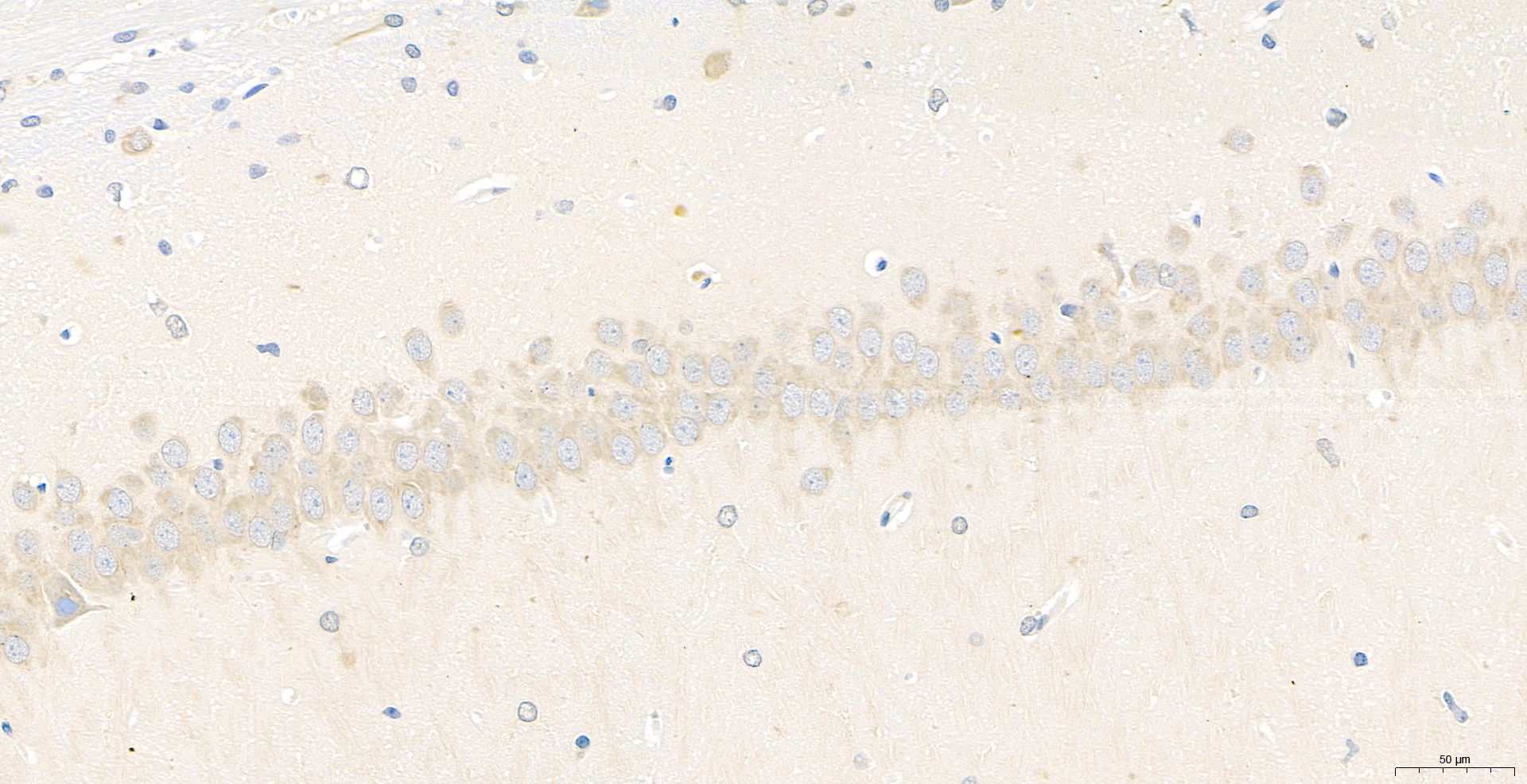

Supplement: S1 Raw data — (ZIP) [file pone.0305541.s002.zip › RAW DATA/FIG3/CAS3/20-100x_30.0x.jpg]

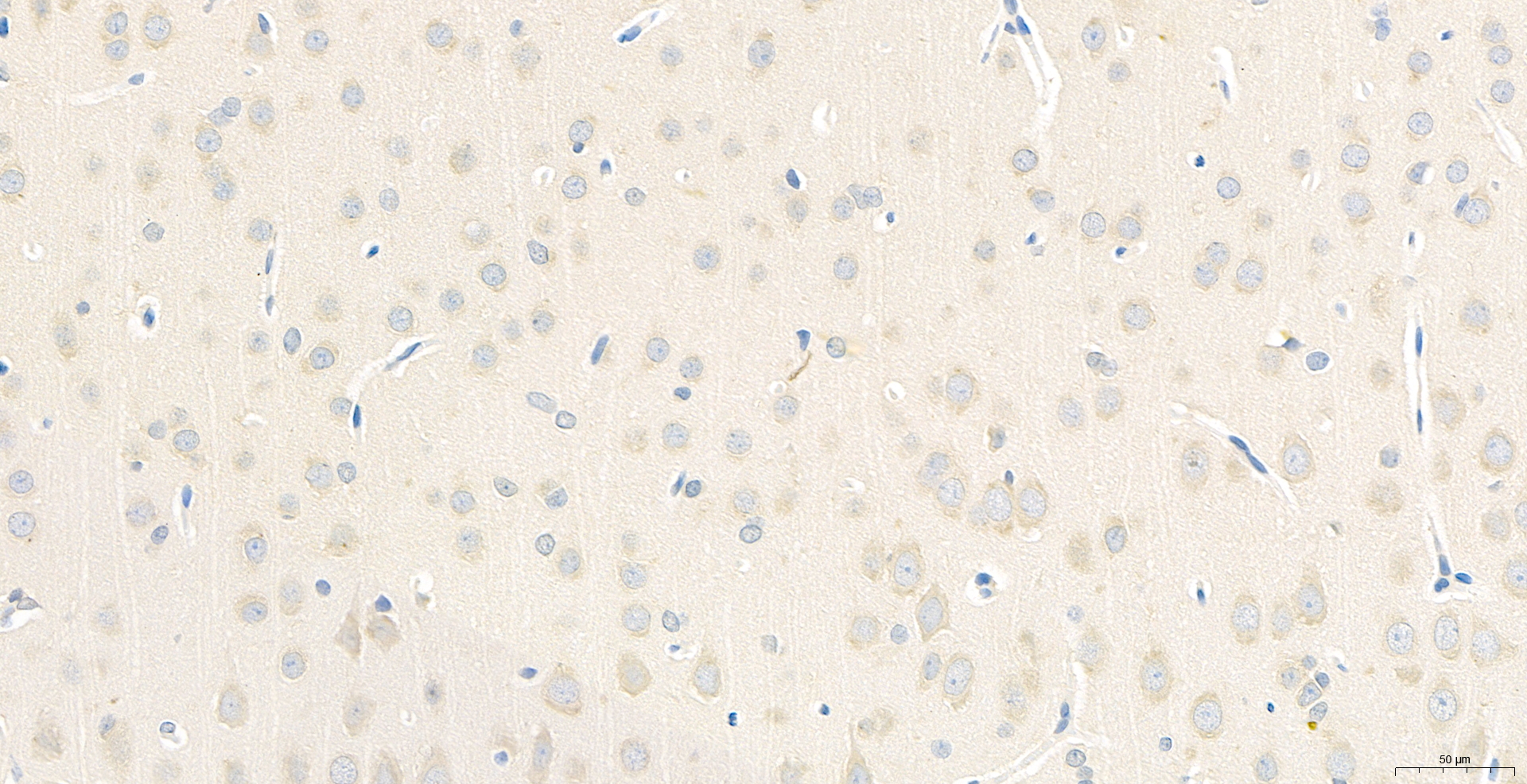

Supplement: S1 Raw data — (ZIP) [file pone.0305541.s002.zip › RAW DATA/FIG3/CAS3/K-100x_30.0x-HM.jpg]

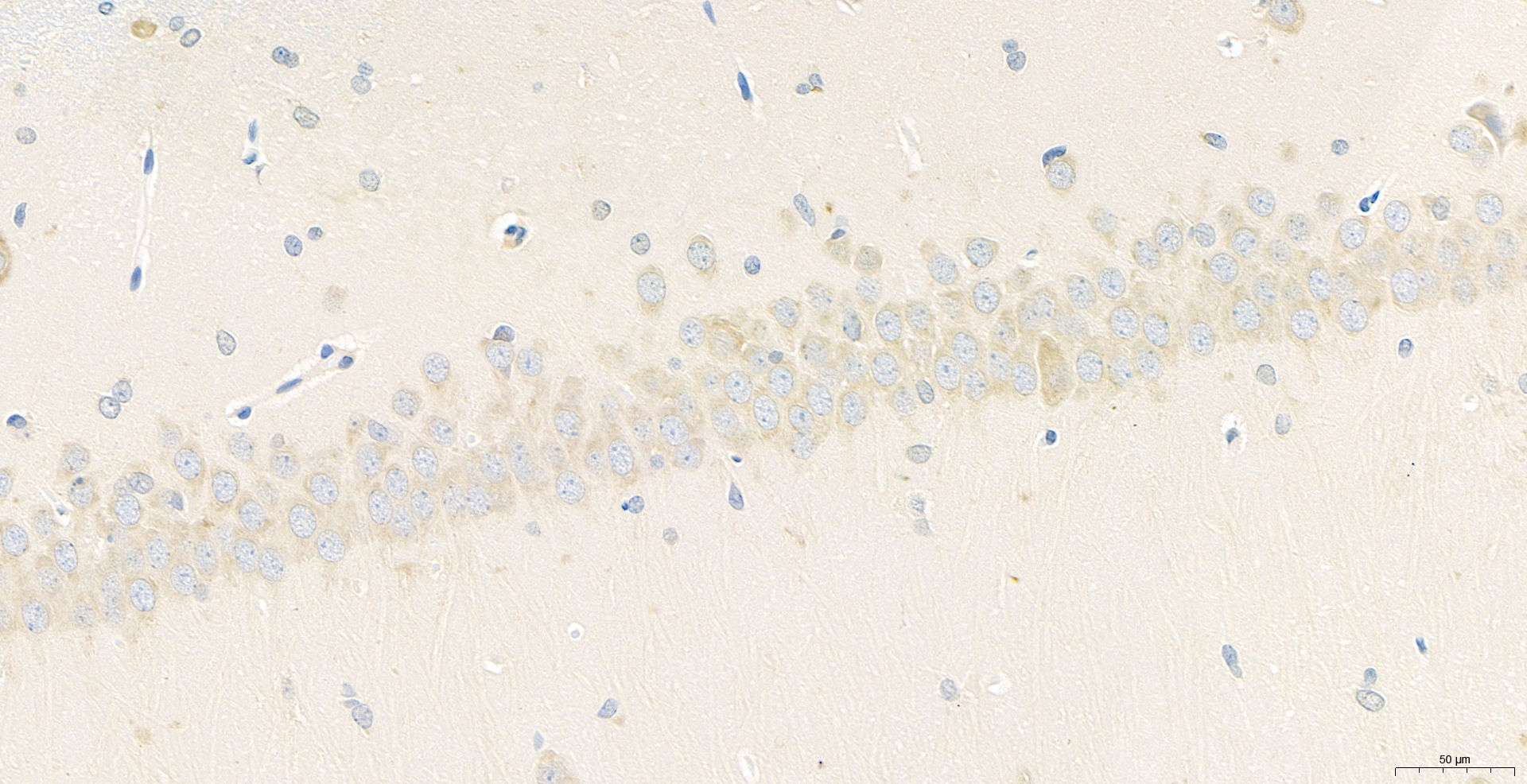

Supplement: S1 Raw data — (ZIP) [file pone.0305541.s002.zip › RAW DATA/FIG3/CAS3/K-100x_30.0x.jpg]

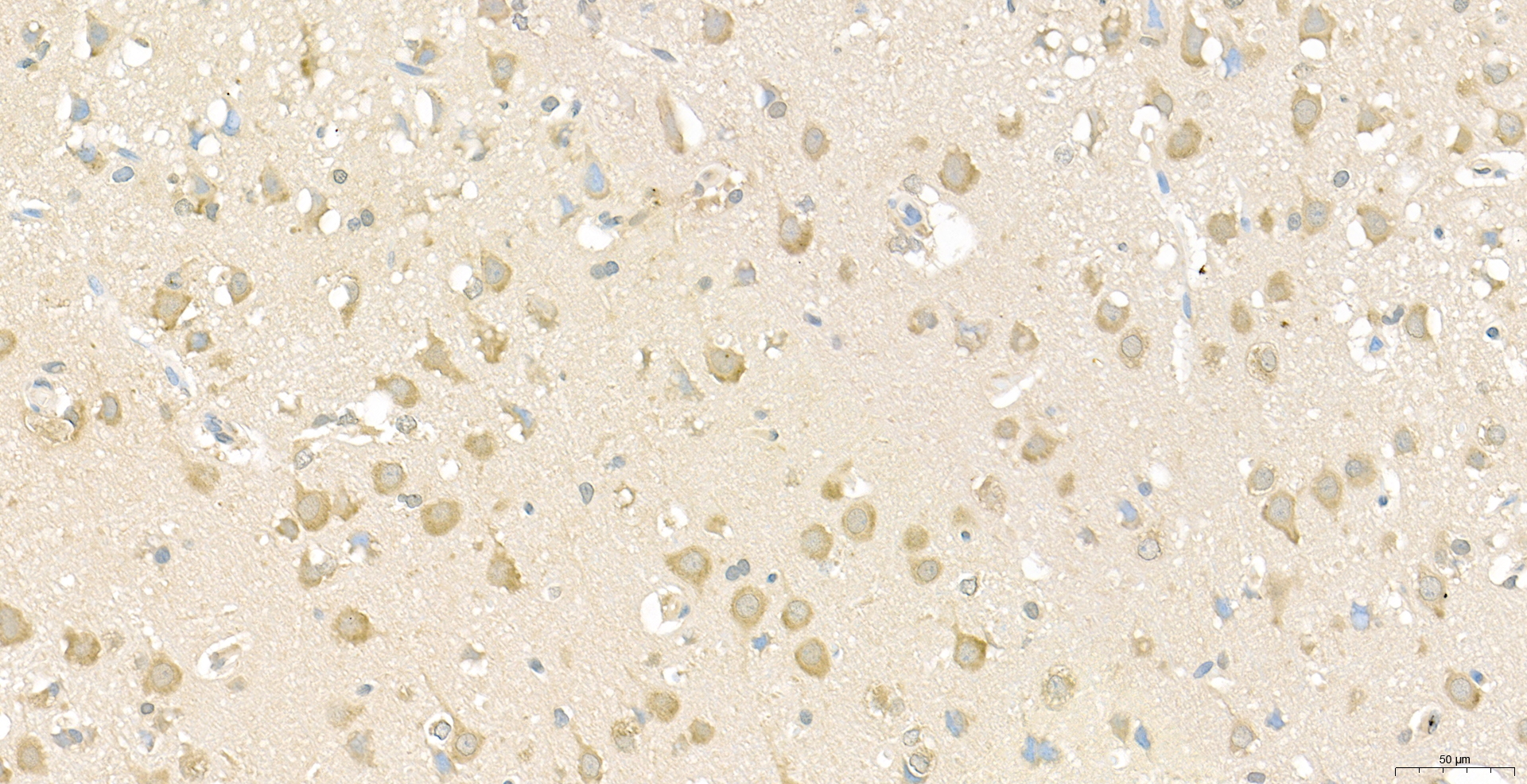

Supplement: S1 Raw data — (ZIP) [file pone.0305541.s002.zip › RAW DATA/FIG3/CAS3/M-100x_30.0x-HM.jpg]

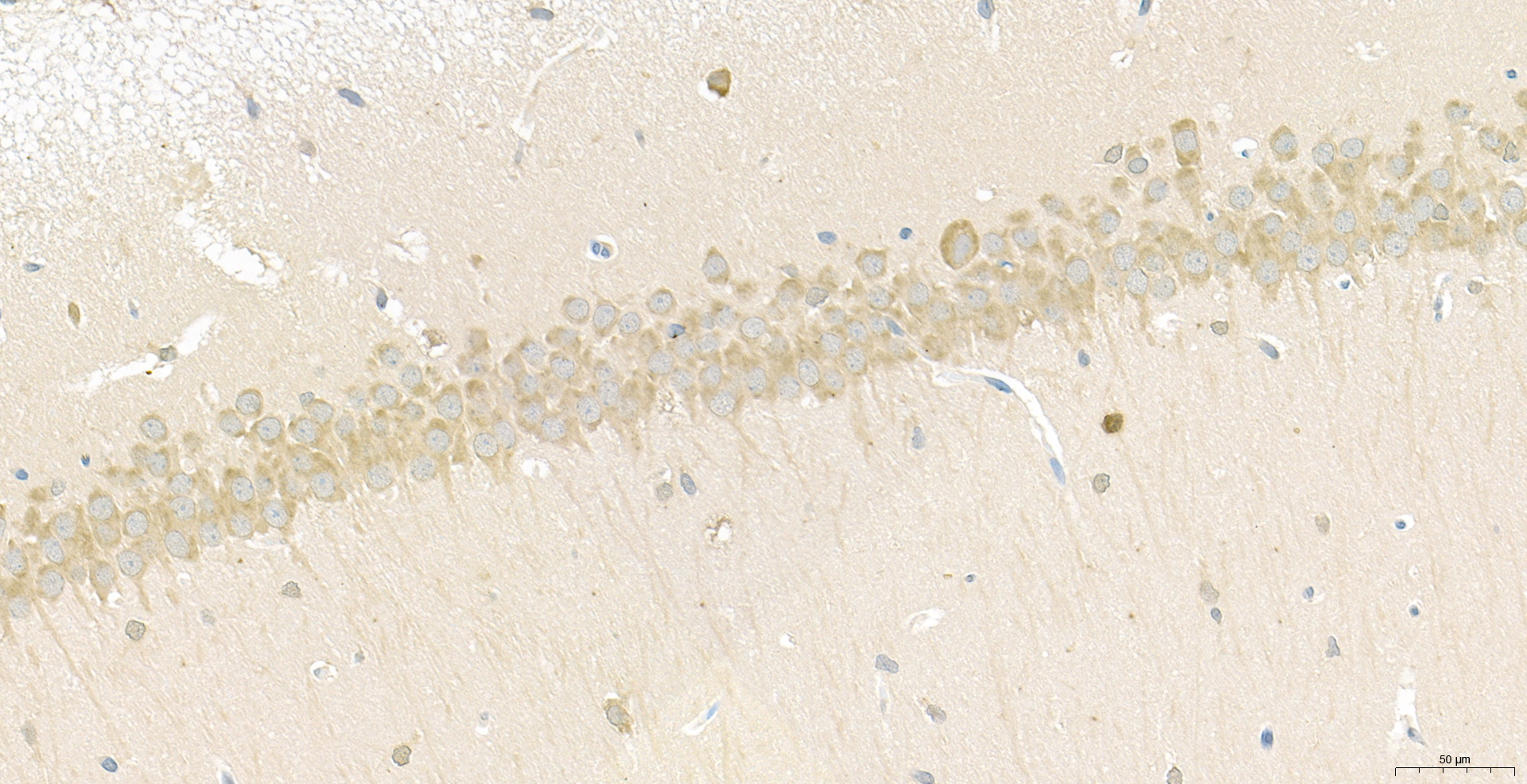

Supplement: S1 Raw data — (ZIP) [file pone.0305541.s002.zip › RAW DATA/FIG3/CAS3/M-100x_30.0x.jpg]

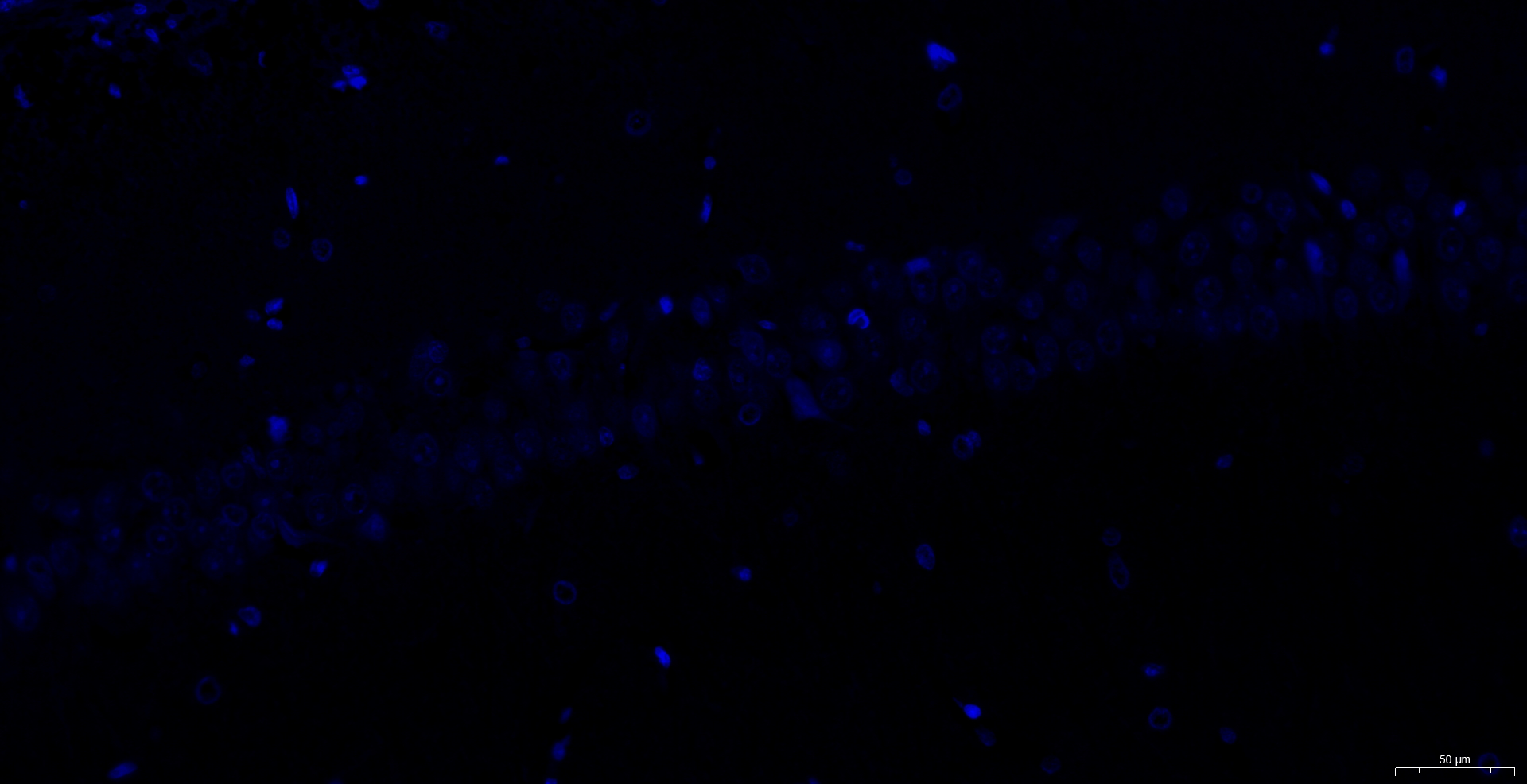

Supplement: S1 Raw data — (ZIP) [file pone.0305541.s002.zip › RAW DATA/FIG3/TUNEL CAI/10-TUNEL_30.0x-1.jpg]

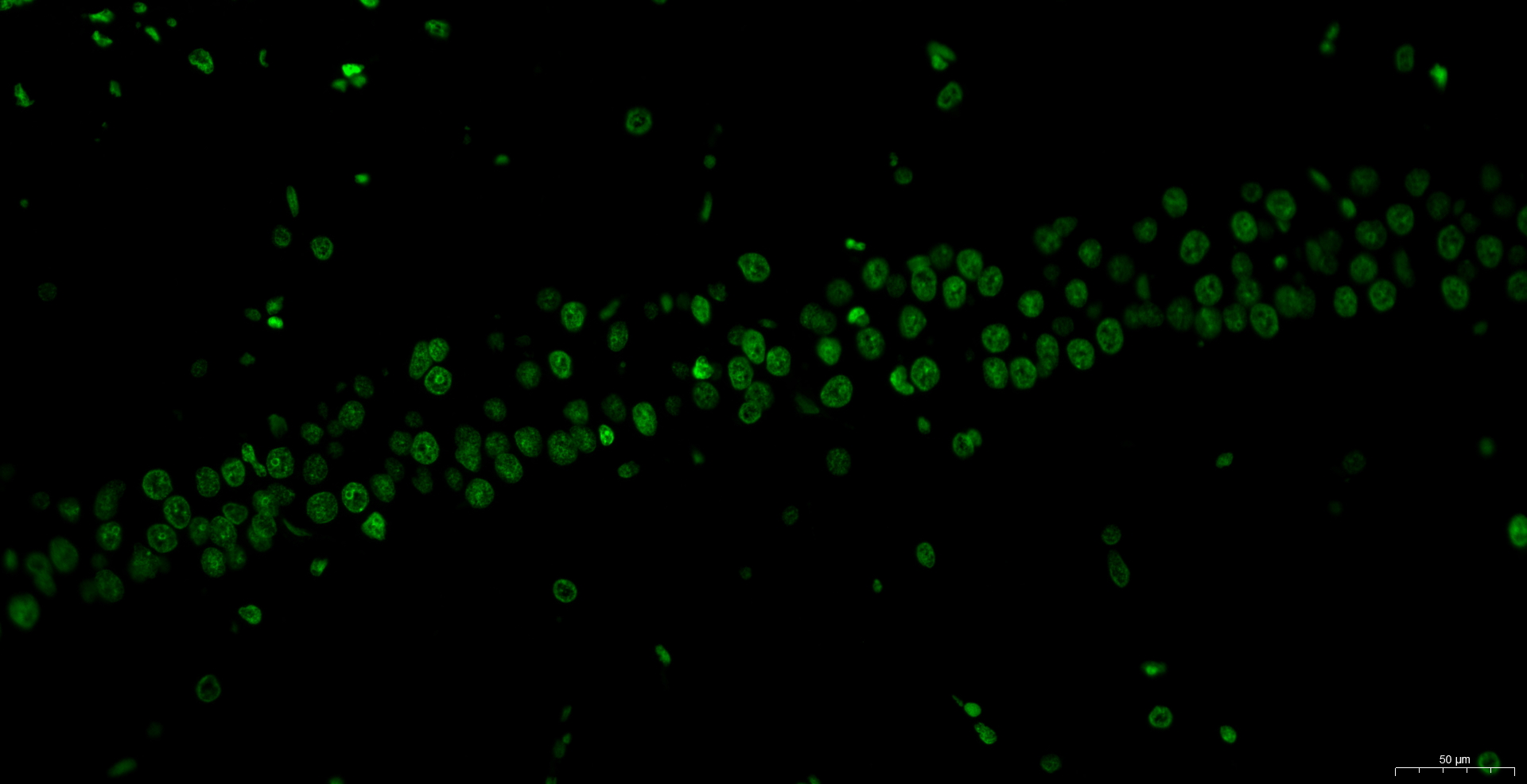

Supplement: S1 Raw data — (ZIP) [file pone.0305541.s002.zip › RAW DATA/FIG3/TUNEL CAI/10-TUNEL_30.0x-2.jpg]

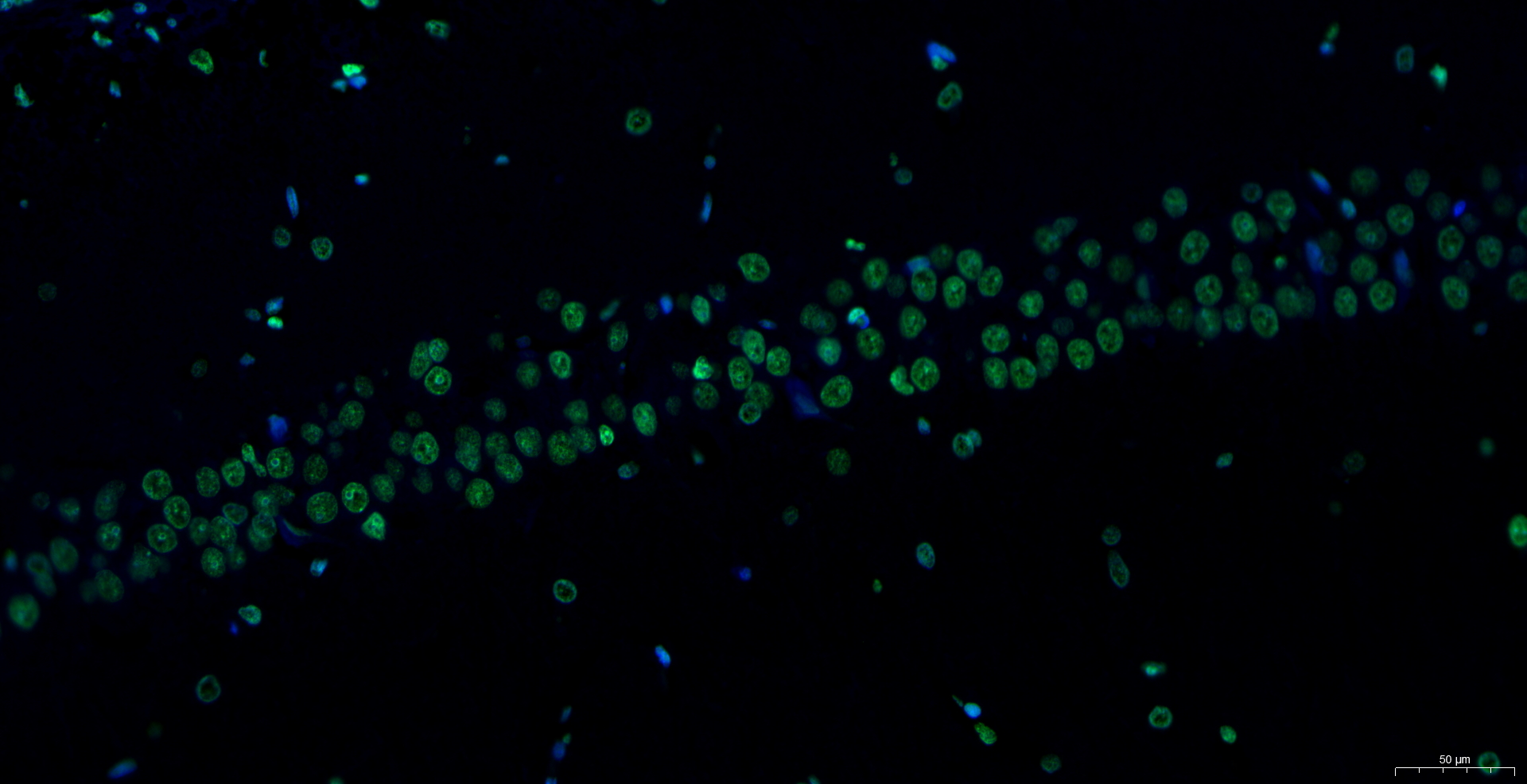

Supplement: S1 Raw data — (ZIP) [file pone.0305541.s002.zip › RAW DATA/FIG3/TUNEL CAI/10-TUNEL_30.0x.jpg]

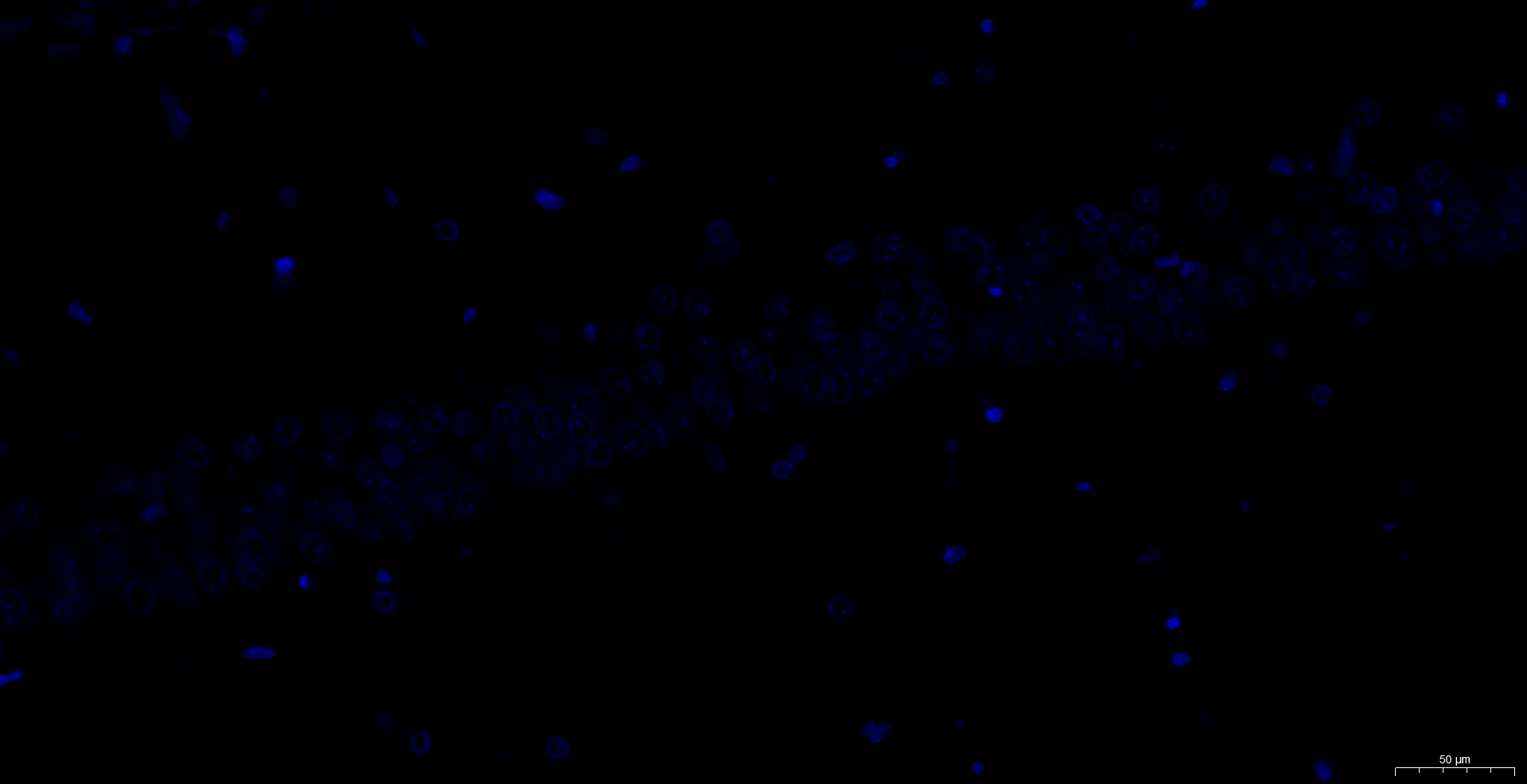

Supplement: S1 Raw data — (ZIP) [file pone.0305541.s002.zip › RAW DATA/FIG3/TUNEL CAI/20-TUNEL_30.0x-1.jpg]

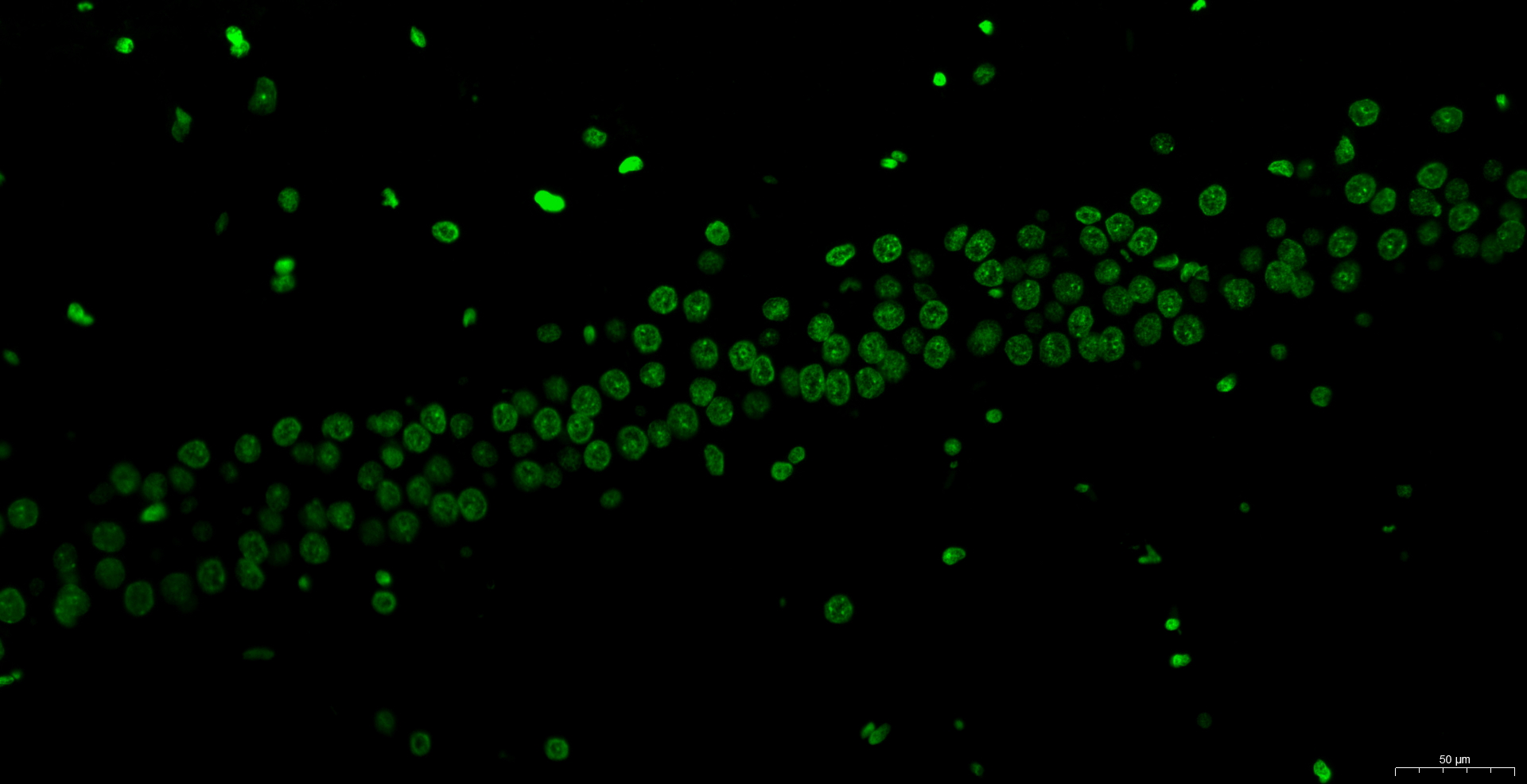

Supplement: S1 Raw data — (ZIP) [file pone.0305541.s002.zip › RAW DATA/FIG3/TUNEL CAI/20-TUNEL_30.0x-2.jpg]

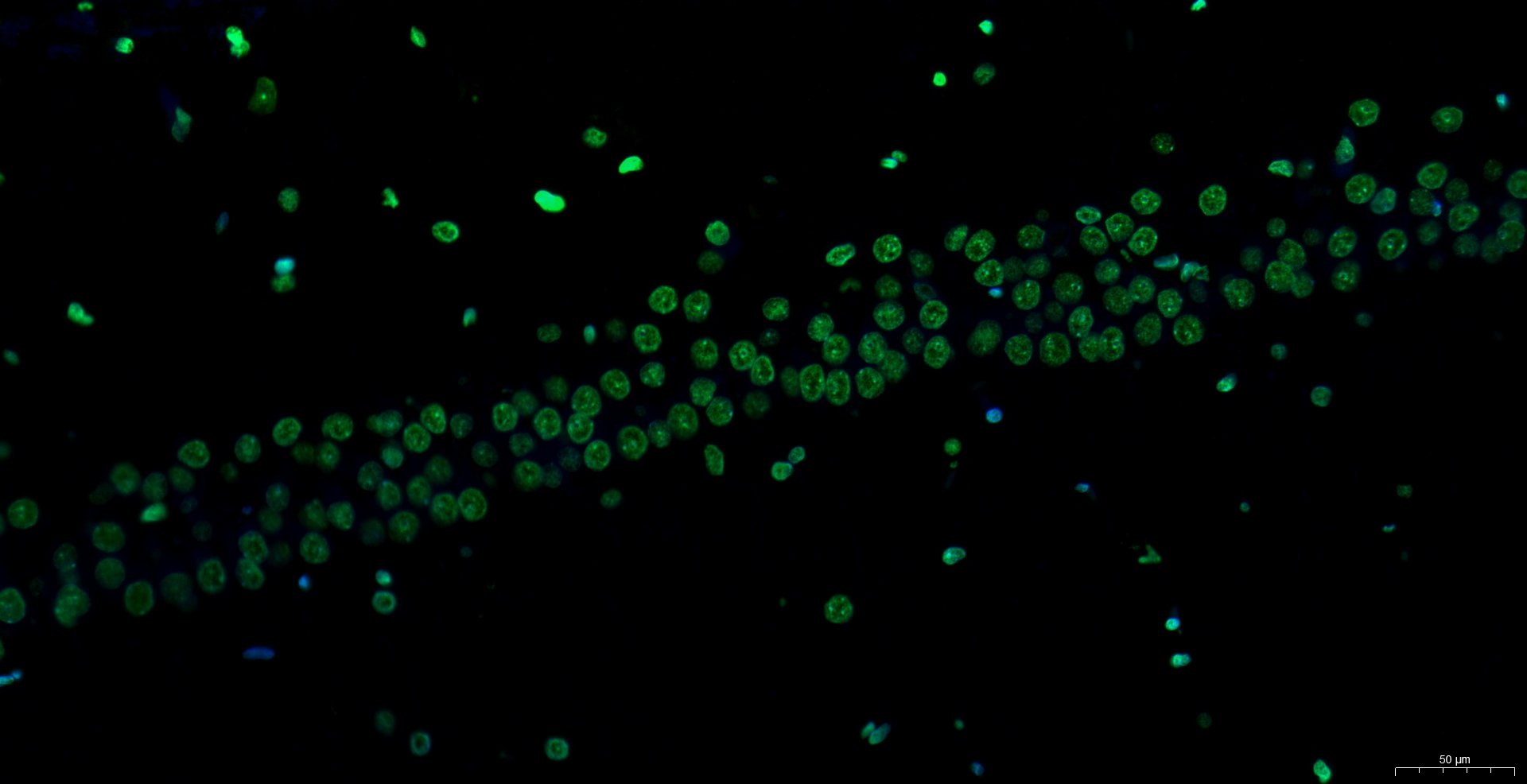

Supplement: S1 Raw data — (ZIP) [file pone.0305541.s002.zip › RAW DATA/FIG3/TUNEL CAI/20-TUNEL_30.0x.jpg]

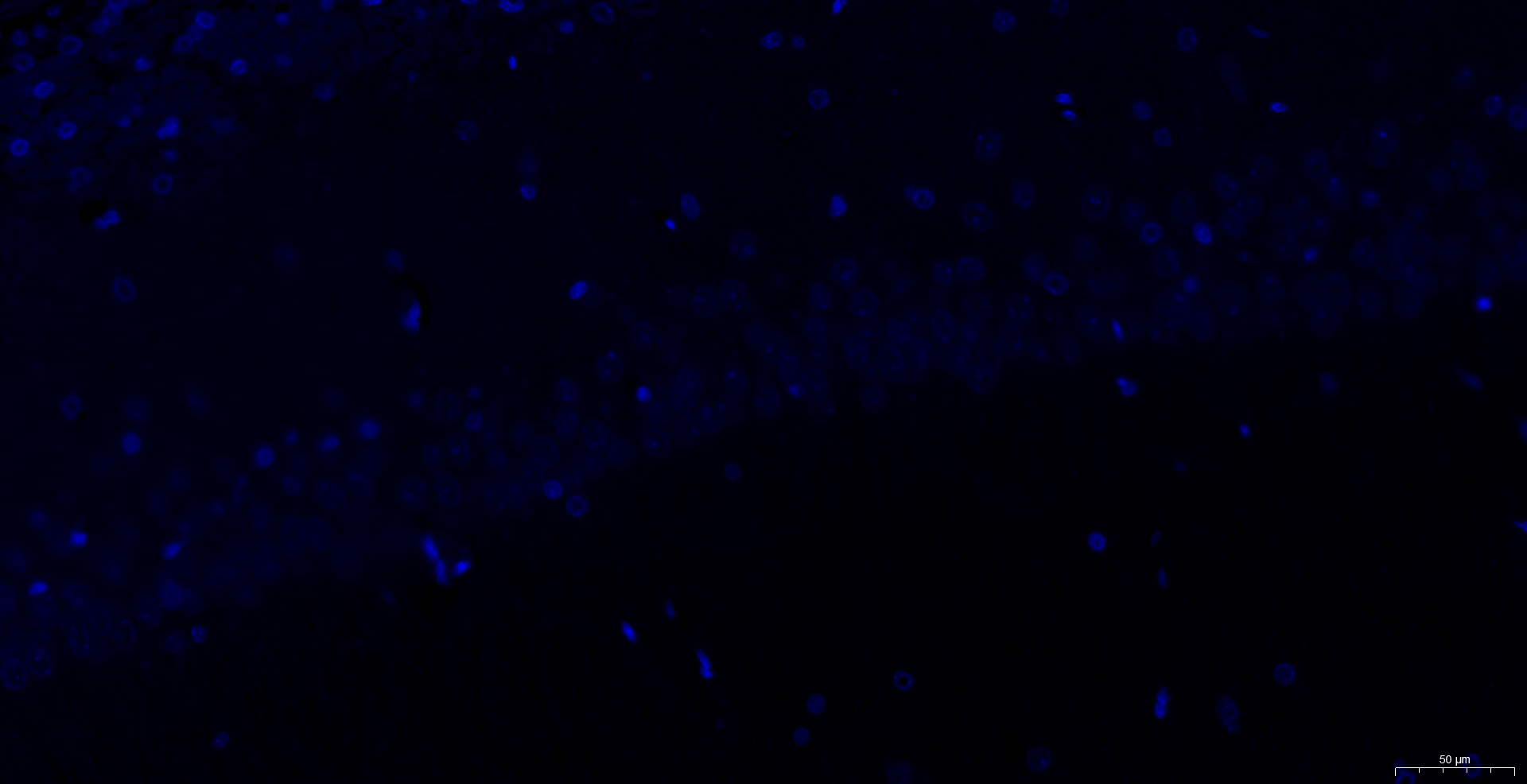

Supplement: S1 Raw data — (ZIP) [file pone.0305541.s002.zip › RAW DATA/FIG3/TUNEL CAI/K-TUNEL_30.0x-1.jpg]

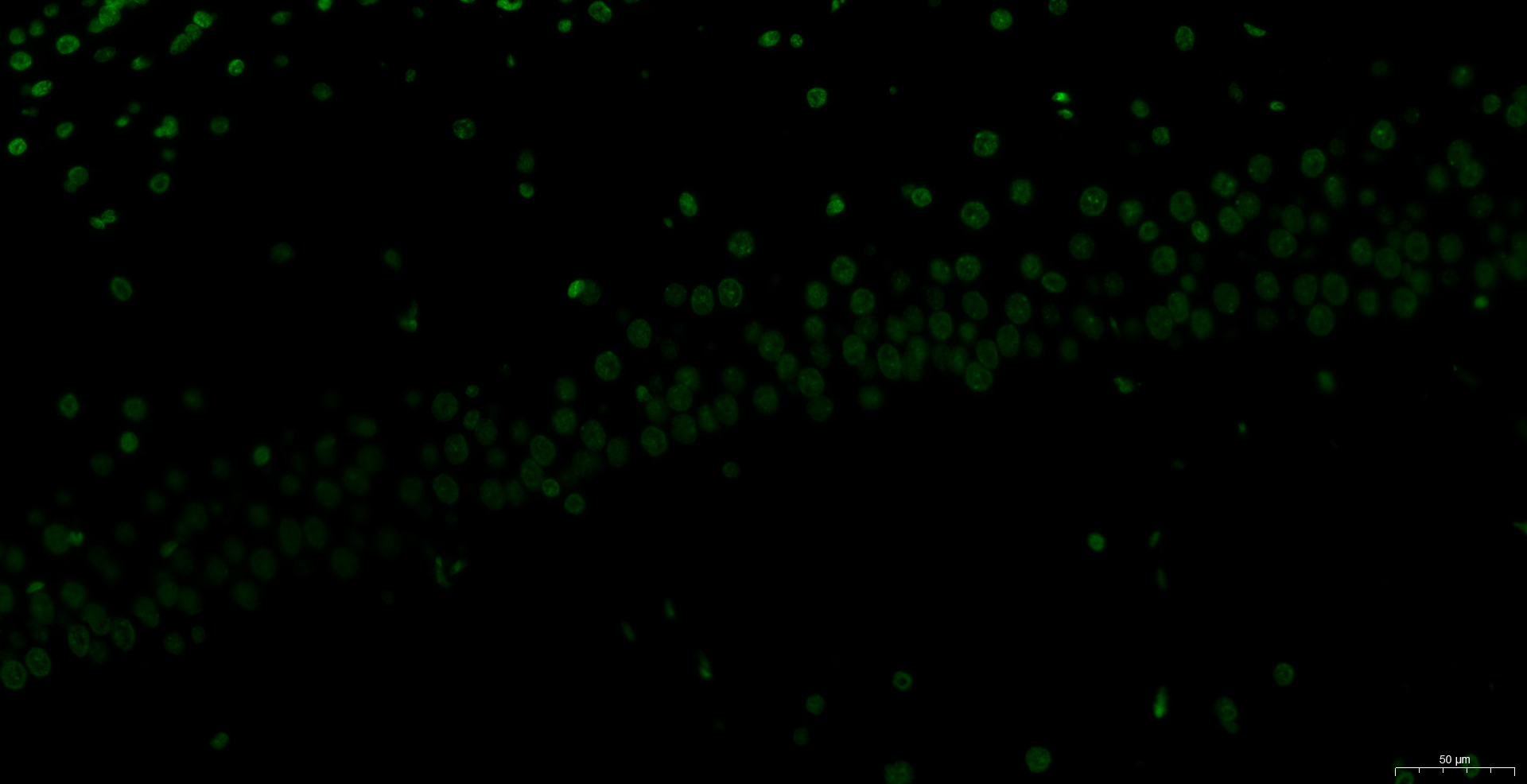

Supplement: S1 Raw data — (ZIP) [file pone.0305541.s002.zip › RAW DATA/FIG3/TUNEL CAI/K-TUNEL_30.0x-2.jpg]

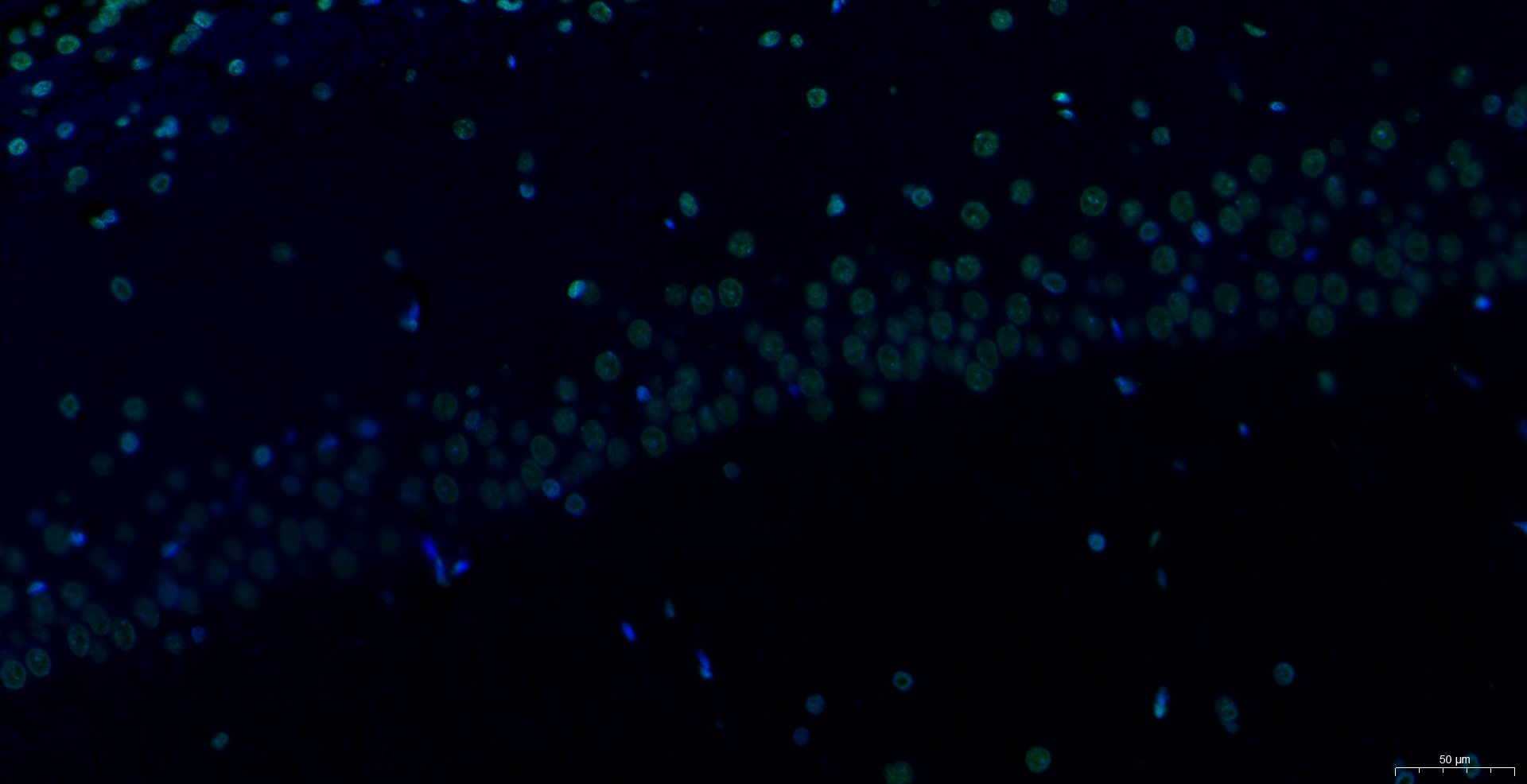

Supplement: S1 Raw data — (ZIP) [file pone.0305541.s002.zip › RAW DATA/FIG3/TUNEL CAI/K-TUNEL_30.0x.jpg]

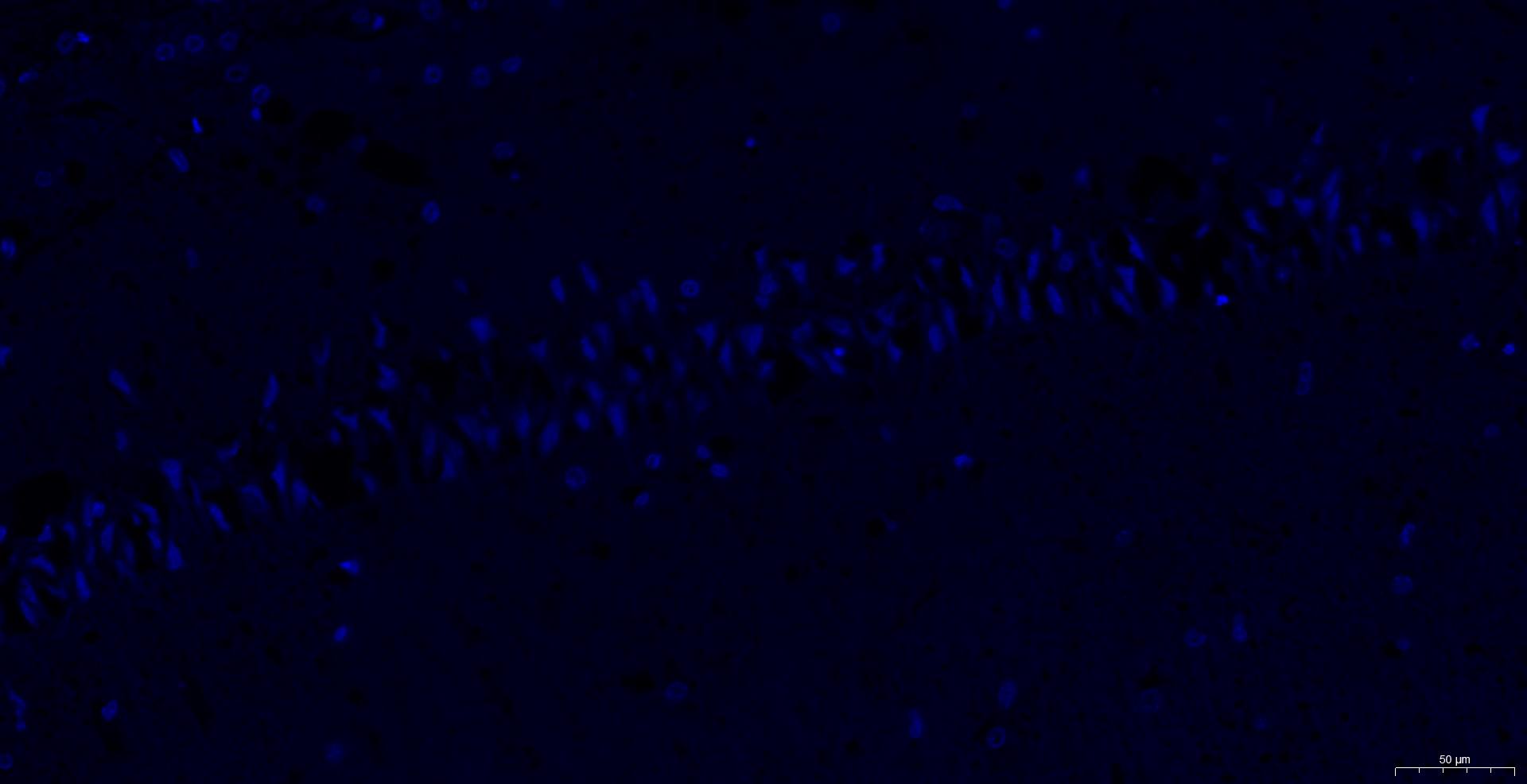

Supplement: S1 Raw data — (ZIP) [file pone.0305541.s002.zip › RAW DATA/FIG3/TUNEL CAI/M-TUNEL_30.0x-1.jpg]

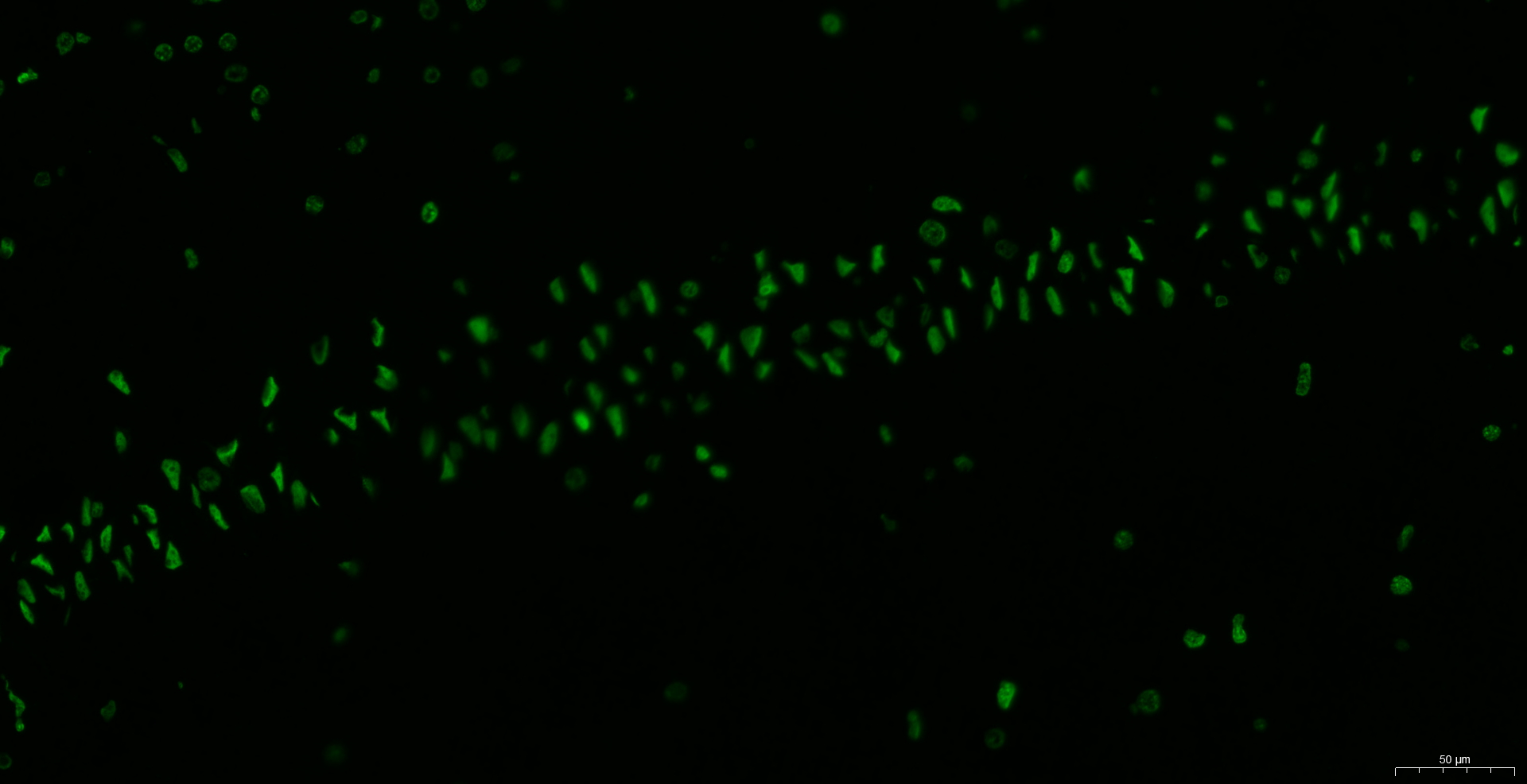

Supplement: S1 Raw data — (ZIP) [file pone.0305541.s002.zip › RAW DATA/FIG3/TUNEL CAI/M-TUNEL_30.0x-2.jpg]

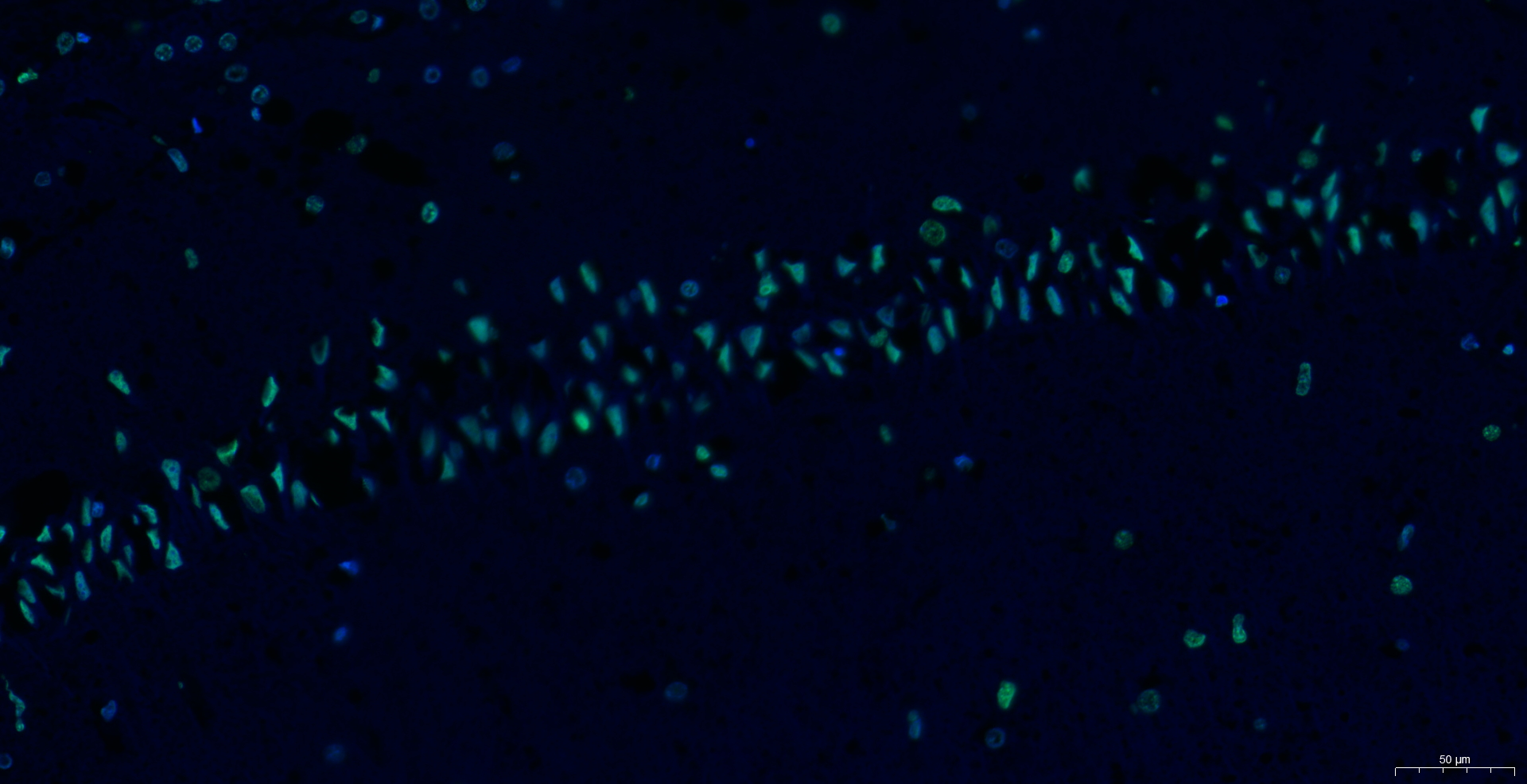

Supplement: S1 Raw data — (ZIP) [file pone.0305541.s002.zip › RAW DATA/FIG3/TUNEL CAI/M-TUNEL_30.0x.jpg]

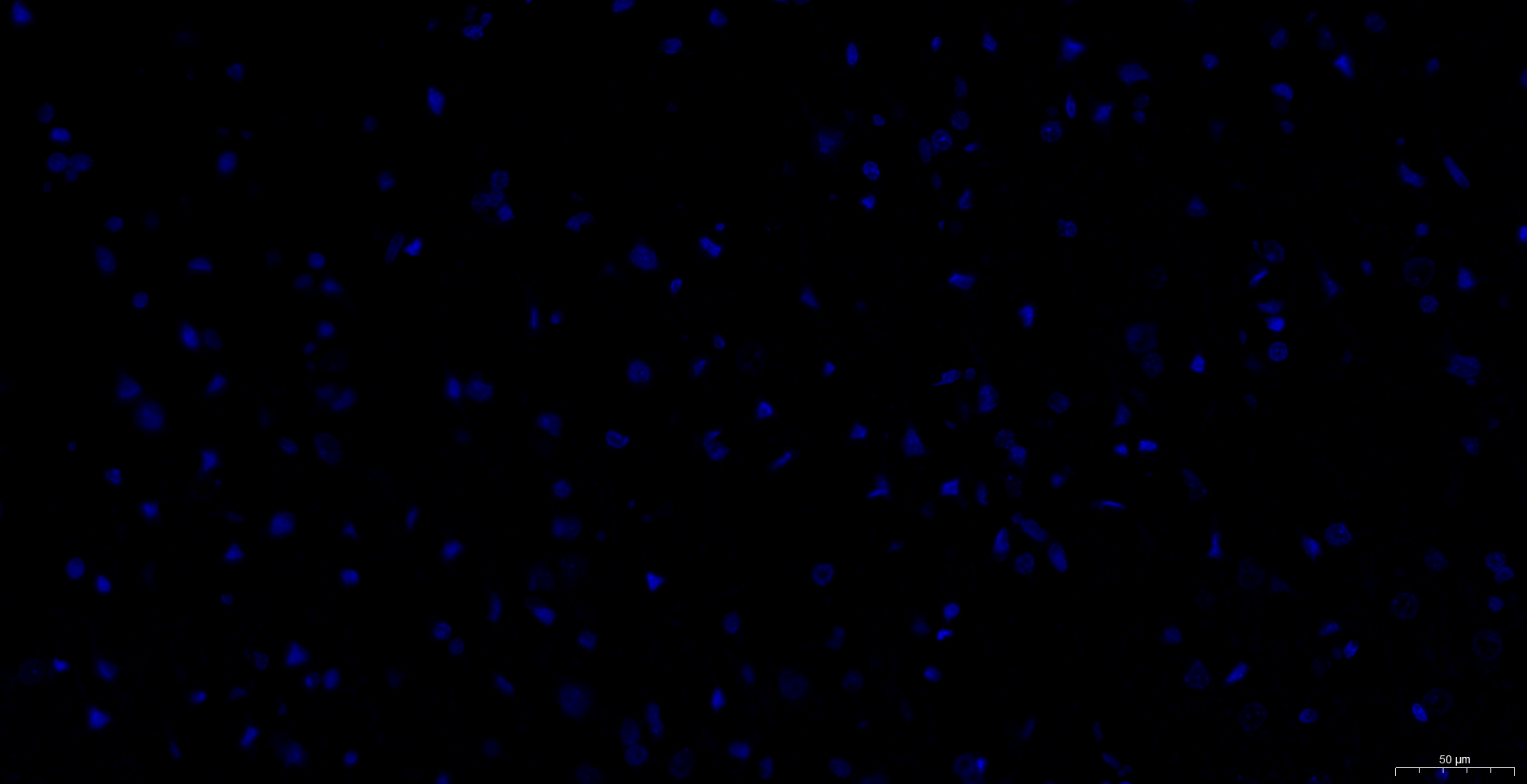

Supplement: S1 Raw data — (ZIP) [file pone.0305541.s002.zip › RAW DATA/FIG3/TUNEL COR/10-TUNEL_30.0x-1.jpg]

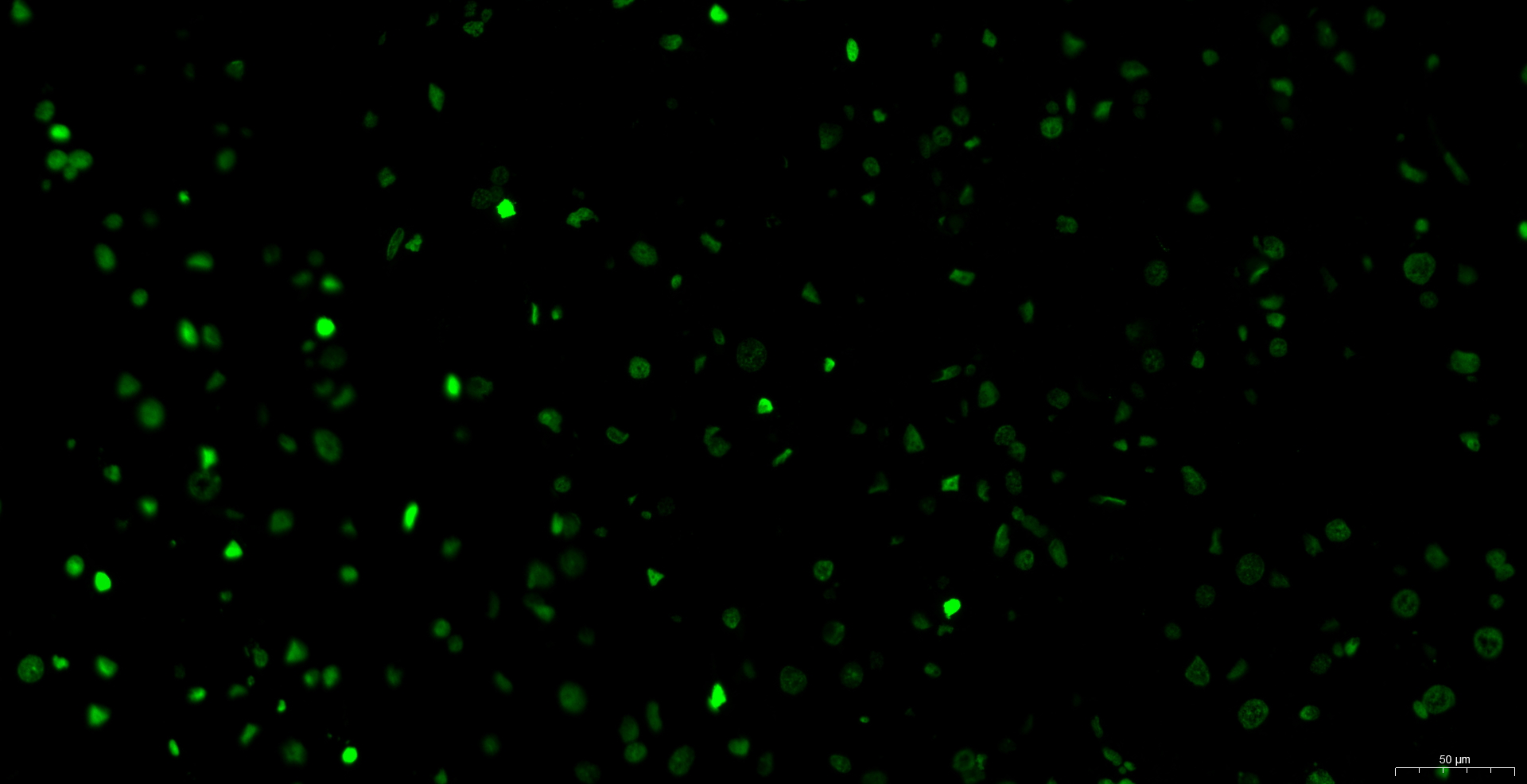

Supplement: S1 Raw data — (ZIP) [file pone.0305541.s002.zip › RAW DATA/FIG3/TUNEL COR/10-TUNEL_30.0x-2.jpg]

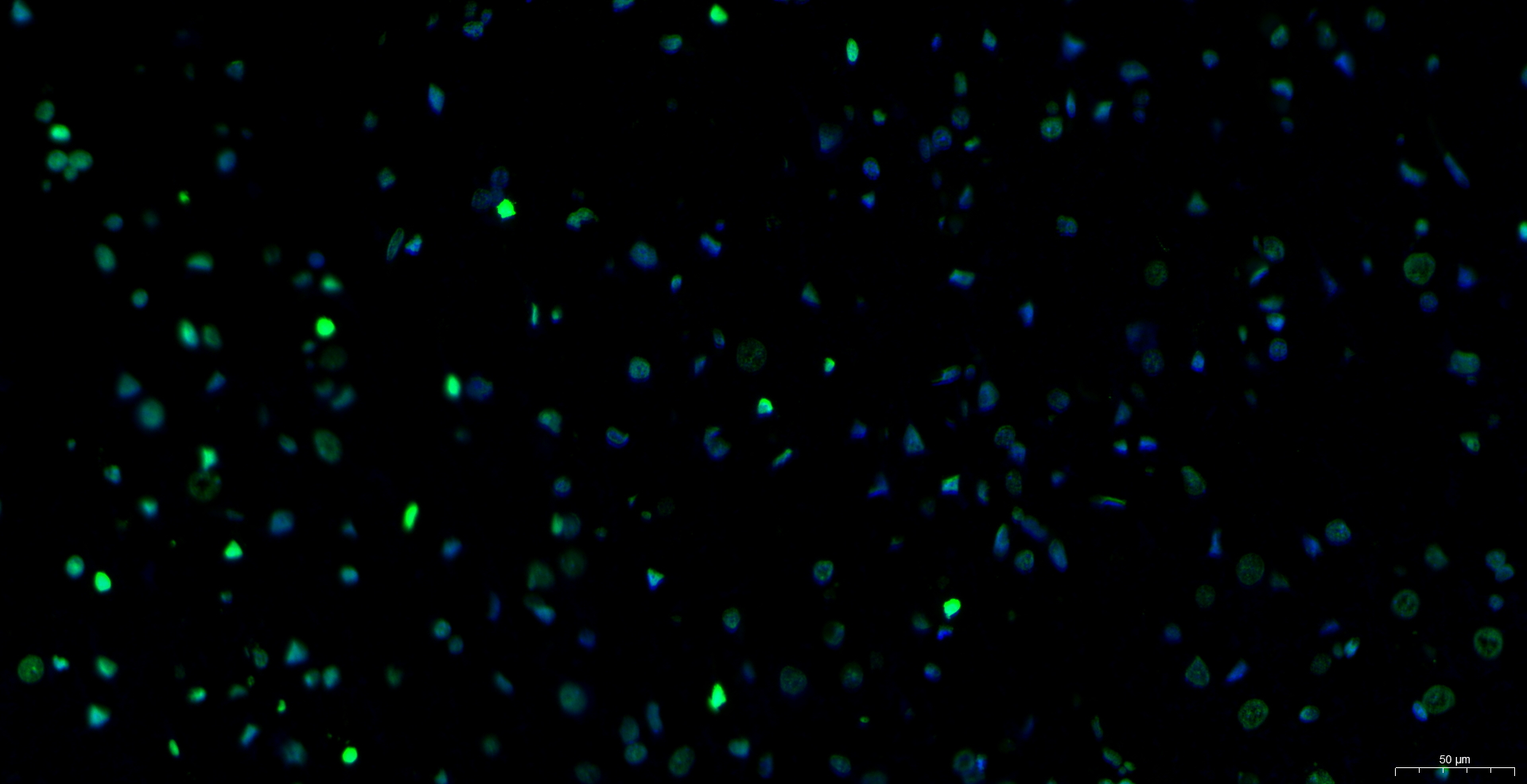

Supplement: S1 Raw data — (ZIP) [file pone.0305541.s002.zip › RAW DATA/FIG3/TUNEL COR/10-TUNEL_30.0x.jpg]

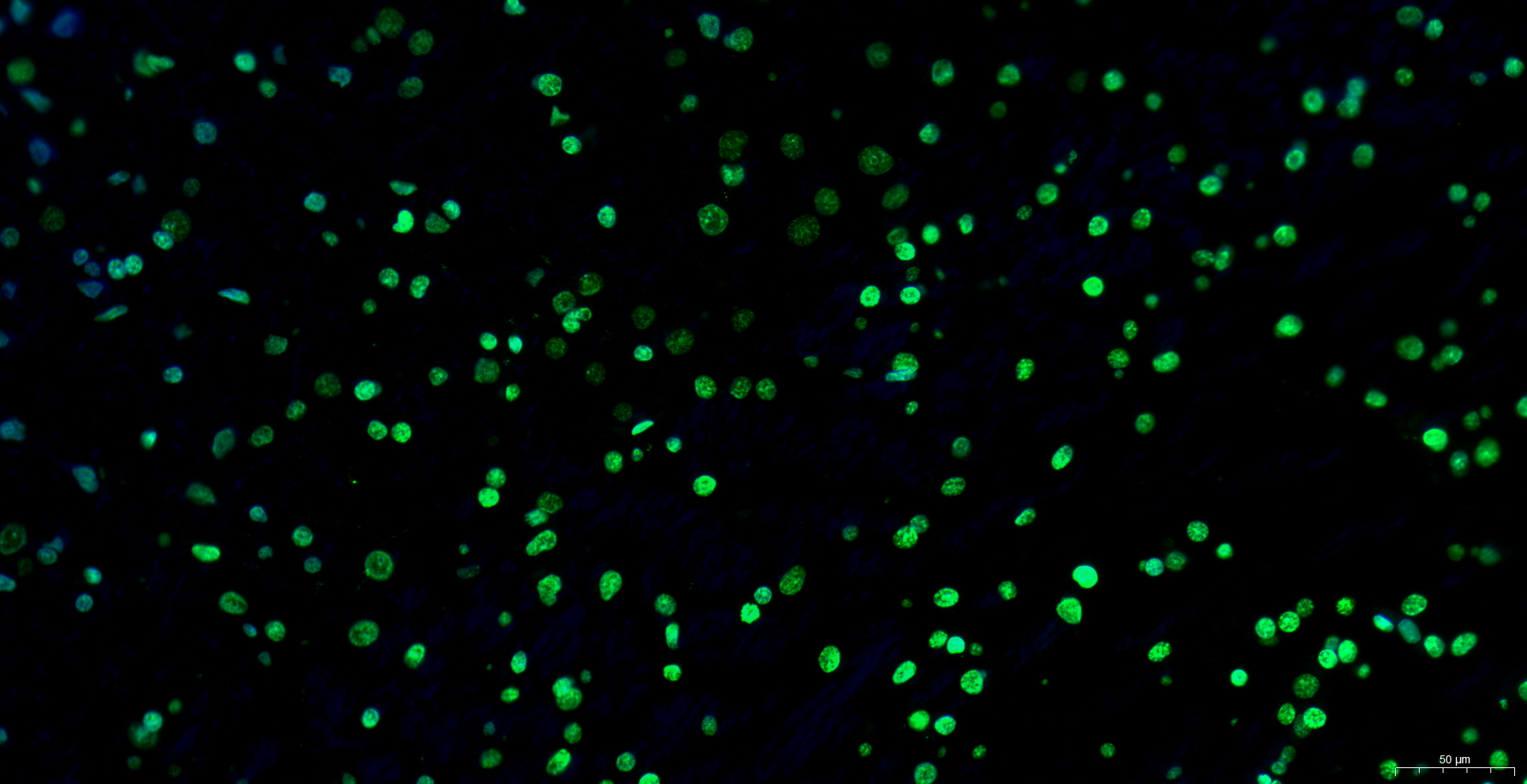

Supplement: S1 Raw data — (ZIP) [file pone.0305541.s002.zip › RAW DATA/FIG3/TUNEL COR/20-TUNEL_30.0x-HM.jpg]

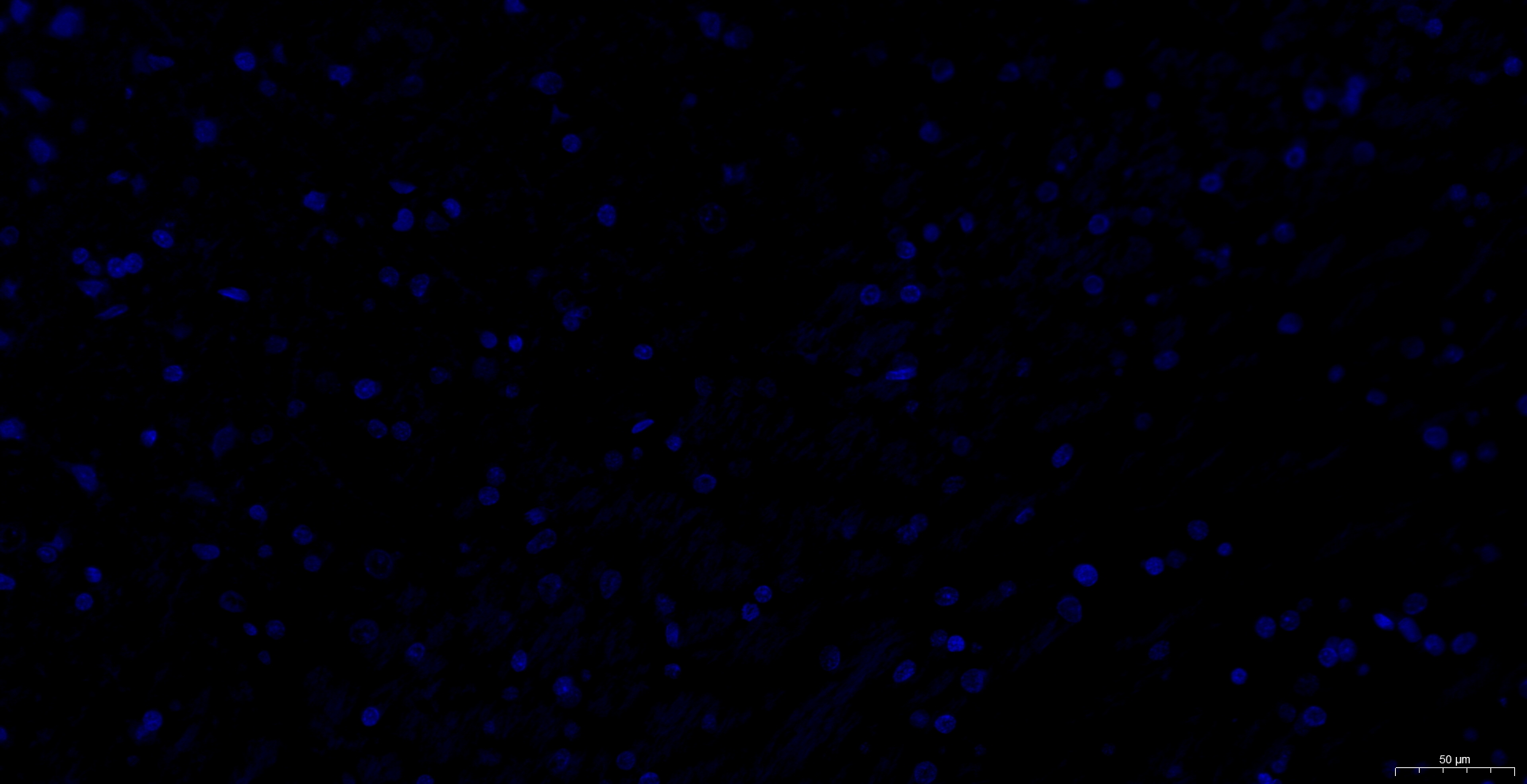

Supplement: S1 Raw data — (ZIP) [file pone.0305541.s002.zip › RAW DATA/FIG3/TUNEL COR/20-TUNEL_30.0x-HM1.jpg]

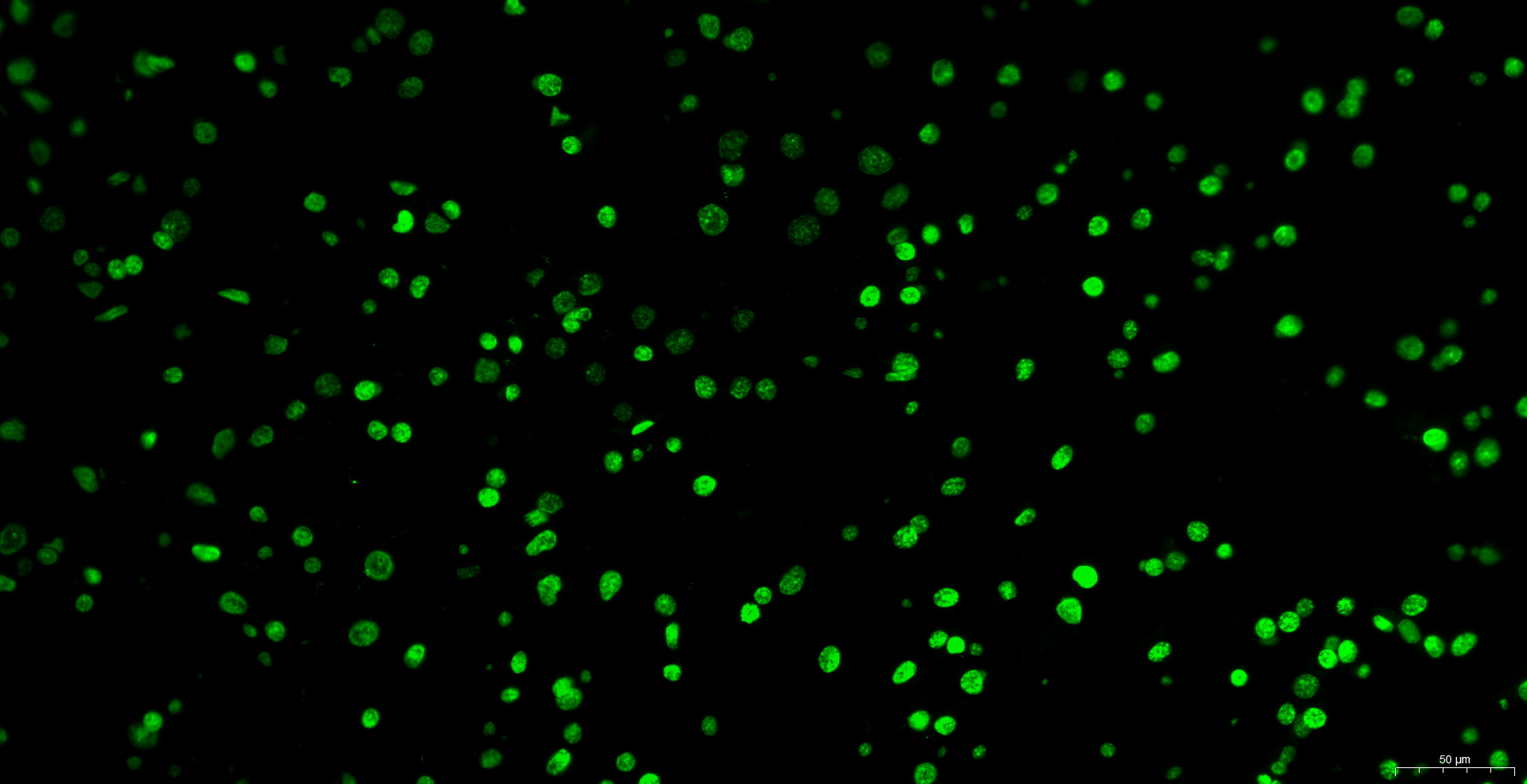

Supplement: S1 Raw data — (ZIP) [file pone.0305541.s002.zip › RAW DATA/FIG3/TUNEL COR/20-TUNEL_30.0x-HM2.jpg]

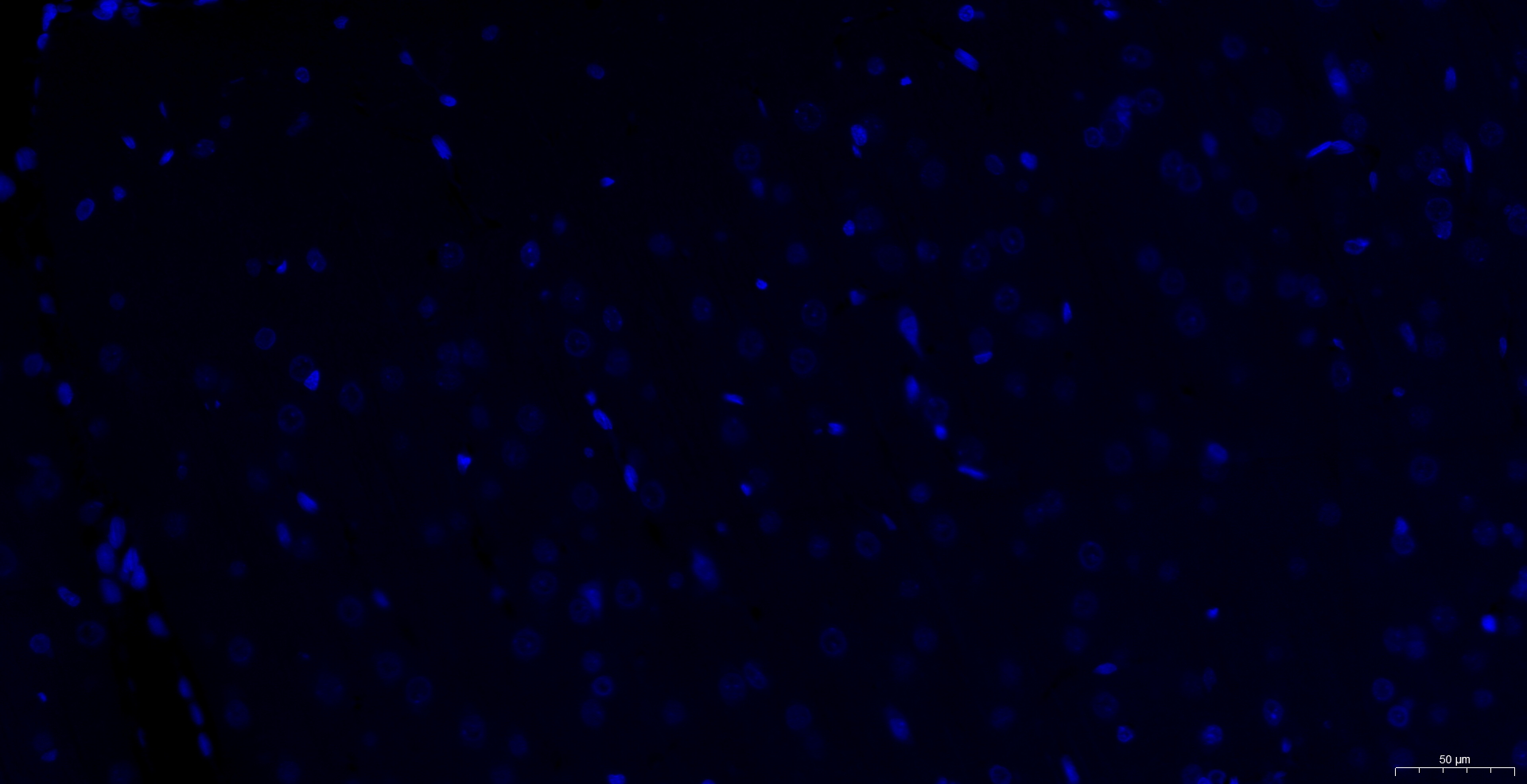

Supplement: S1 Raw data — (ZIP) [file pone.0305541.s002.zip › RAW DATA/FIG3/TUNEL COR/K-TUNEL_30.0x-1.jpg]

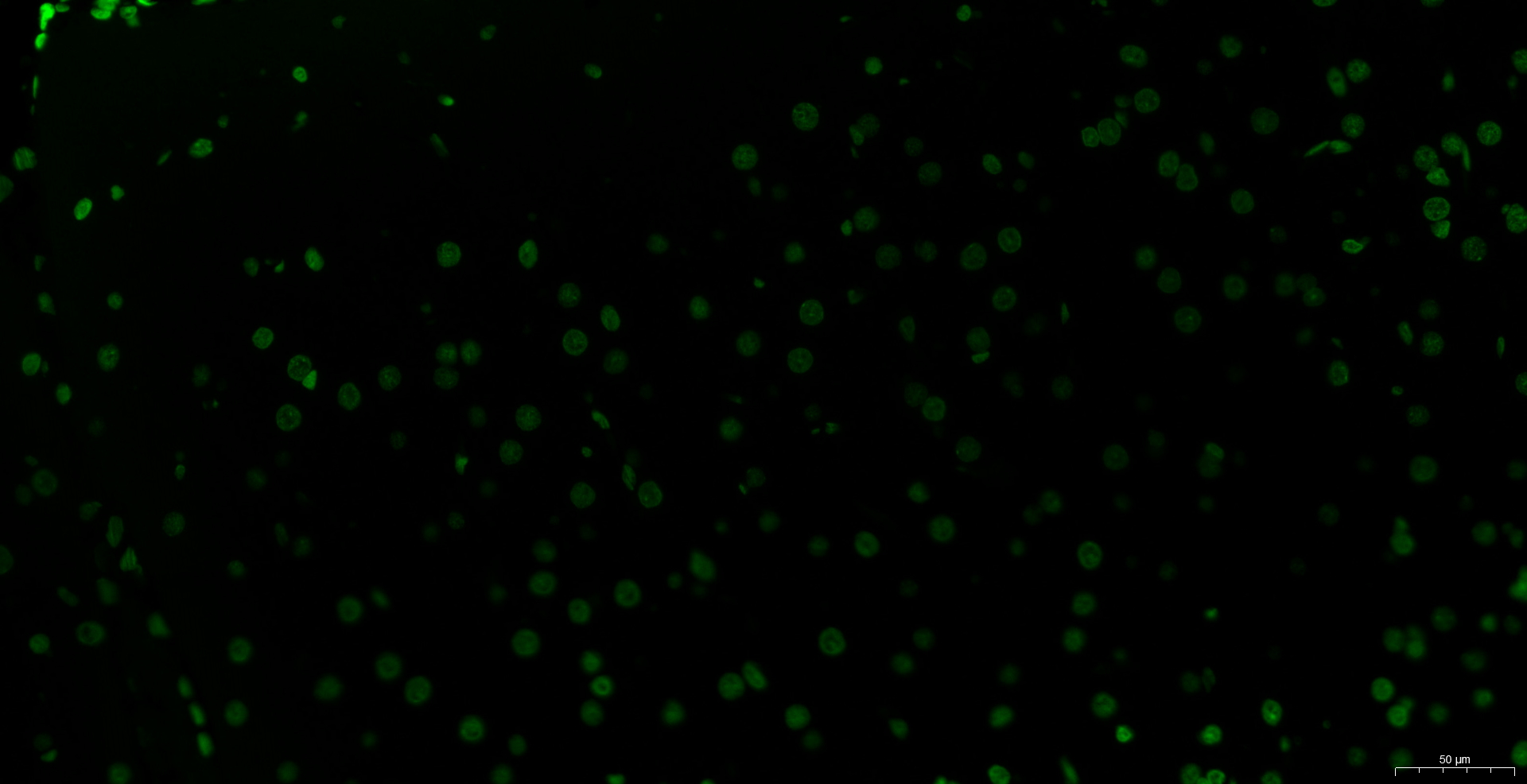

Supplement: S1 Raw data — (ZIP) [file pone.0305541.s002.zip › RAW DATA/FIG3/TUNEL COR/K-TUNEL_30.0x-2.jpg]

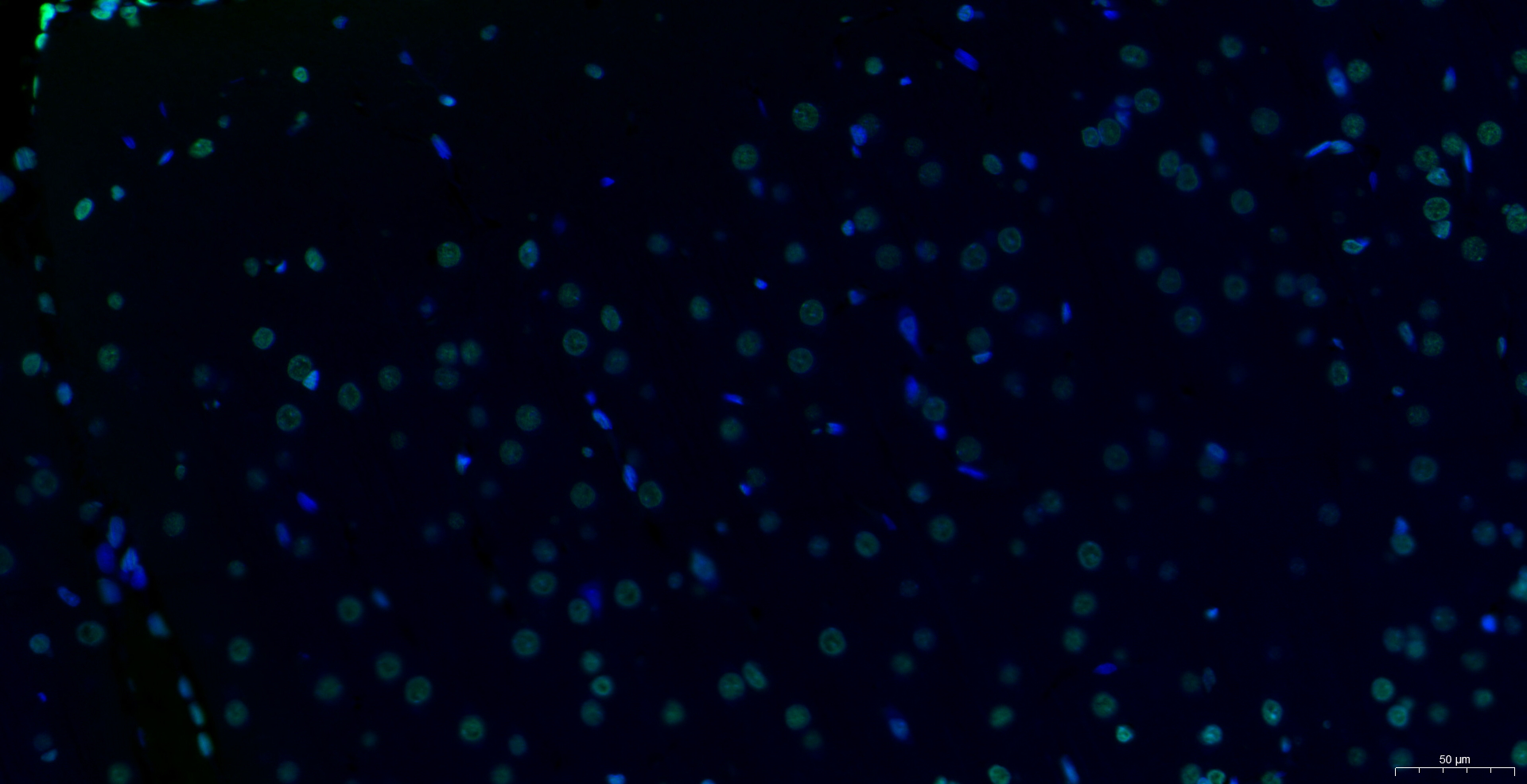

Supplement: S1 Raw data — (ZIP) [file pone.0305541.s002.zip › RAW DATA/FIG3/TUNEL COR/K-TUNEL_30.0x.jpg]

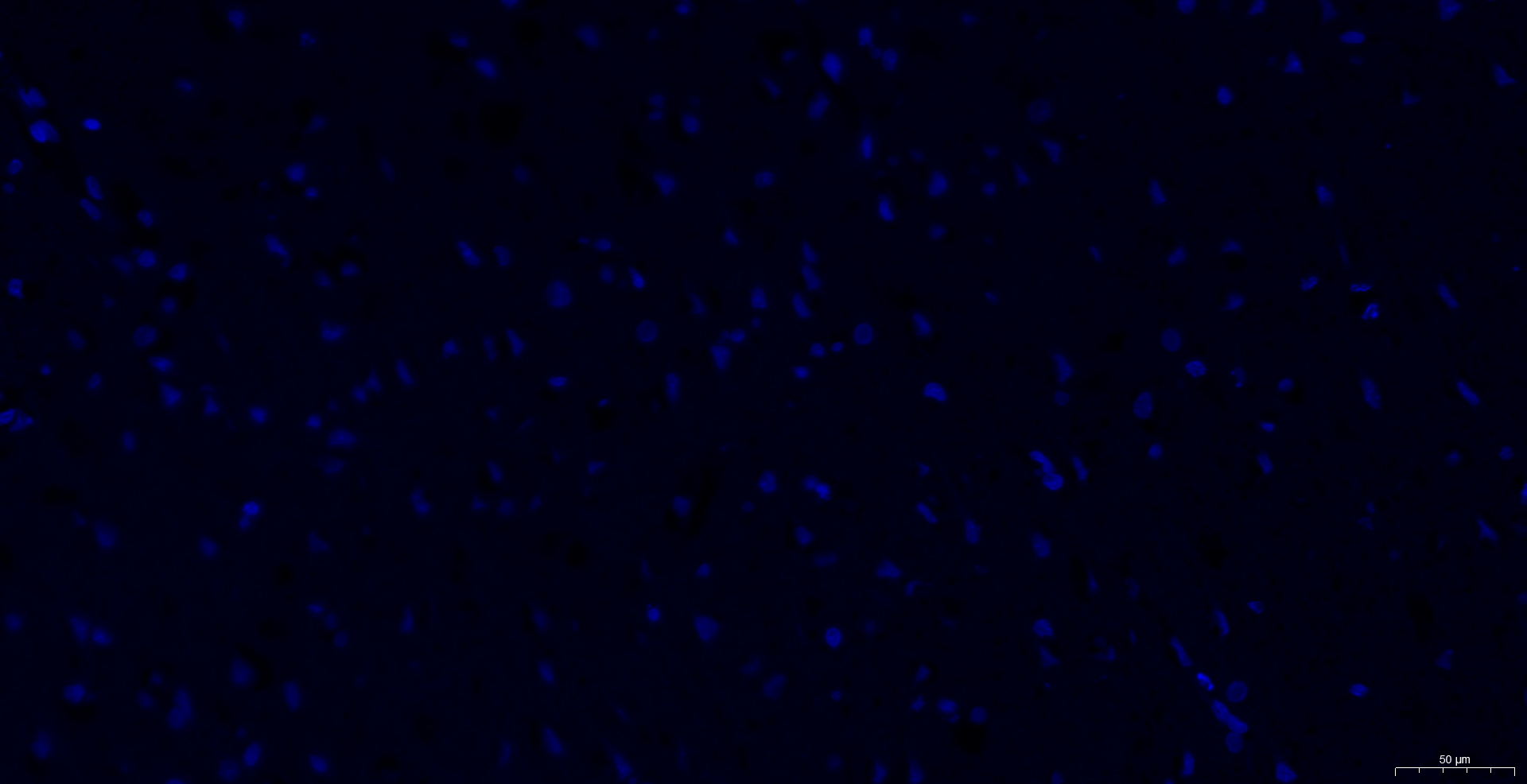

Supplement: S1 Raw data — (ZIP) [file pone.0305541.s002.zip › RAW DATA/FIG3/TUNEL COR/M-TUNEL_30.0x-1.jpg]

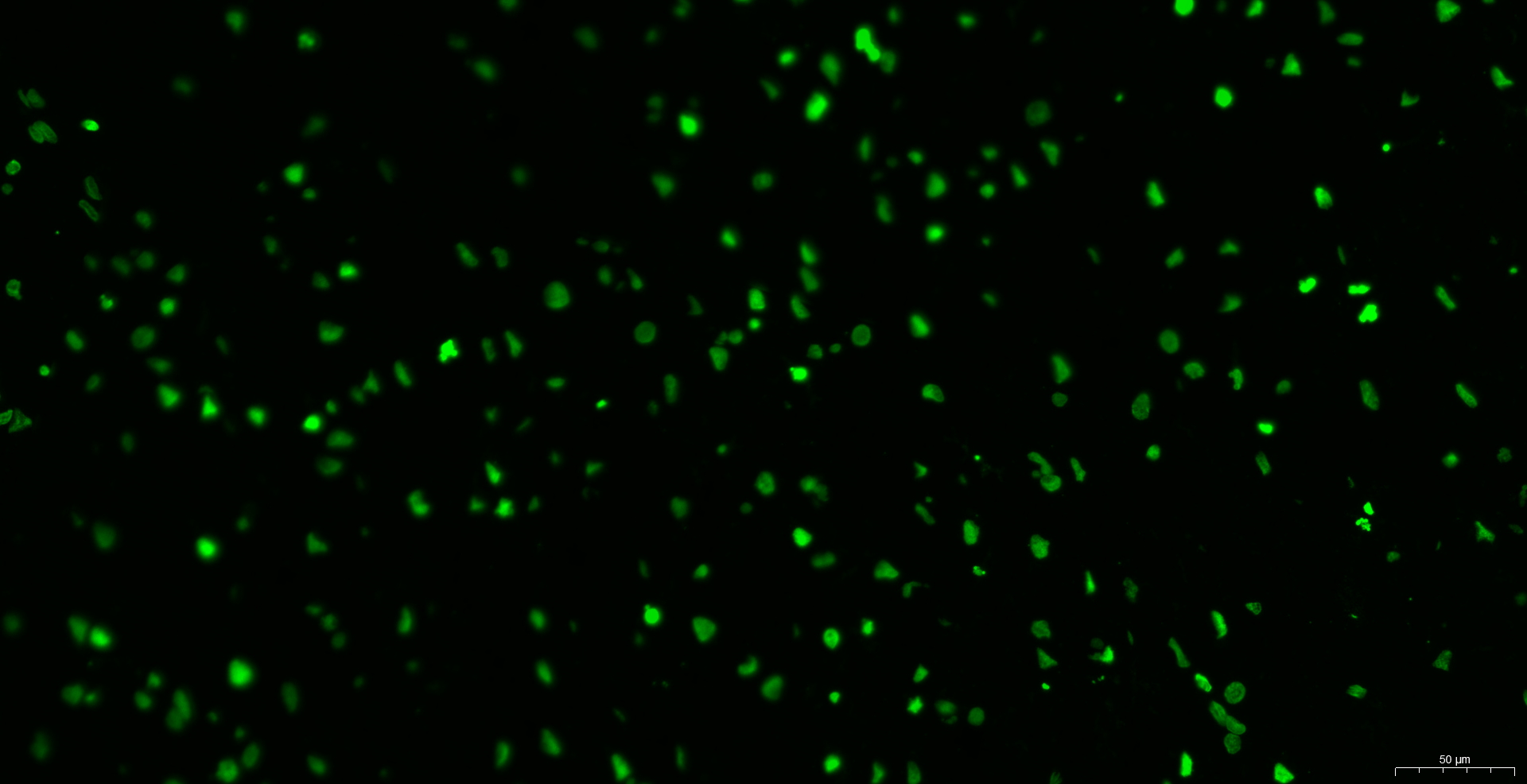

Supplement: S1 Raw data — (ZIP) [file pone.0305541.s002.zip › RAW DATA/FIG3/TUNEL COR/M-TUNEL_30.0x-2.jpg]

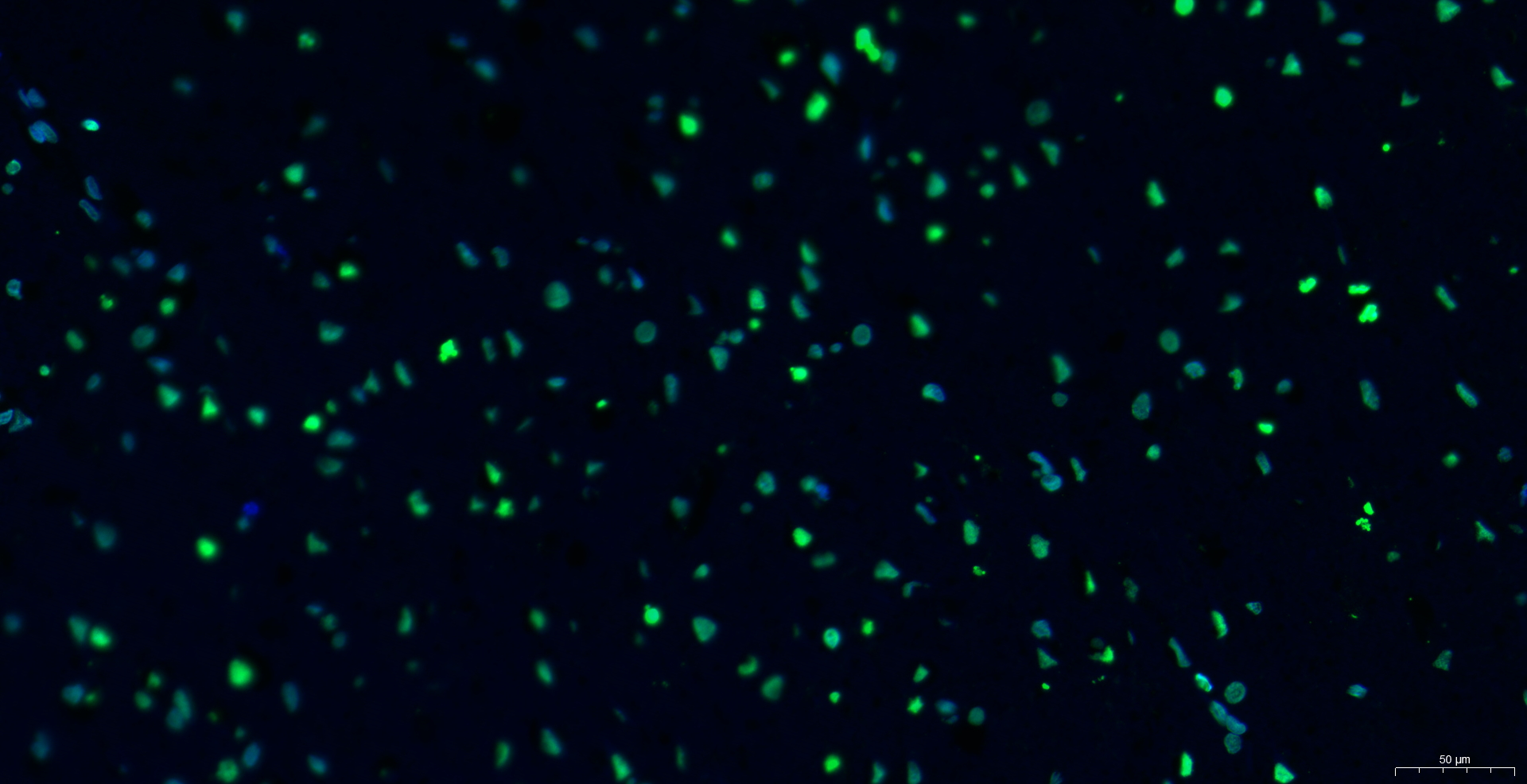

Supplement: S1 Raw data — (ZIP) [file pone.0305541.s002.zip › RAW DATA/FIG3/TUNEL COR/M-TUNEL_30.0x.jpg]

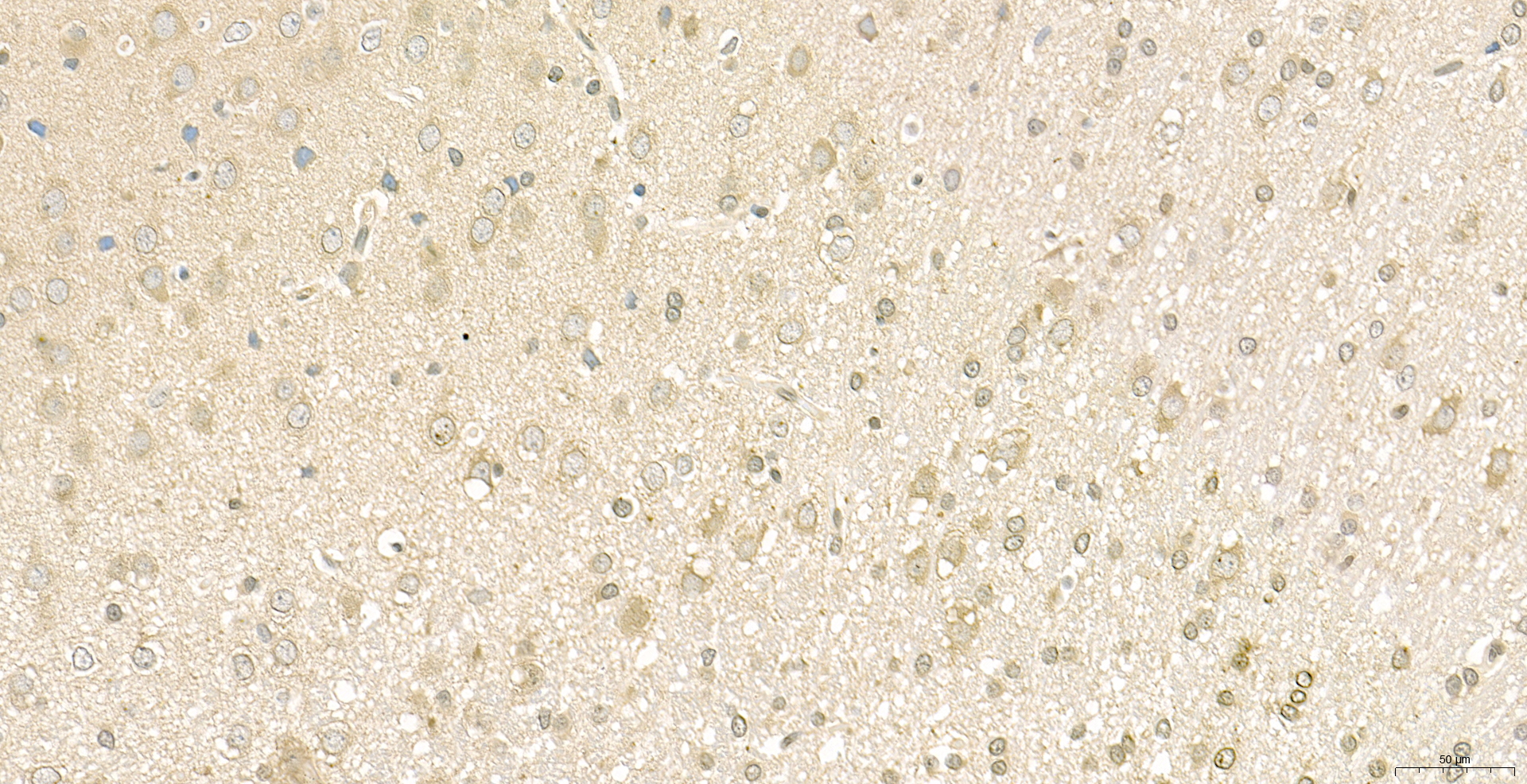

Supplement: S1 Raw data — (ZIP) [file pone.0305541.s002.zip › RAW DATA/FIG4/IL1B/10-100x_30.0x-PC.jpg]

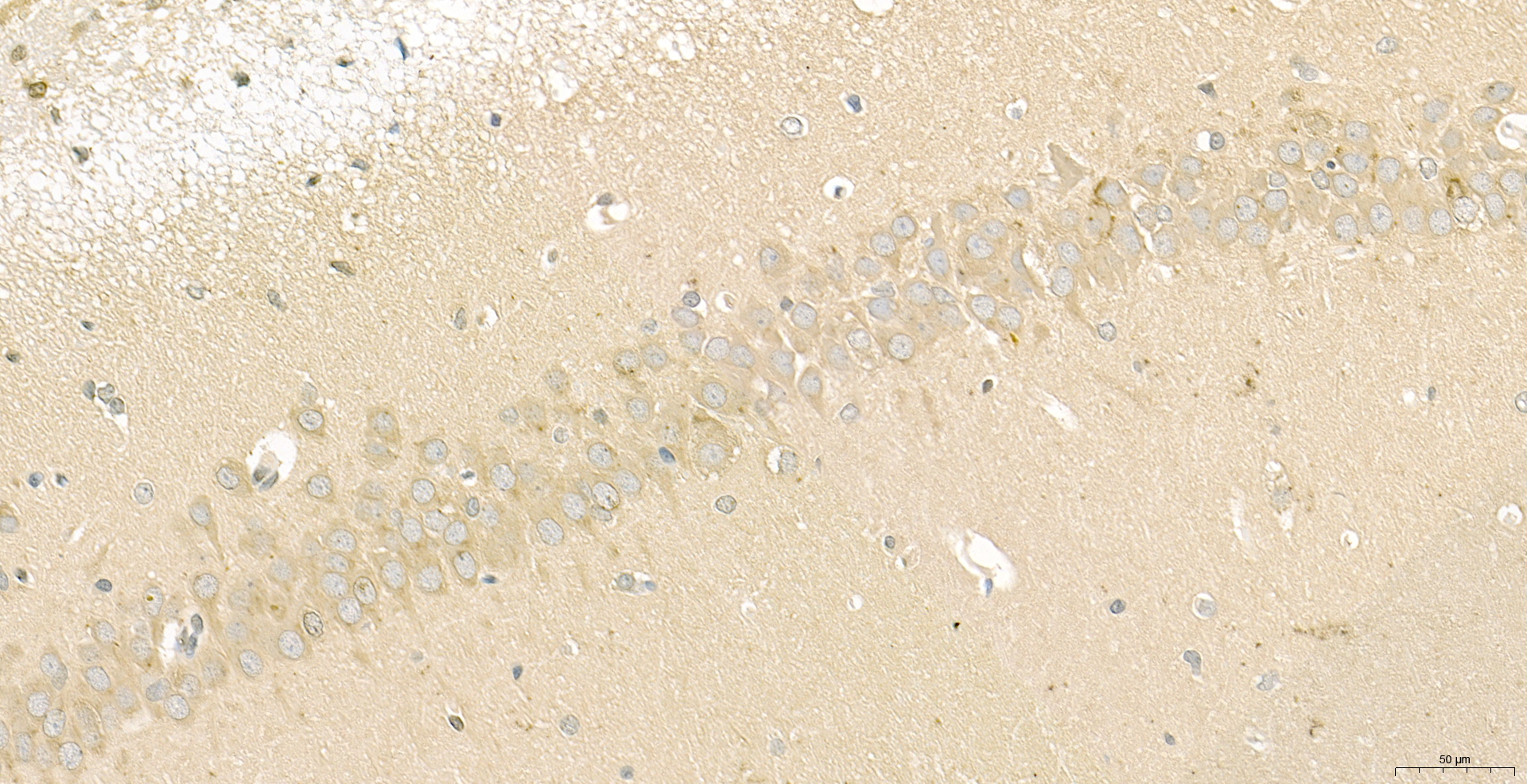

Supplement: S1 Raw data — (ZIP) [file pone.0305541.s002.zip › RAW DATA/FIG4/IL1B/10-100x_30.0x.jpg]

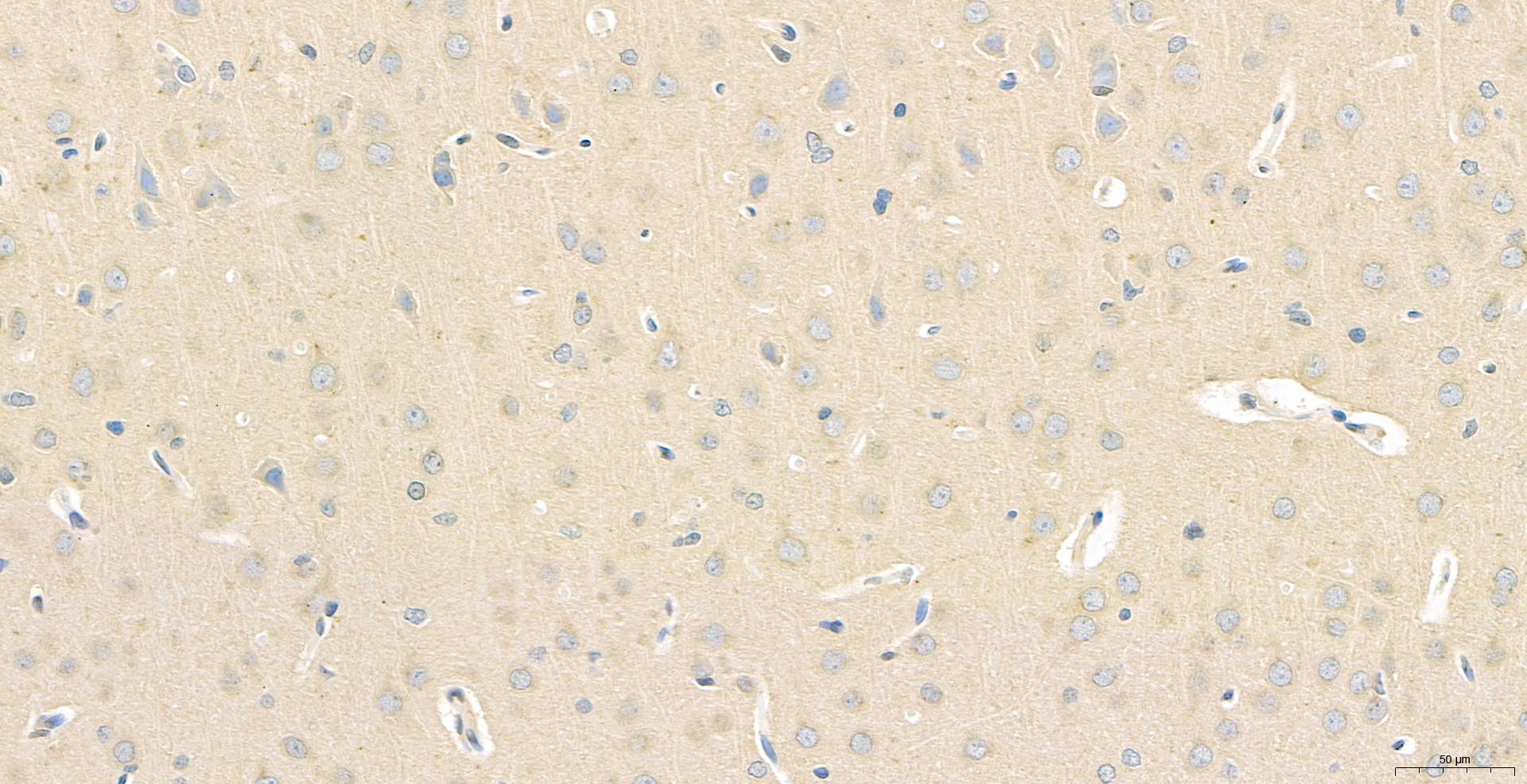

Supplement: S1 Raw data — (ZIP) [file pone.0305541.s002.zip › RAW DATA/FIG4/IL1B/20-100x_30.0x-PC.jpg]

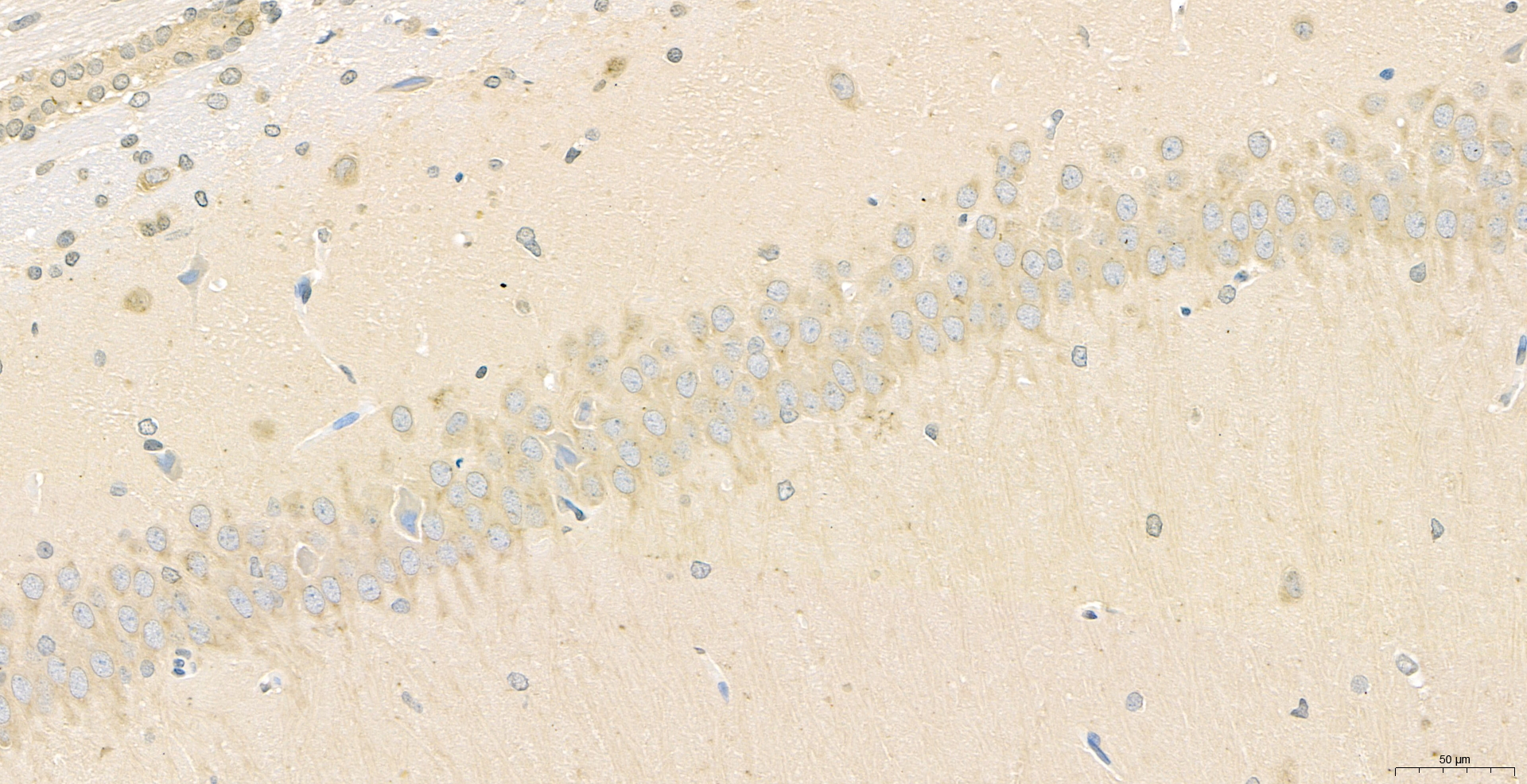

Supplement: S1 Raw data — (ZIP) [file pone.0305541.s002.zip › RAW DATA/FIG4/IL1B/20-100x_30.0x.jpg]

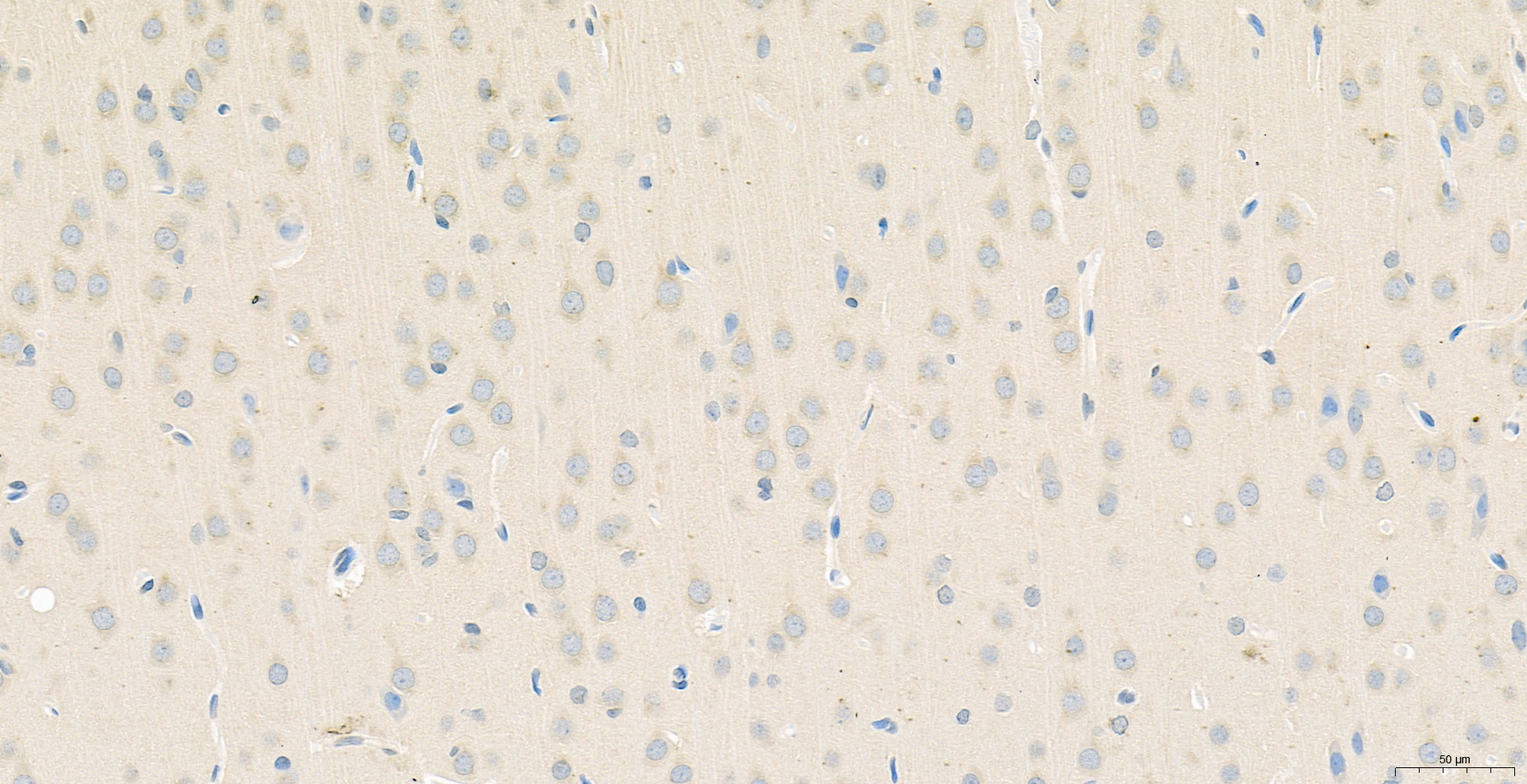

Supplement: S1 Raw data — (ZIP) [file pone.0305541.s002.zip › RAW DATA/FIG4/IL1B/K-100x_30.0x-PC.jpg]

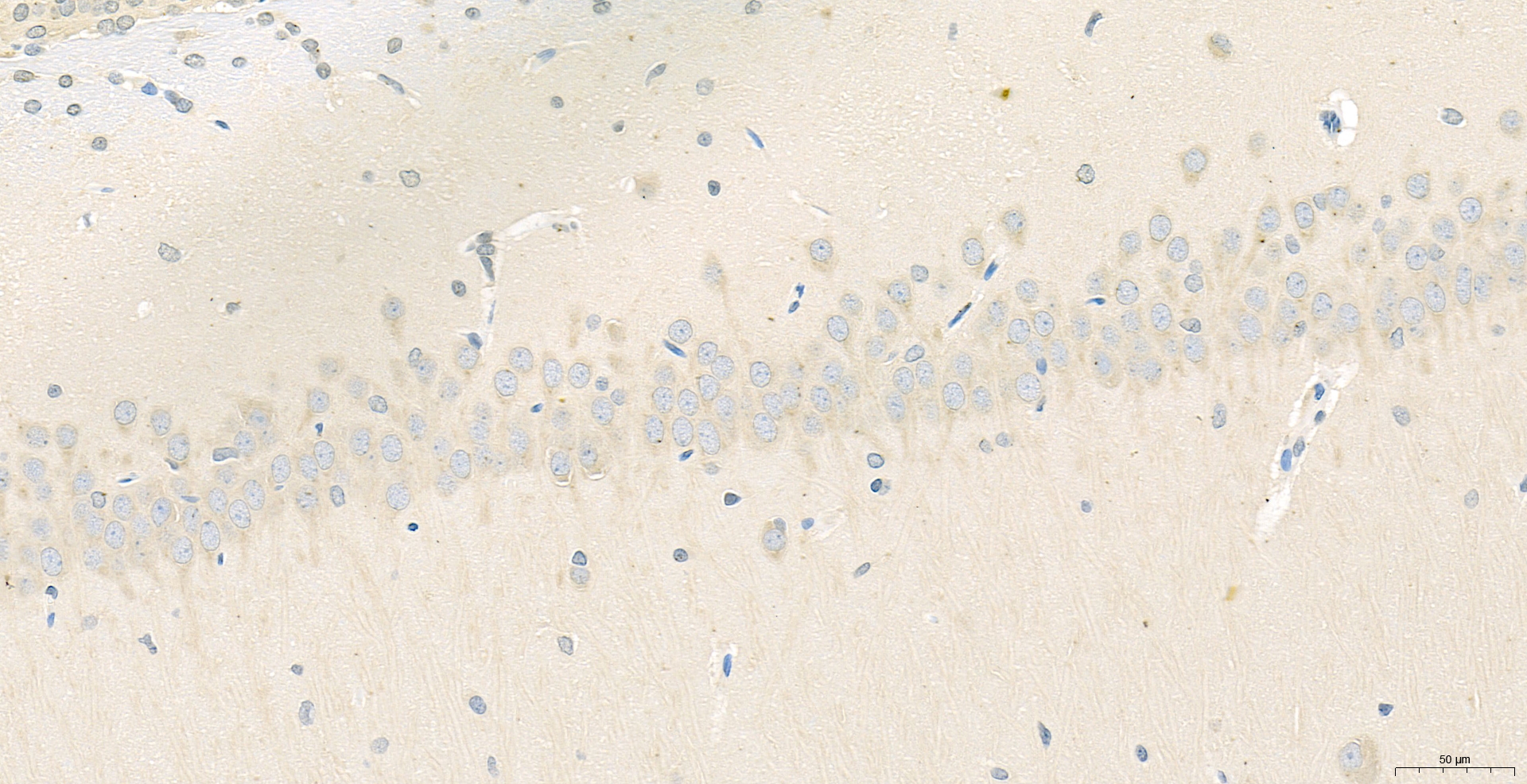

Supplement: S1 Raw data — (ZIP) [file pone.0305541.s002.zip › RAW DATA/FIG4/IL1B/K-100x_30.0x.jpg]

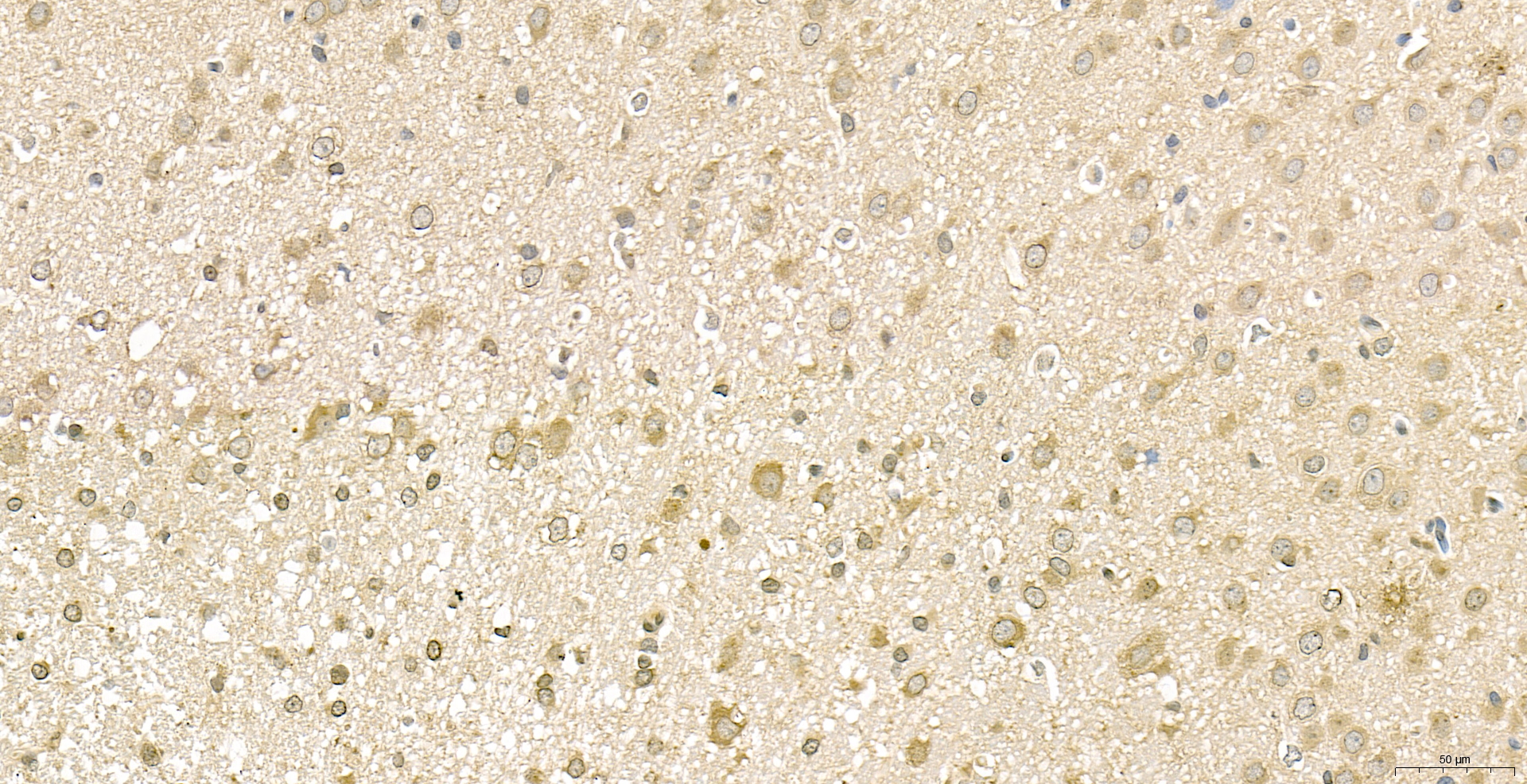

Supplement: S1 Raw data — (ZIP) [file pone.0305541.s002.zip › RAW DATA/FIG4/IL1B/M-100x_30.0x-PC.jpg]

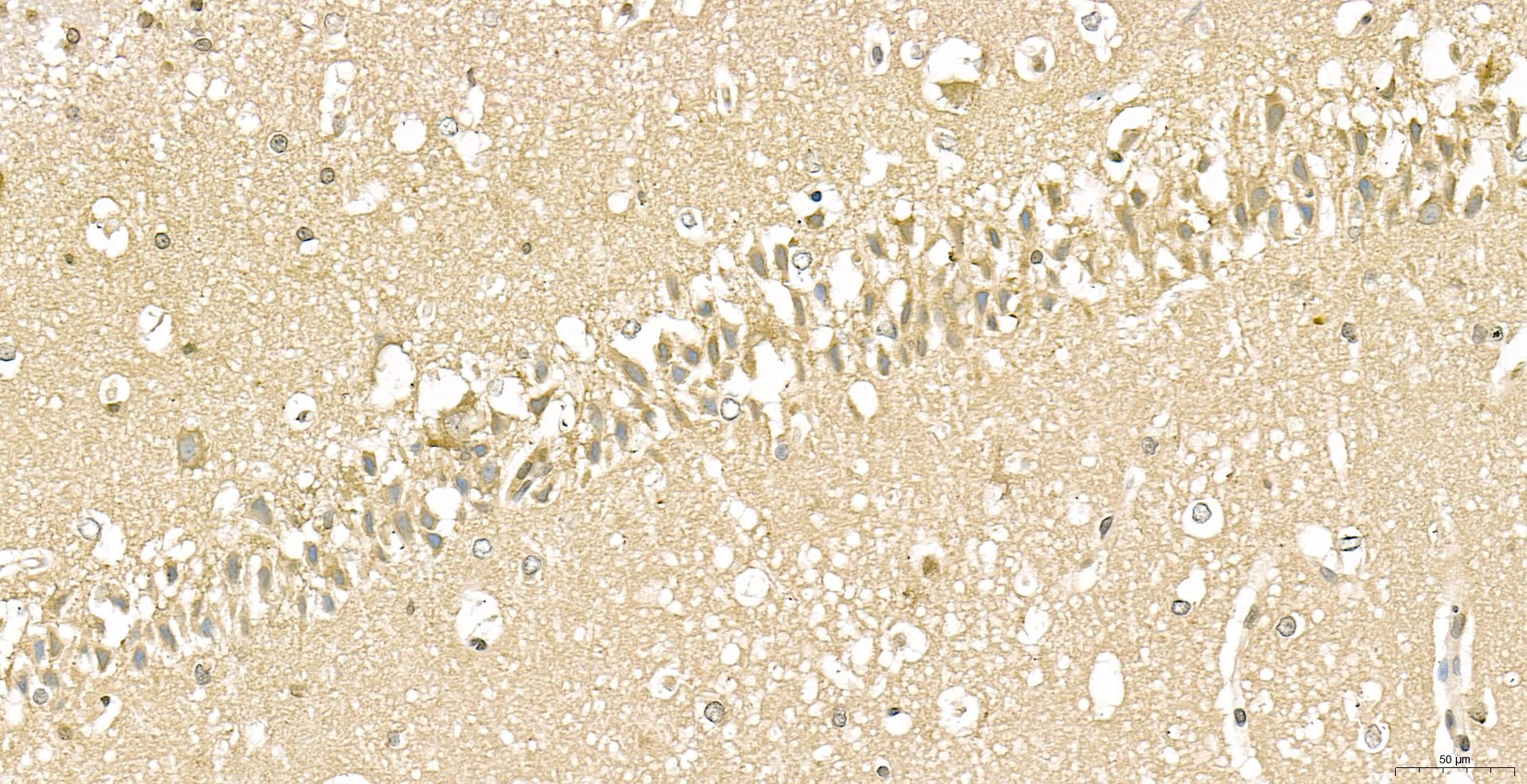

Supplement: S1 Raw data — (ZIP) [file pone.0305541.s002.zip › RAW DATA/FIG4/IL1B/M-100x_30.0x.jpg]

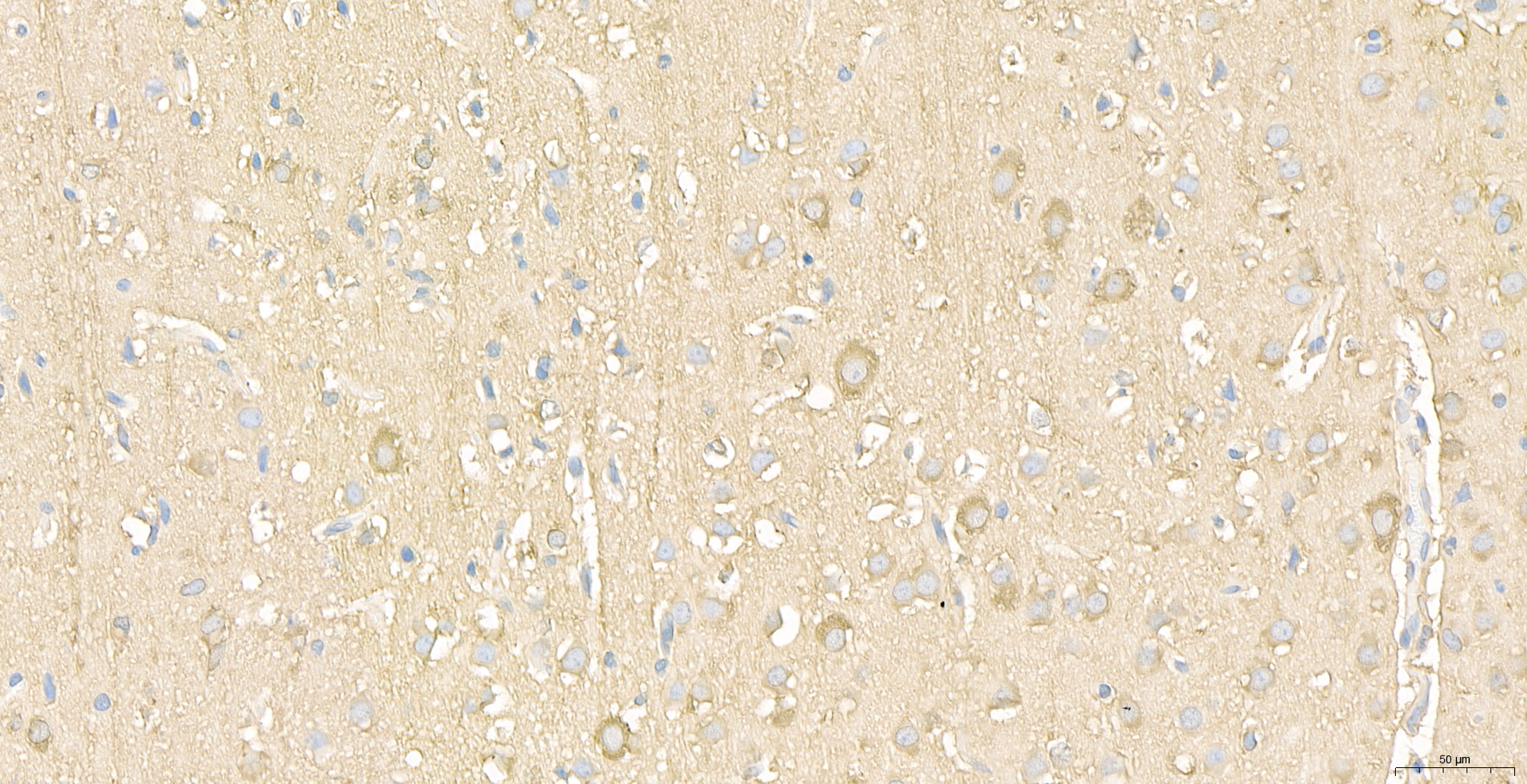

Supplement: S1 Raw data — (ZIP) [file pone.0305541.s002.zip › RAW DATA/FIG4/NLRP3/10-100x_30.0x-PC.jpg]

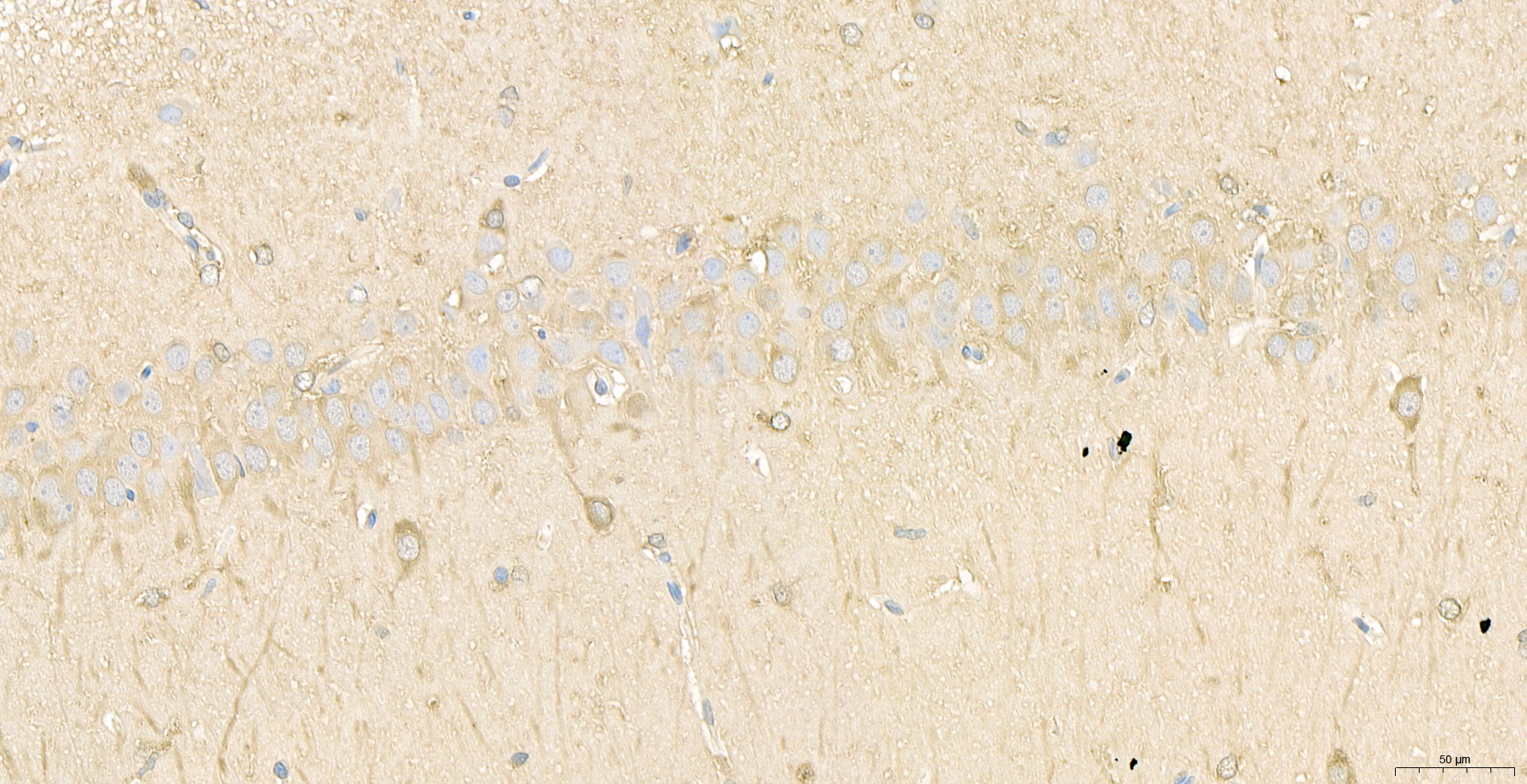

Supplement: S1 Raw data — (ZIP) [file pone.0305541.s002.zip › RAW DATA/FIG4/NLRP3/10-100x_30.0x.jpg]

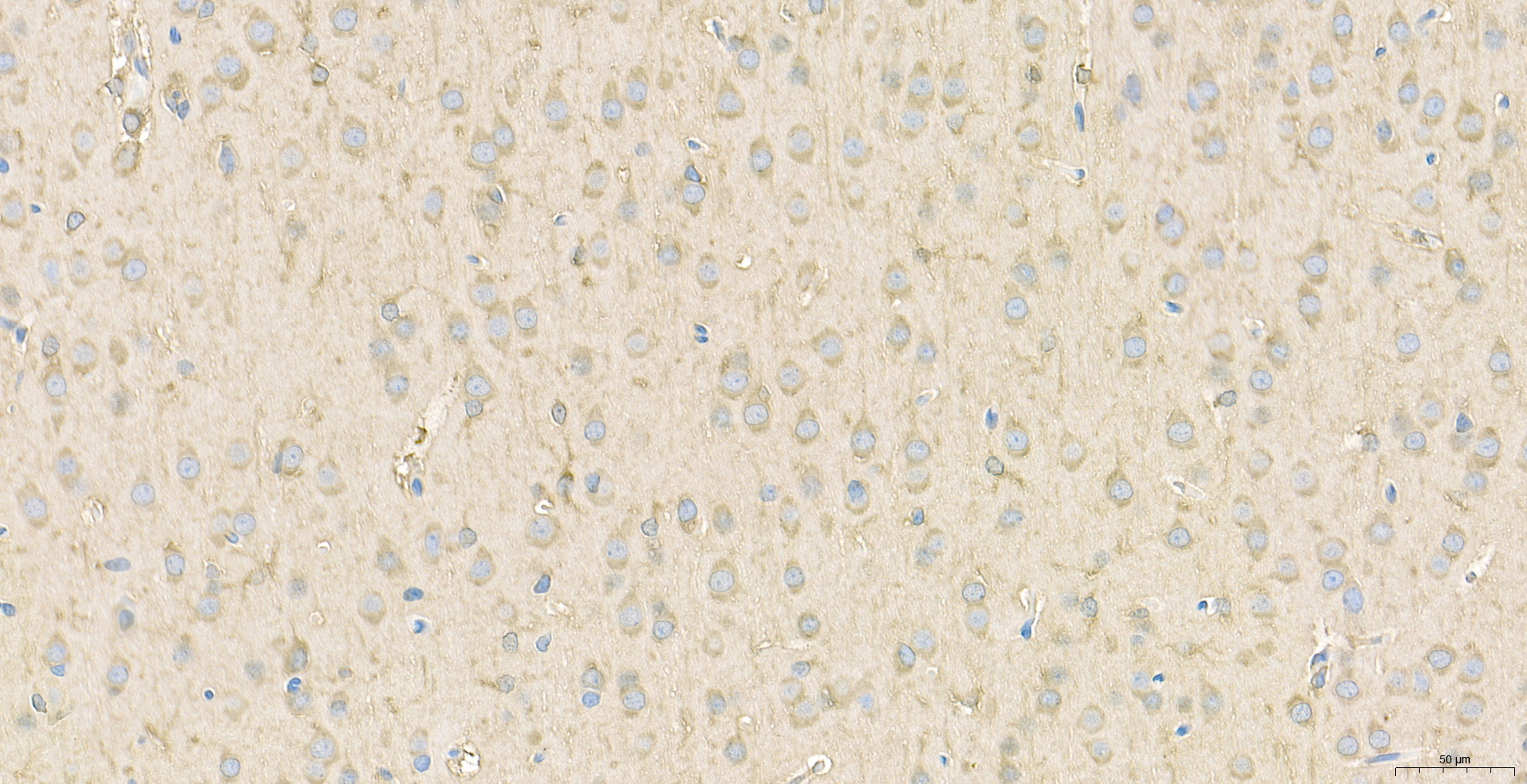

Supplement: S1 Raw data — (ZIP) [file pone.0305541.s002.zip › RAW DATA/FIG4/NLRP3/20-100x_30.0x-PC.jpg]

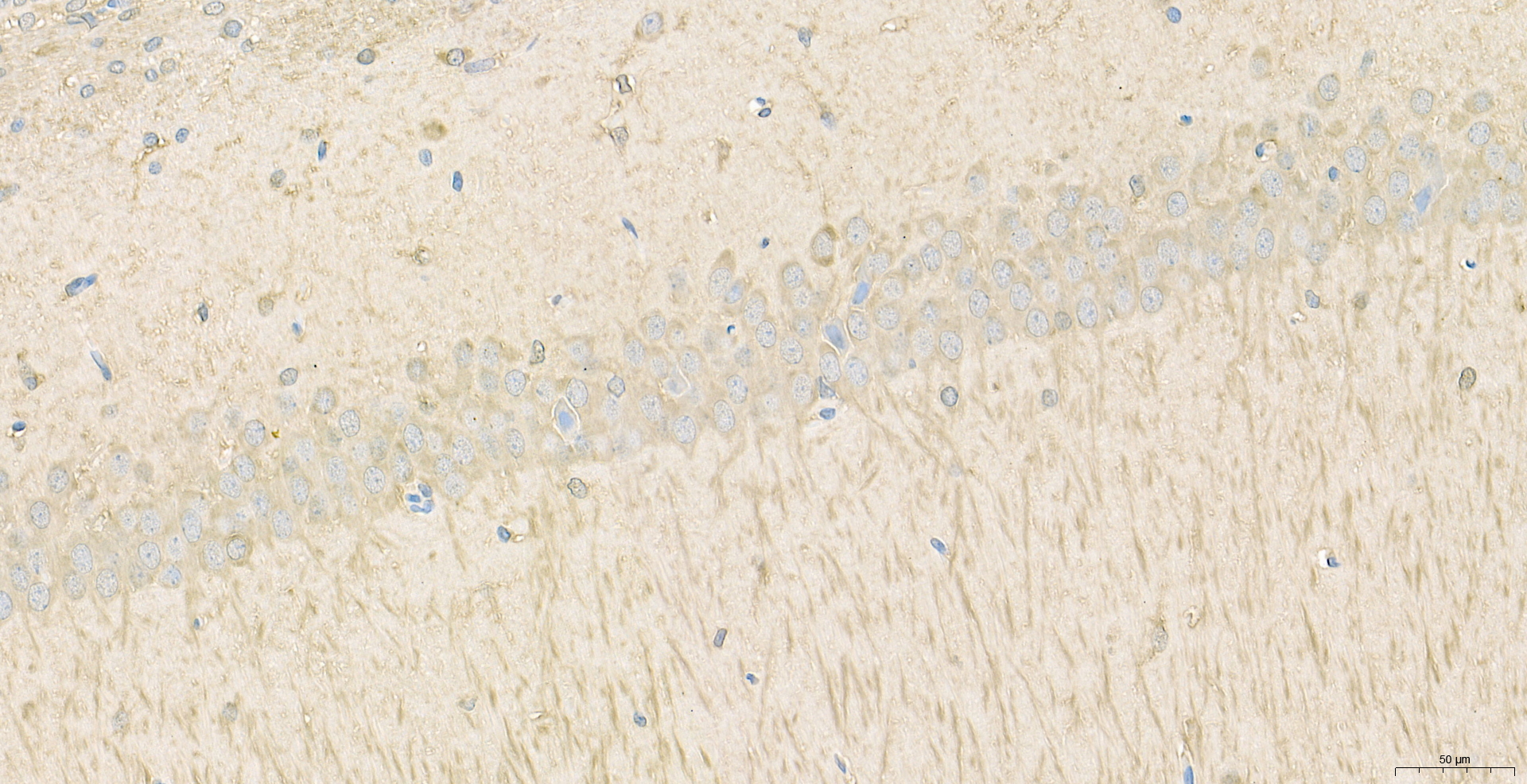

Supplement: S1 Raw data — (ZIP) [file pone.0305541.s002.zip › RAW DATA/FIG4/NLRP3/20-100x_30.0x.jpg]

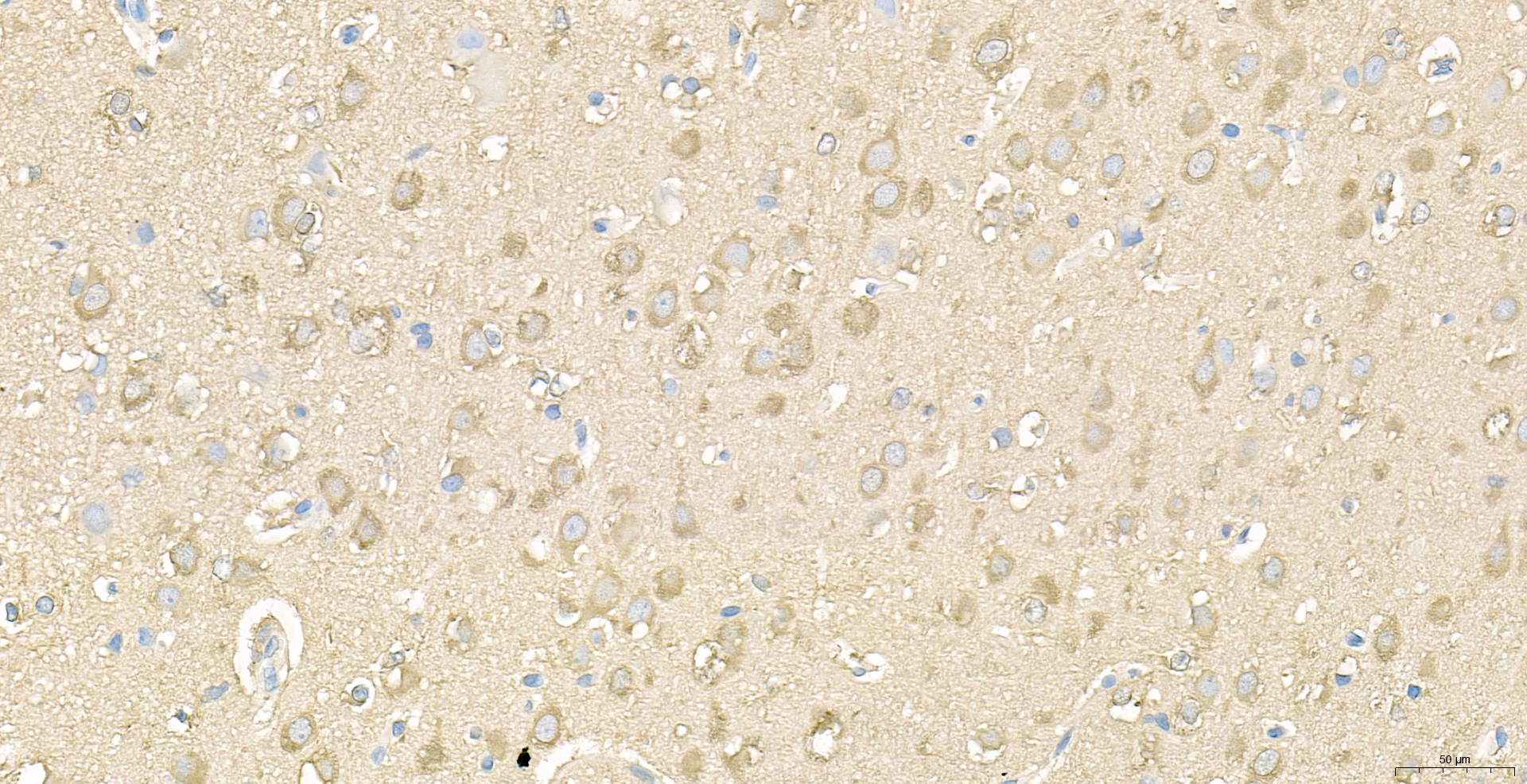

Supplement: S1 Raw data — (ZIP) [file pone.0305541.s002.zip › RAW DATA/FIG4/NLRP3/M-100x_30.0x-PC.jpg]

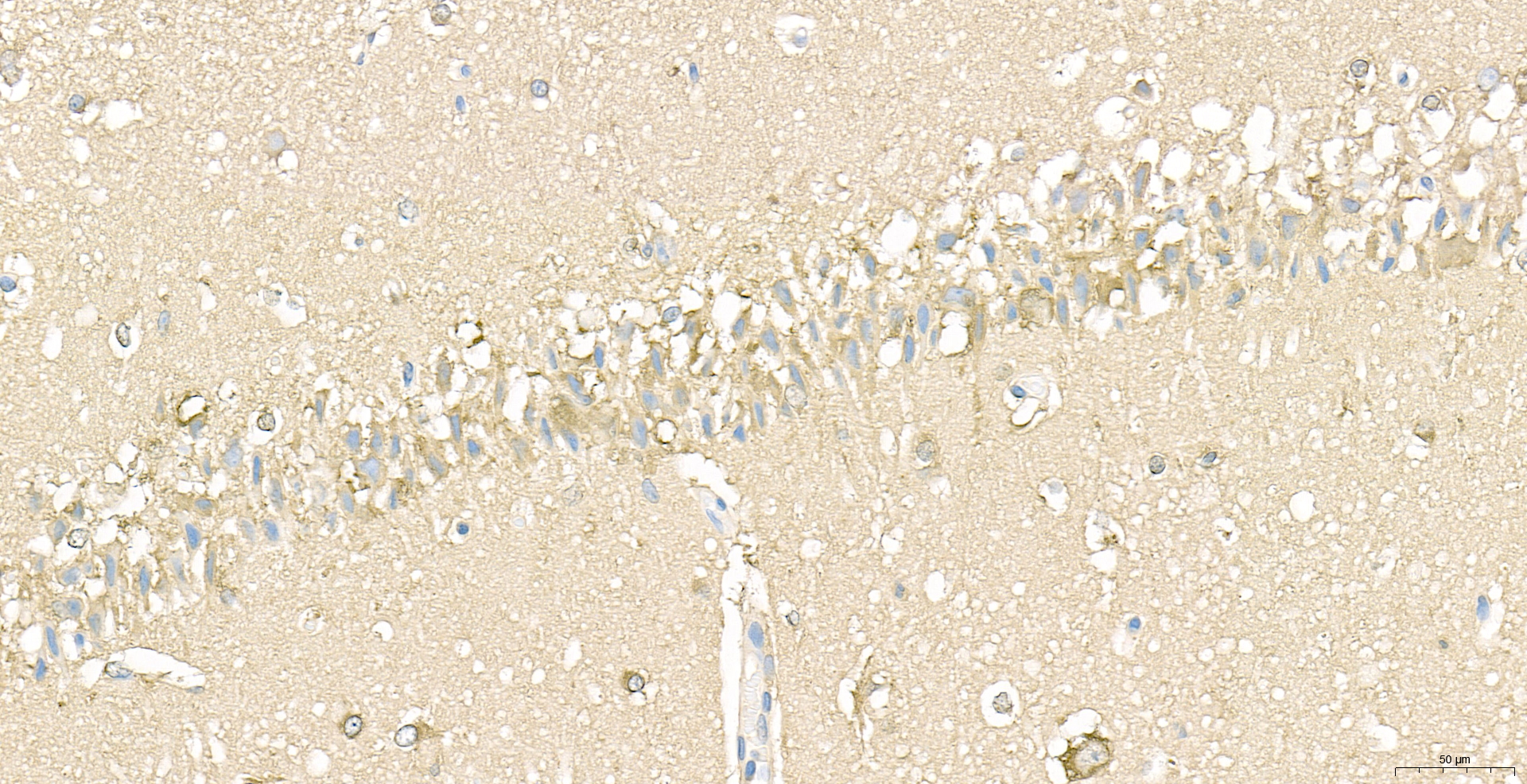

Supplement: S1 Raw data — (ZIP) [file pone.0305541.s002.zip › RAW DATA/FIG4/NLRP3/M-100x_30.0x.jpg]

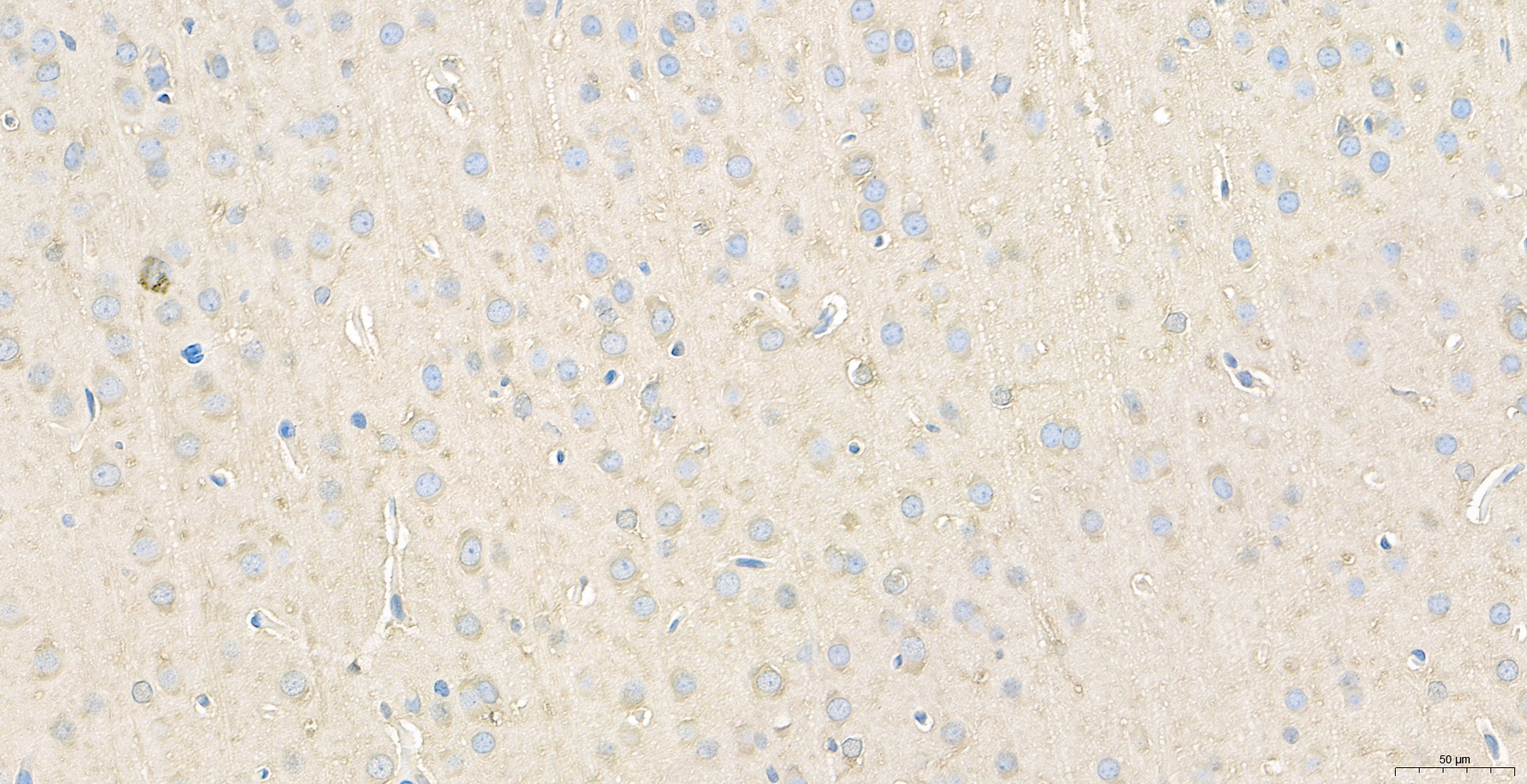

Supplement: S1 Raw data — (ZIP) [file pone.0305541.s002.zip › RAW DATA/FIG4/NLRP3/k-100x_30.0x-PC.jpg]

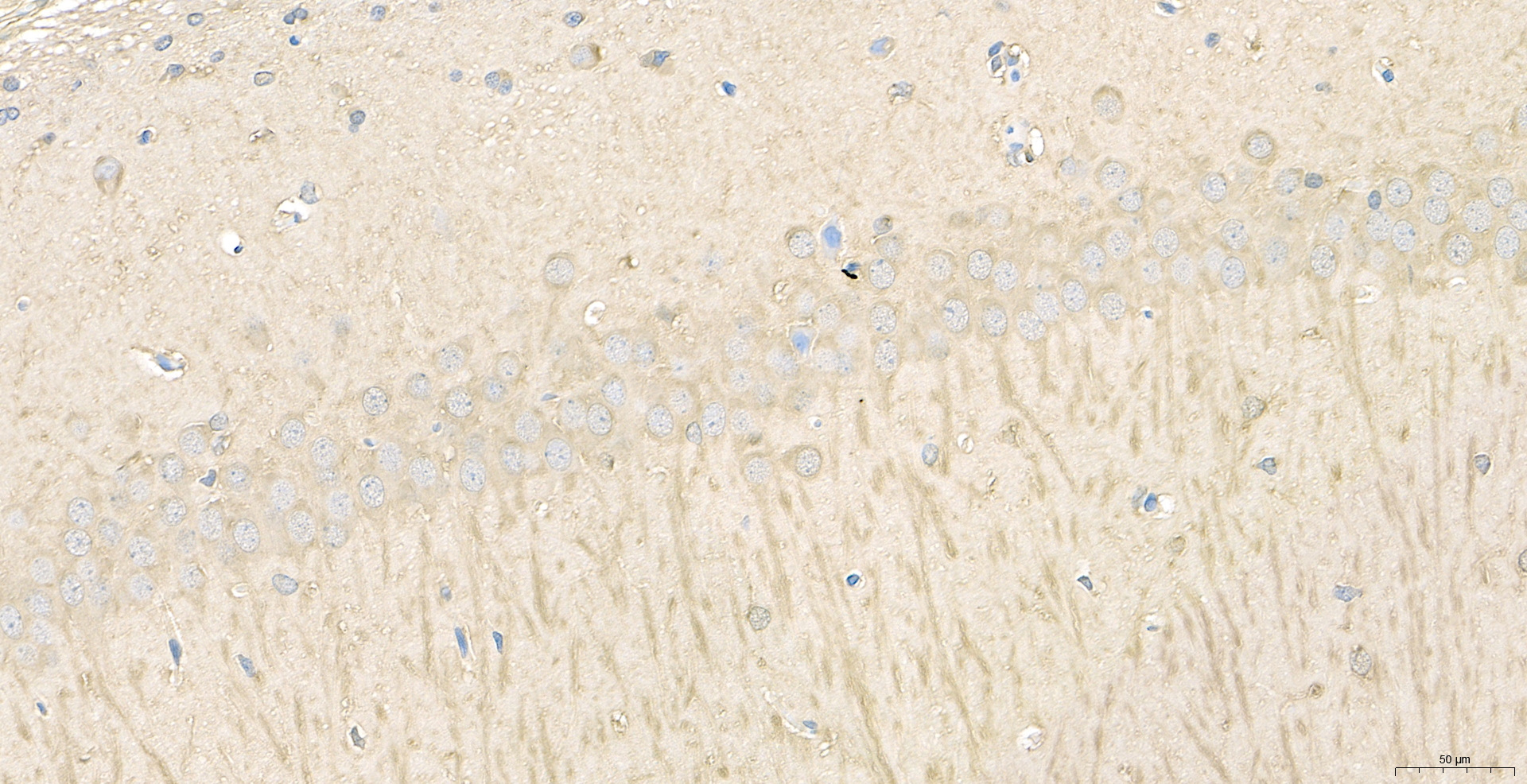

Supplement: S1 Raw data — (ZIP) [file pone.0305541.s002.zip › RAW DATA/FIG4/NLRP3/k-100x_30.0x.jpg]

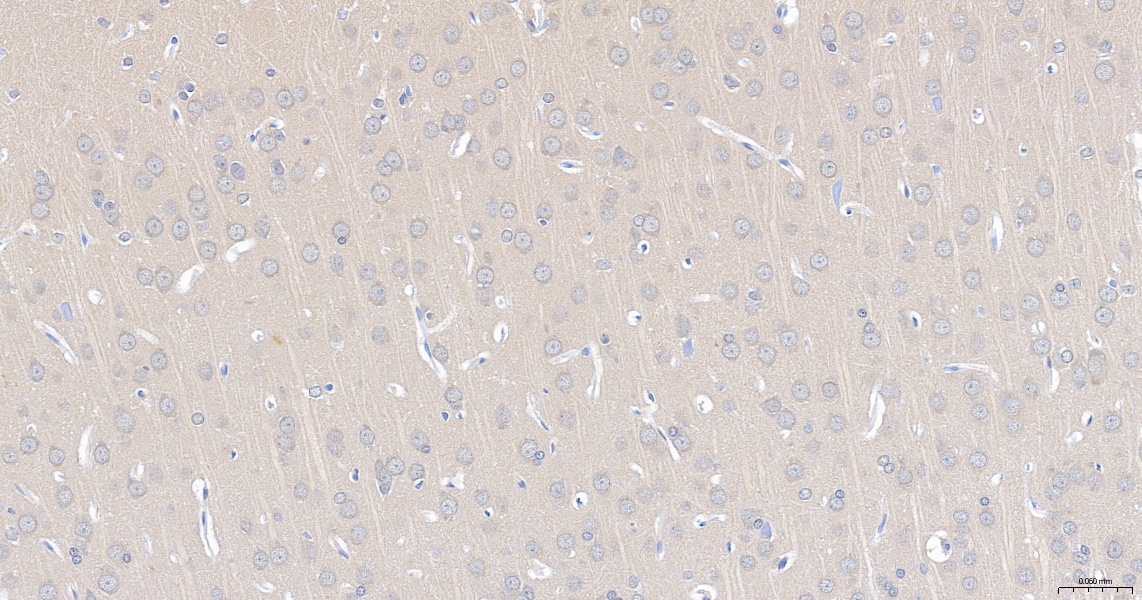

Supplement: S1 Raw data — (ZIP) [file pone.0305541.s002.zip › RAW DATA/FIG4/─╘NFKB/10-100X_25.0x.jpg]

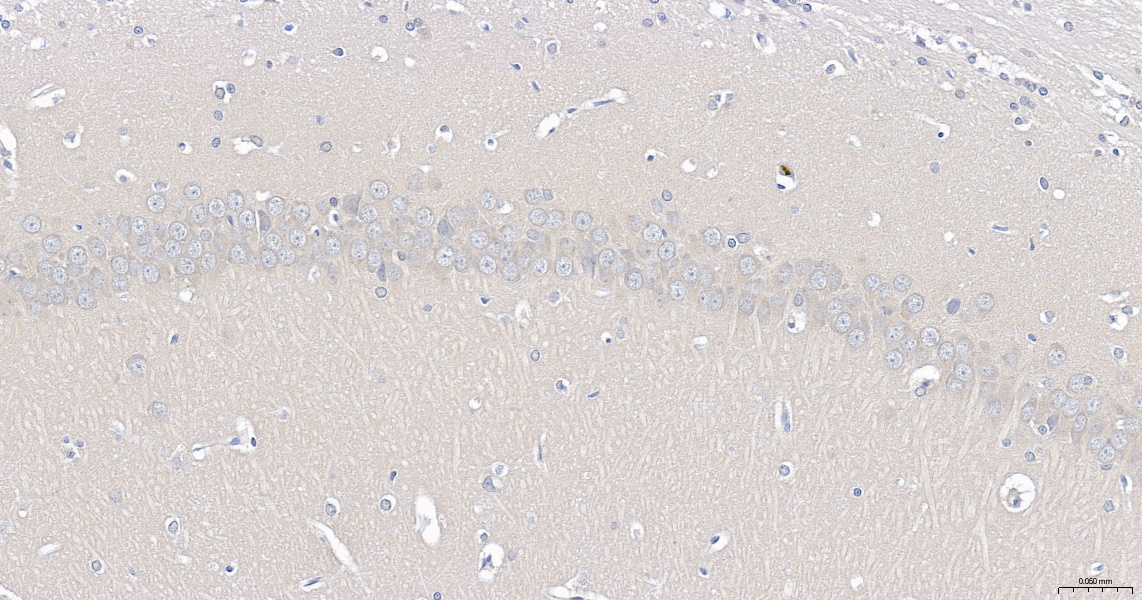

Supplement: S1 Raw data — (ZIP) [file pone.0305541.s002.zip › RAW DATA/FIG4/─╘NFKB/10-100X_25.0x1.jpg]

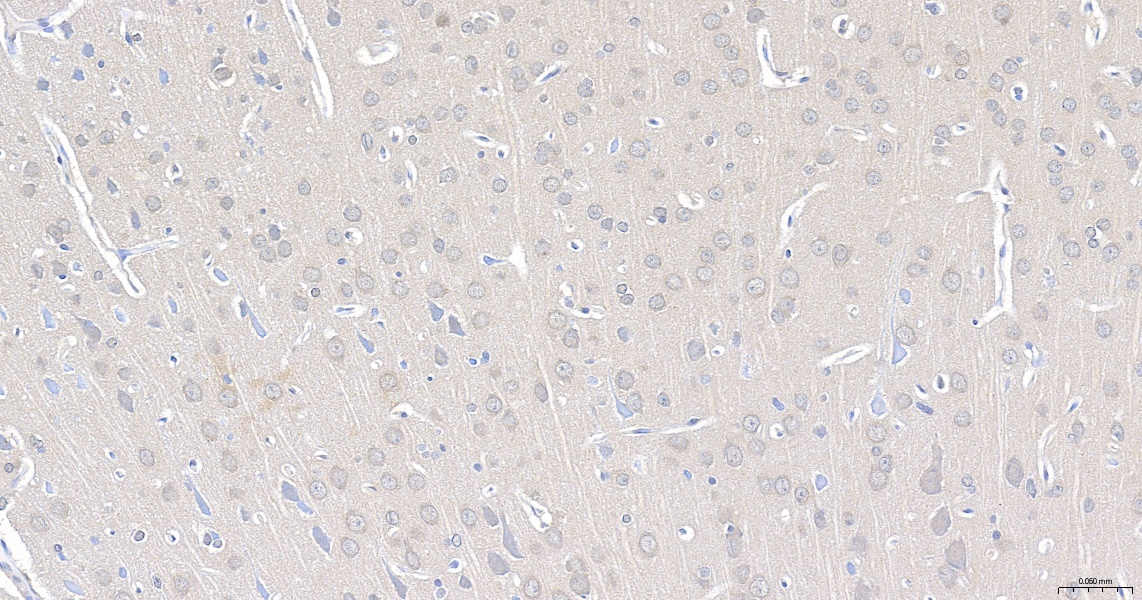

Supplement: S1 Raw data — (ZIP) [file pone.0305541.s002.zip › RAW DATA/FIG4/─╘NFKB/20-100X_25.0x.jpg]

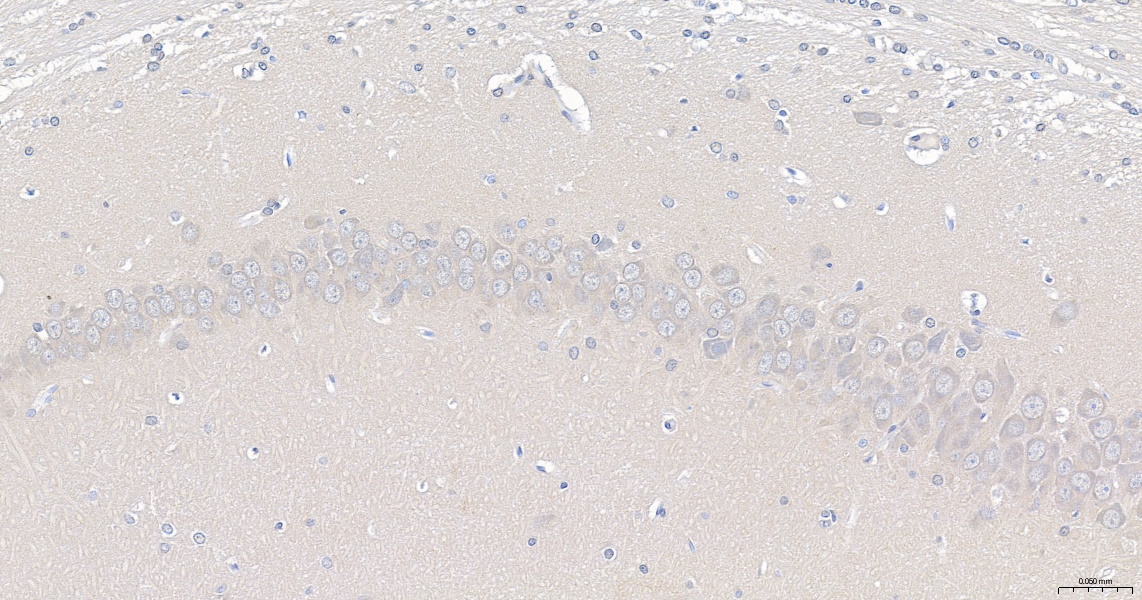

Supplement: S1 Raw data — (ZIP) [file pone.0305541.s002.zip › RAW DATA/FIG4/─╘NFKB/20-100X_25.0x1.jpg]

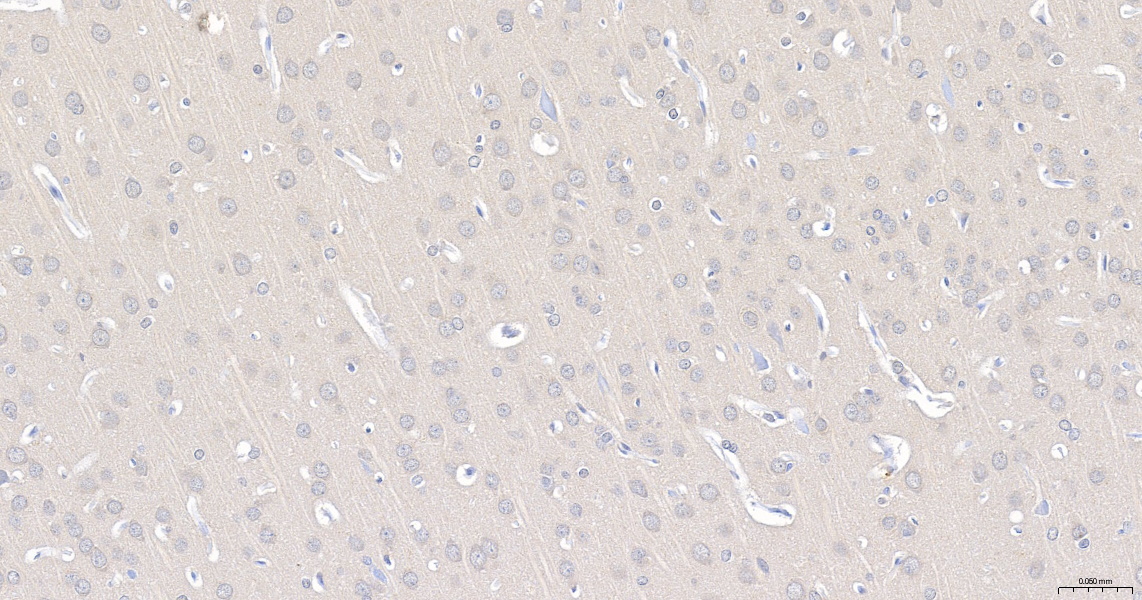

Supplement: S1 Raw data — (ZIP) [file pone.0305541.s002.zip › RAW DATA/FIG4/─╘NFKB/K-100X_25.0x.jpg]

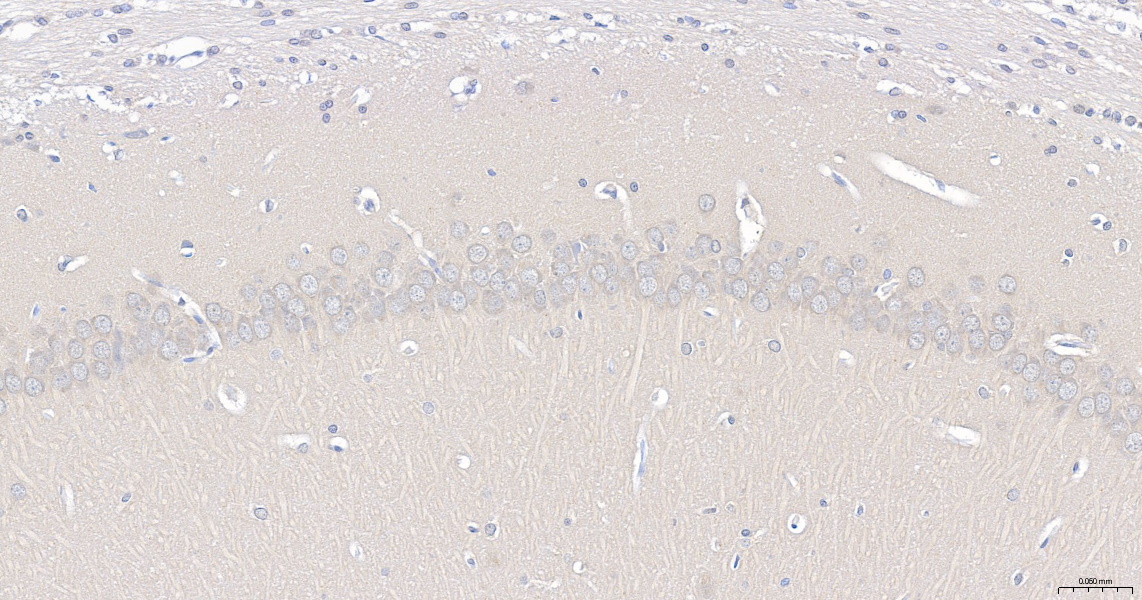

Supplement: S1 Raw data — (ZIP) [file pone.0305541.s002.zip › RAW DATA/FIG4/─╘NFKB/K-100X_25.0x1.jpg]

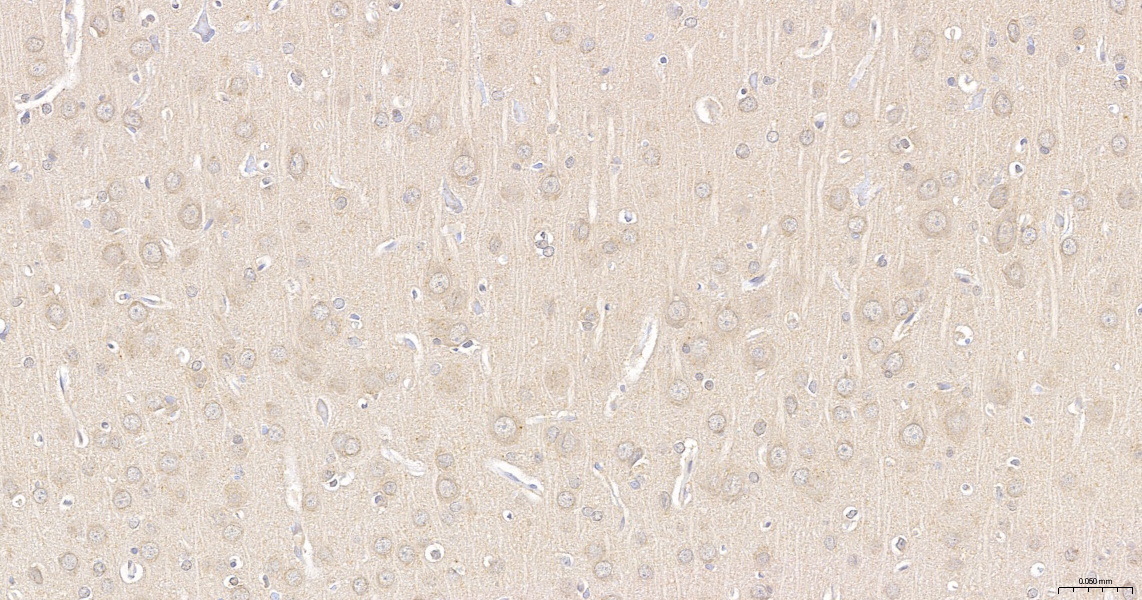

Supplement: S1 Raw data — (ZIP) [file pone.0305541.s002.zip › RAW DATA/FIG4/─╘NFKB/M-100X_25.0x.jpg]

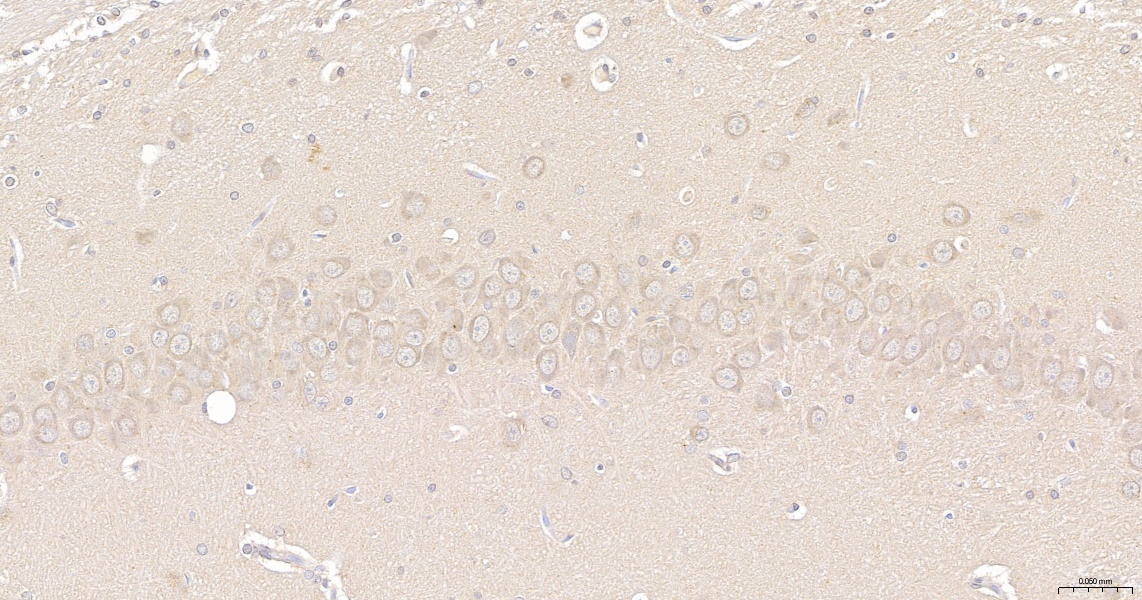

Supplement: S1 Raw data — (ZIP) [file pone.0305541.s002.zip › RAW DATA/FIG4/─╘NFKB/M-100X_25.0x1.jpg]

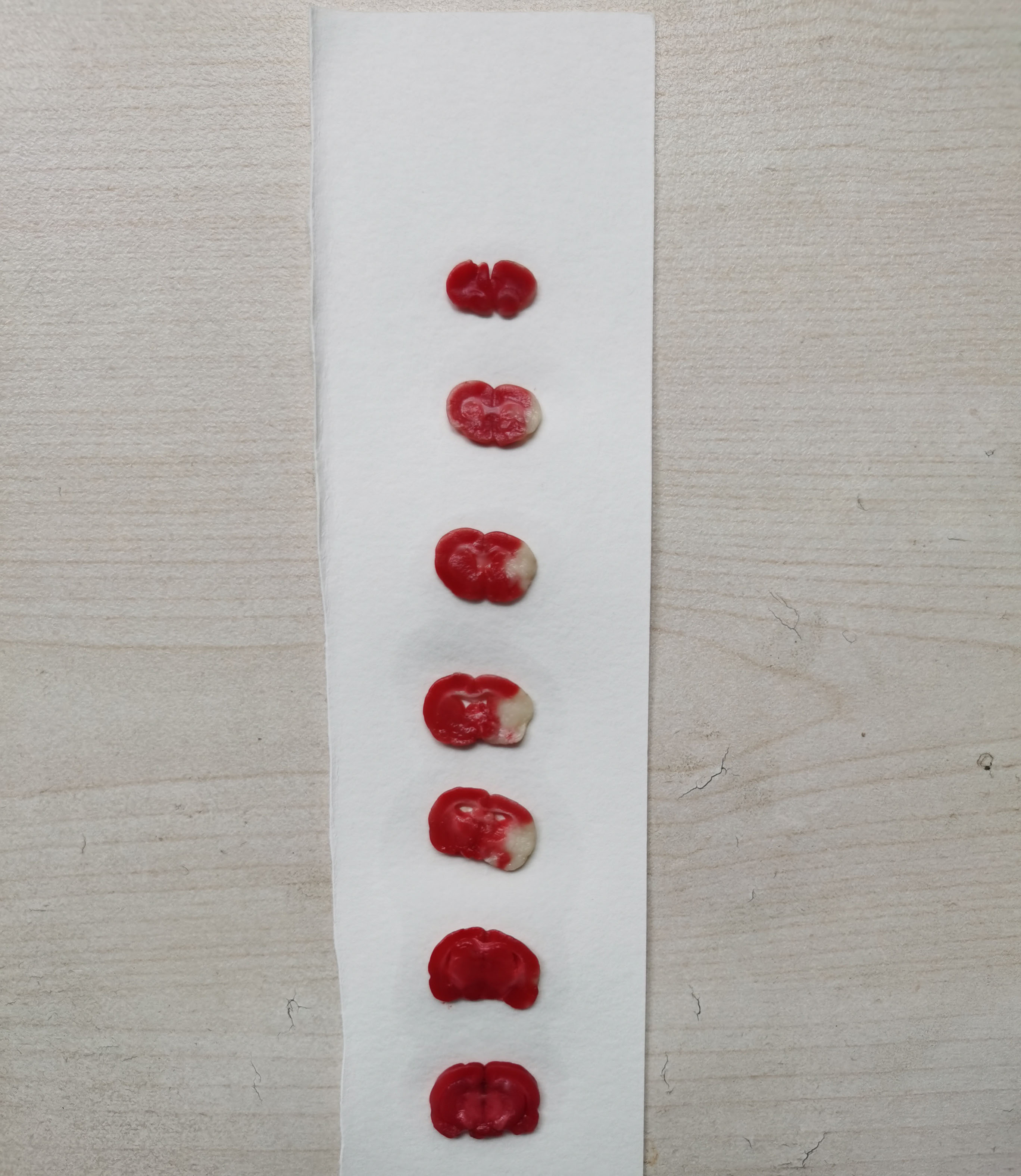

Supplement: S1 Raw data — (ZIP) [file pone.0305541.s002.zip › RAW DATA/FIG6/TTC/AAV-cont+FB.jpg]

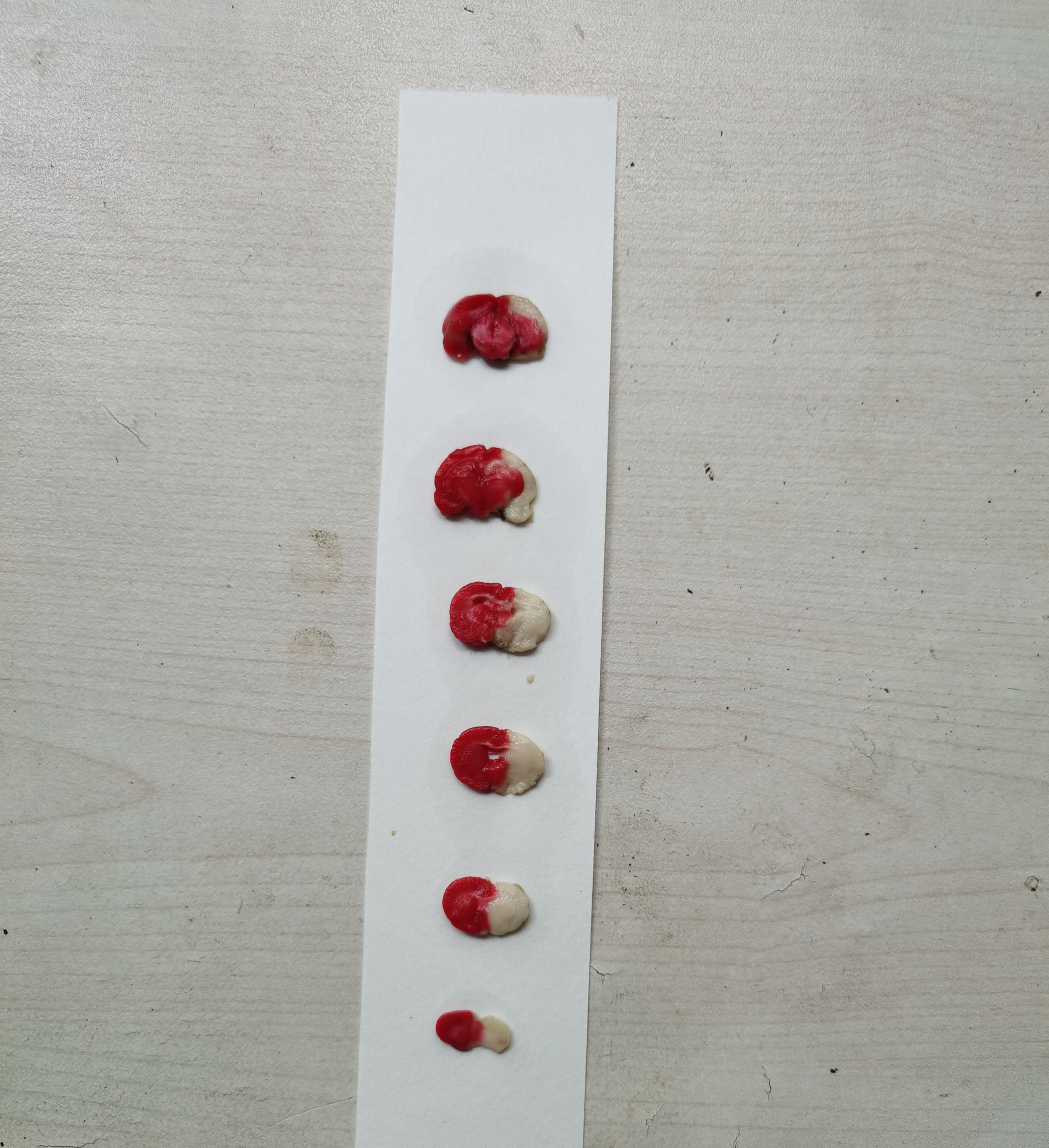

Supplement: S1 Raw data — (ZIP) [file pone.0305541.s002.zip › RAW DATA/FIG6/TTC/AAV-cont.jpg]

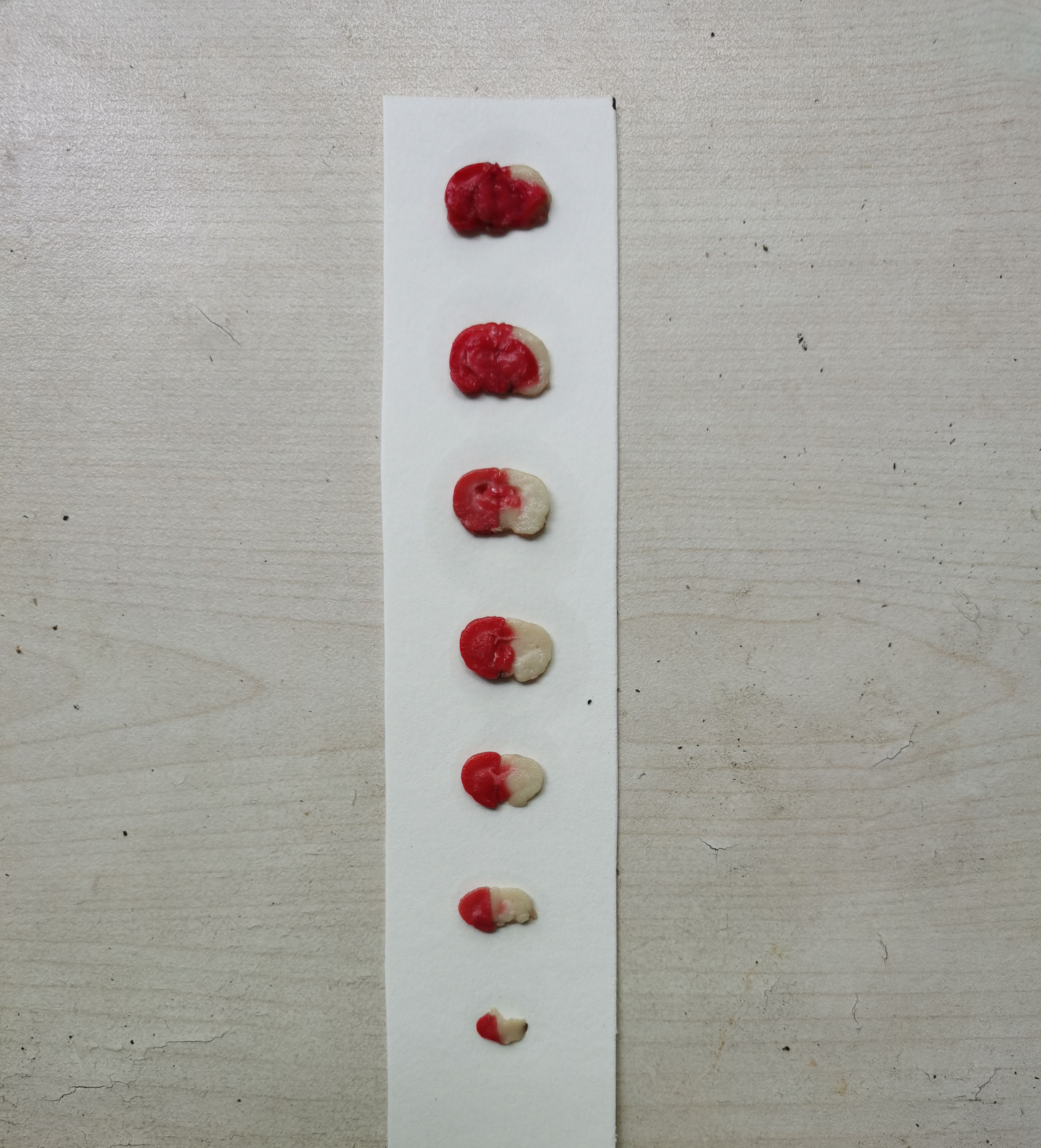

Supplement: S1 Raw data — (ZIP) [file pone.0305541.s002.zip › RAW DATA/FIG6/TTC/AAV-sirt1+FB.jpg]

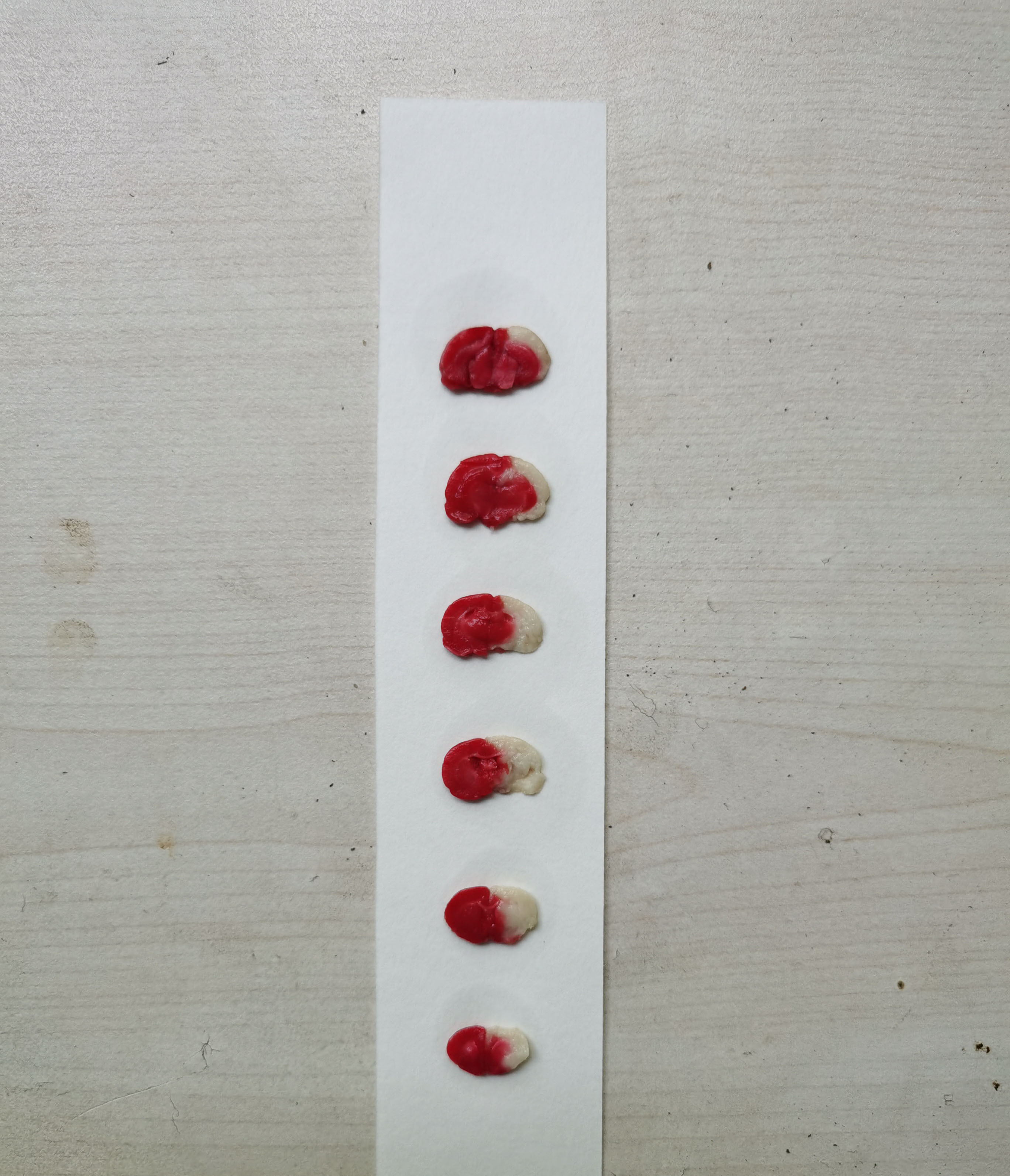

Supplement: S1 Raw data — (ZIP) [file pone.0305541.s002.zip › RAW DATA/FIG6/TTC/AAV-sirt1.jpg]
